# Supplementary material for: Identification of BiP as a temperature sensor mediating temperature-induced germline sex reversal in C. elegans
Source: EMBO J. 2024 Aug 12;43(18):4020–48. doi: 10.1038/s44318-024-00197-z (PMC11405683; doi:10.1038/s44318-024-00197-z)
Supplement: Supplementary file 2 — Appendix [file 44318_2024_197_MOESM2_ESM.pdf]

Identification of BiP as a temperature sensor mediating temperature-induced germline sex reversal in C. elegans

Jing Shi et al

Table of Contents:

Appendix Figure S1

Appendix Figure S2

Appendix Figure S3

Appendix Figure S4

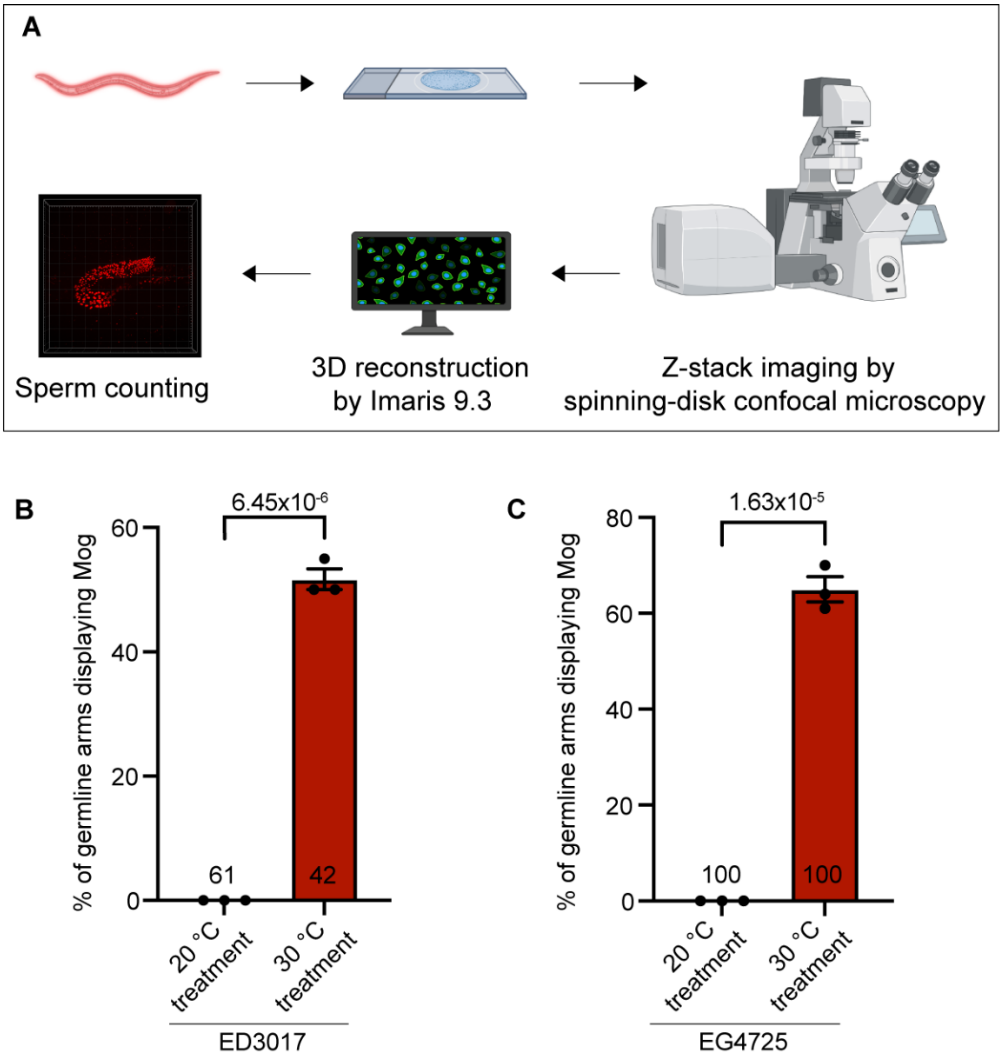

Appendix Figure S1

Warmer temperature promotes excess sperm production in ED3017 and EG4725 *C. elegans*.

(A) Workflow for quantifying sperm numbers. After the indicated temperature treatment, DE90 worms carrying the sperm marker transgene, and other worms stained with DAPI, were transferred onto slides. Z-stack images of the germline were acquired by spinning-disk confocal microscopy (Nikon), and the sperm number was quantified using Imaris 9.3 software through 3D reconstruction of the germline.

(B) Bar graphs showing that the warmer temperature treatment induces Mog in wild-isolated *C. elegans* strain ED3017. The number of sperm in ED3017 worms with 30 °C treatment was determined by the commonly used DAPI staining assay (Tang & Han, 2017). A total of 52.5% of worms with the 30 °C treatment displayed Mog, in contrast

to the absence of the Mog phenotype in worms cultured at a constant 20 °C (B). *P* value = 6.45×10^{-6}.

(C) Bar graphs showing the effect of warmer temperature on Mog induction in wild-isolated *C. elegans* EG4725. A total of 65.3% of worms with 30 °C treatment exhibited Mog phenotype, while no such phenotype was observed in worms cultured at constant 20 °C (C). *P* value = 1.63×10^{-5}.

The data are shown as the means ± SEMs. Each dot represents the percentage of germline arms showing Mog in each replicate in (B and C). Statistical analyses were performed by unpaired t test. Three biological independent replicates were conducted.

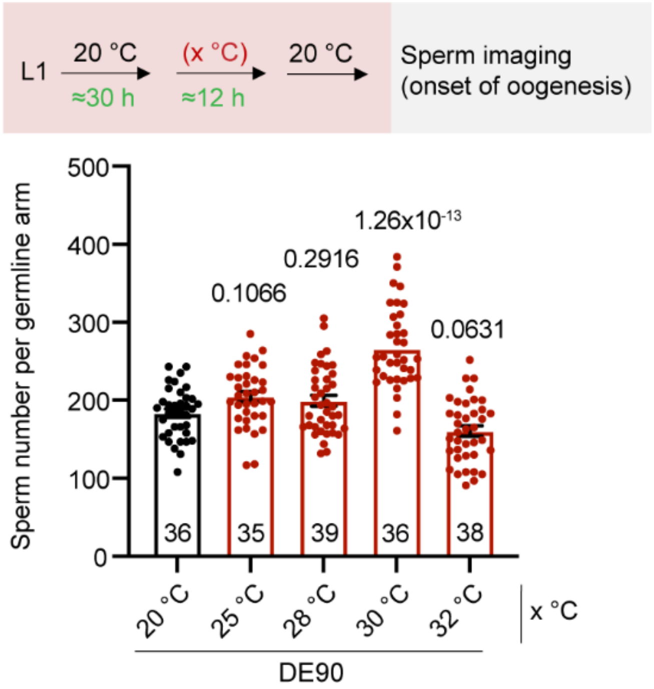

Appendix Figure S2

The warmer temperature (30 °C) exhibits a strong masculinizing effect on the germline. 30 hours post-hatching, DE90 worms were treated with the indicated temperature (x °C) for 12 hours, as the schematic diagram described. Each dot represents the sperm number in one germline arm. The data are shown as the means ± SEMs. Statistical analyses were performed by One-way ANOVA. Three biological independent replicates. *P* value = 0.1066, *P* value = 0.2916, *P* value = 1.26×10^{-13}, *P* value = 0.0631 (from left to right).

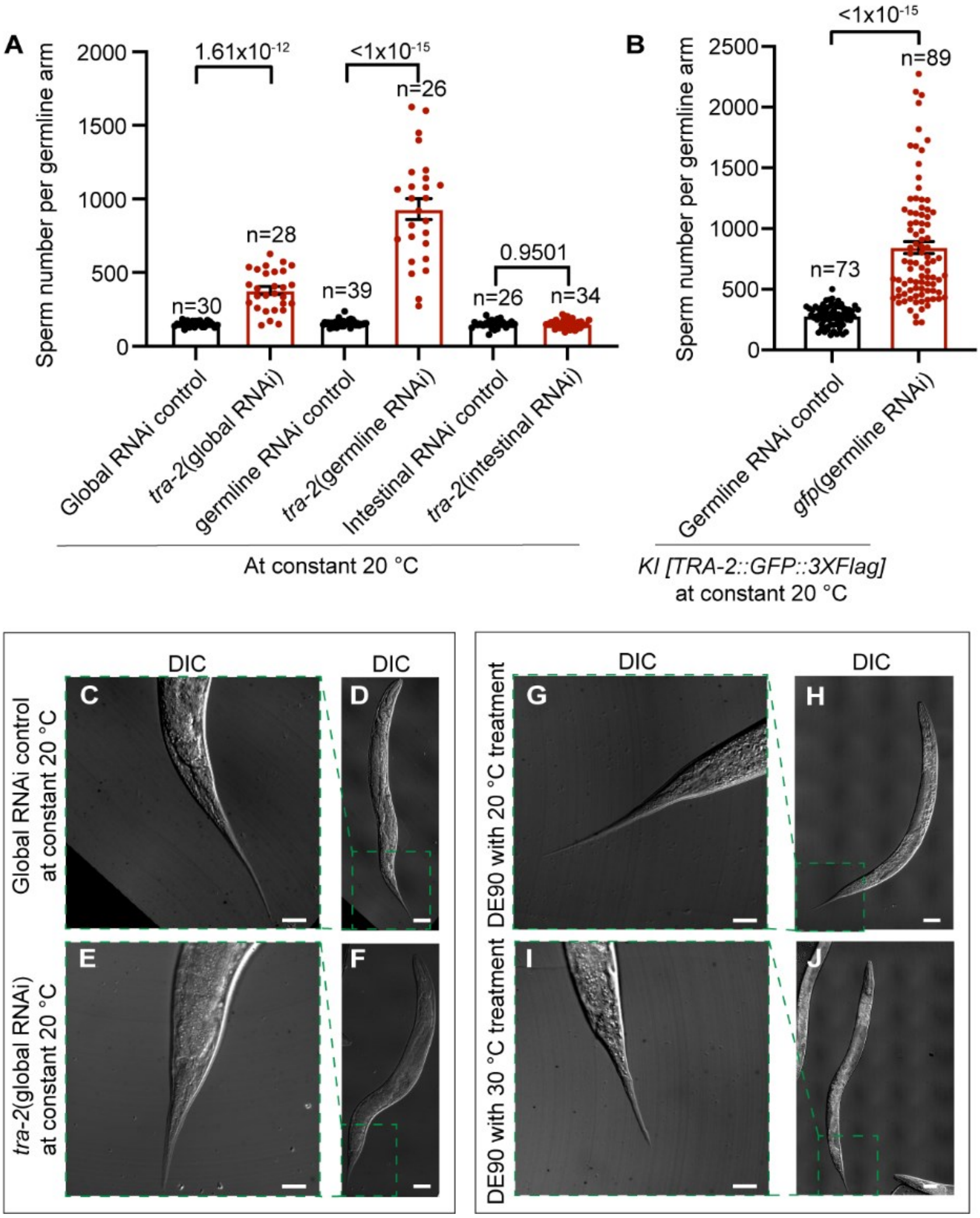

Appendix Figure S3

TRA-2 is expressed and functions in the germline to drive oocyte fate.

(A) Bar graph showing that TRA-2 functions in the germline to modulate germ cell fate, although it is expressed at an undetectable level in the germline (Fig. EV1D). The *Is [spe-11p::his-58::mCherry]* sperm marker strain, the VP303 strain and the DCL569 strain were cultured at a constant 20 °C and used for global RNAi, intestine-specific RNAi and germline-specific RNAi, respectively (Tang & Han, 2017; Zou *et al.*, 2019). *P* value = 1.61×10^{-12}, *P* value < 1×10^{-15}, *P* value = 0.9501 (from left to right).

(B) Bar graph showing that *KI [TRA-2::GFP::3×Flag]* worms with *gfp* knocking down specifically in the germline exhibited increased sperm production. *gfp(RNAi)* was performed in the *rrf-1(pk1417);KI [TRA-2::GFP::3×Flag];Is (spe-11p::his-58::mCherry)I]* strain to knock down TRA-2::GFP::3×Flag specifically in the germline. *rrf-1(lf)* enabled germline-specific RNAi (Sijen *et al*, 2001) and *Is (spe-11p::his58::mCherry)* were used to mark sperm. This data indicates that TRA-2::GFP::3×Flag is indeed expressed and functions in the germline, although the GFP signal is undetectable (Fig. EV1D). *P* value < 1×10^{-15}.

(C-F) Micrographs showing the tail morphology (C, E) and whole body (D, F) of *Is [spe-11p::his-58::mCherry]* worms with control RNAi or *tra-2* RNAi. The *tra-2*(global RNAi) induced masculinization of the germline (Appendix Fig. S3A) but not the soma, as evidenced by the typical hermaphroditic tail observed in both the control RNAi- and *tra-2* (RNAi)-treated animals (C and E). Magnified areas of tails of the corresponding worms are enclosed in green inset boxes. n = 60 worms.

(G-J) Representative images of the tail shape (G, I) and whole body (H, J) of DE90 worms with indicated treatment. The 30 °C treatment did not induce masculinization of the soma, as supported by the appearance of the typical hermaphroditic tail (I). The enlarged images of the green boxed areas show the tails of the corresponding worms. n = 60 worms.

All RNAi experiments were conducted by the microinjection methods. Scale bar: 50 μm in (D, F, H and J) and 20 μm in (C, E, G and I). Each dot represents the sperm number in one germline arm in (A and B). The data are shown as the means ± SEMs. Statistical analyses were performed by unpaired t test. All experiments were performed at least three times independently.

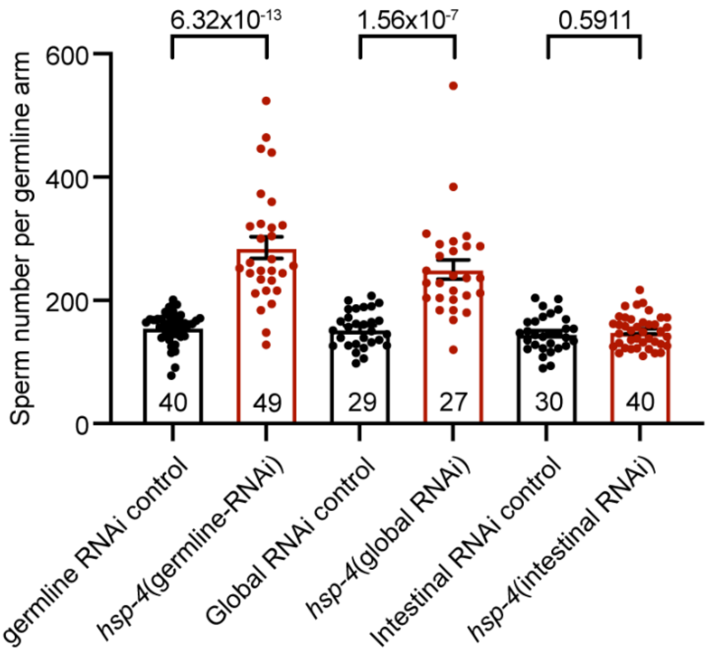

Appendix Figure S4

BiPs function in the germline to modulate germ cell fate.

Bar graph showing that HSP-4 functions in the germline to modulate germ cell fate. The *hsp-4(*RNAi) was performed by microinjection in the DCL569 worms carrying the *Is [sybSi36(spe-11p::his-58::mCherry)]* sperm marker, the *Is [sybSi36(spe-11p::his-58::mCherry)]* sperm marker strain and the VP303 strain grown at a constant 20 °C, and were indicated as germline-specific RNAi, global RNAi and intestine-specific RNAi, respectively. Germline-specific and global knockdown of *hsp-4* induced excess sperm production, while intestine-specific knockdown did not, indicating that *hsp-4* acts in the germline to modulate germline sex determination. Each dot represents the sperm number in one germline arm. The data are presented as the means ± SEMs. Statistical analyses were performed by unpaired t test. Three biological replicates were performed. *P* value = 6.32×10^{-13}, *P* value = 1.56×10^{-7}, *P* value = 0.5911 (from left to right).
