# Supplementary figures and images for: Identification of BiP as a temperature sensor mediating temperature-induced germline sex reversal in C. elegans (part 1 of 2)
Source: EMBO J. 2024 Aug 12;43(18):4020–48. doi: 10.1038/s44318-024-00197-z (PMC11405683; doi:10.1038/s44318-024-00197-z)

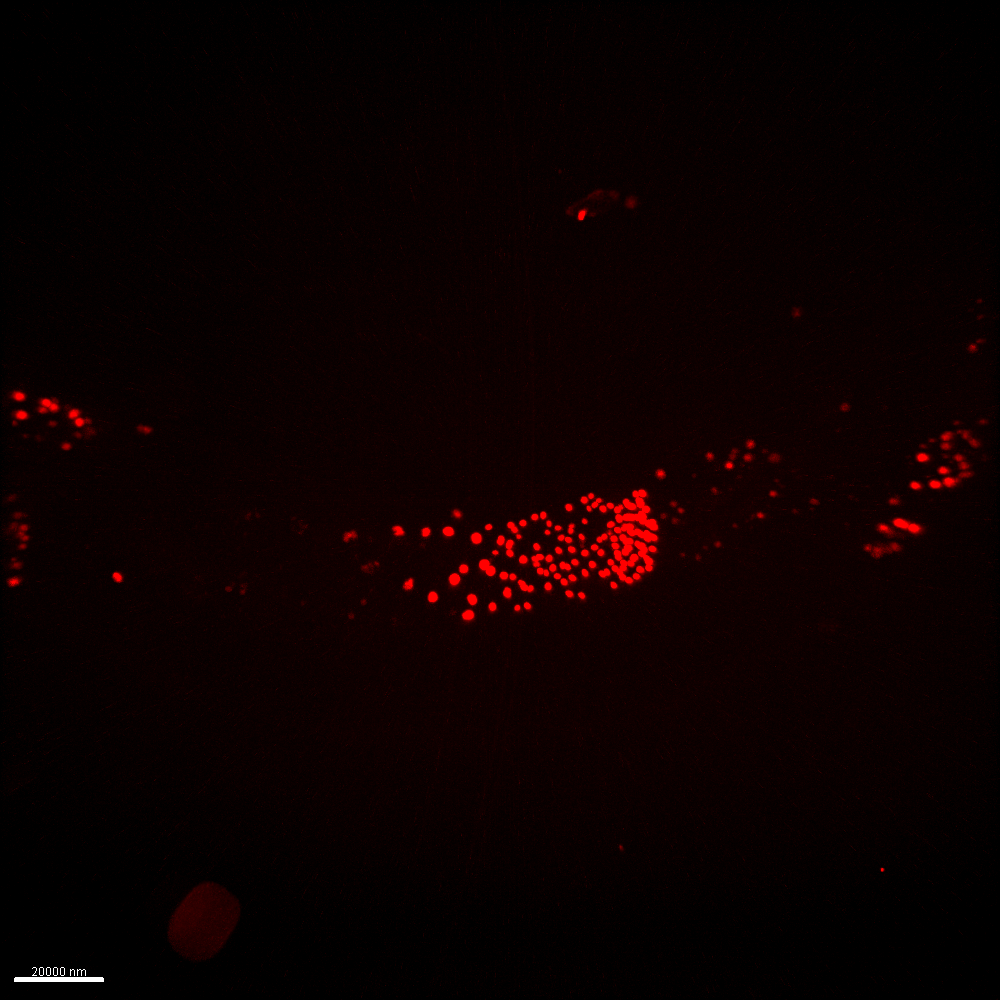

Supplement: Supplementary file 3 — Source data Fig. 1 [file 44318_2024_197_MOESM3_ESM.zip › SD figure 1/1C/1C.tif]

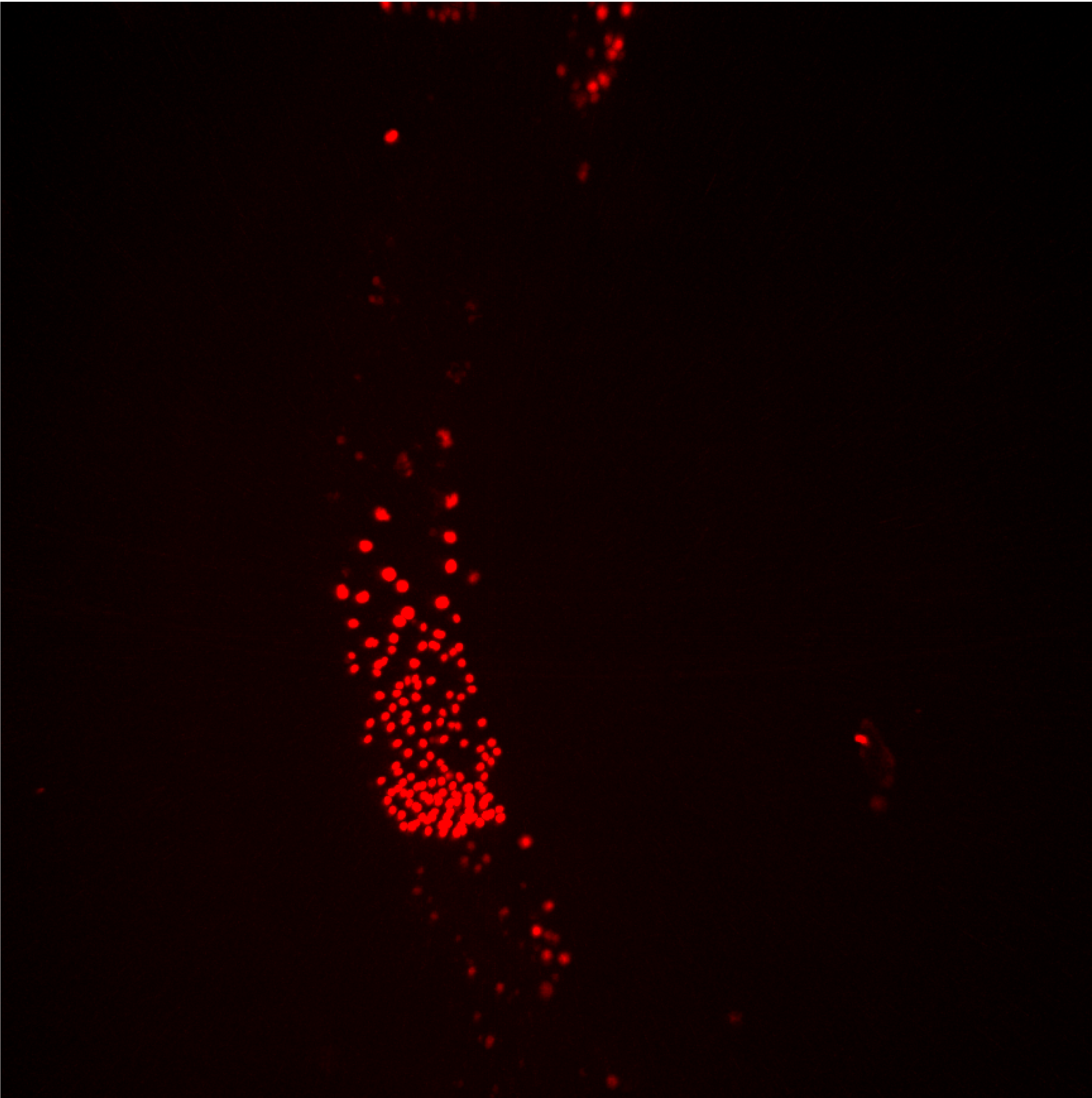

Supplement: Supplementary file 3 — Source data Fig. 1 [file 44318_2024_197_MOESM3_ESM.zip › SD figure 1/1C/crop/1C crop.tif]

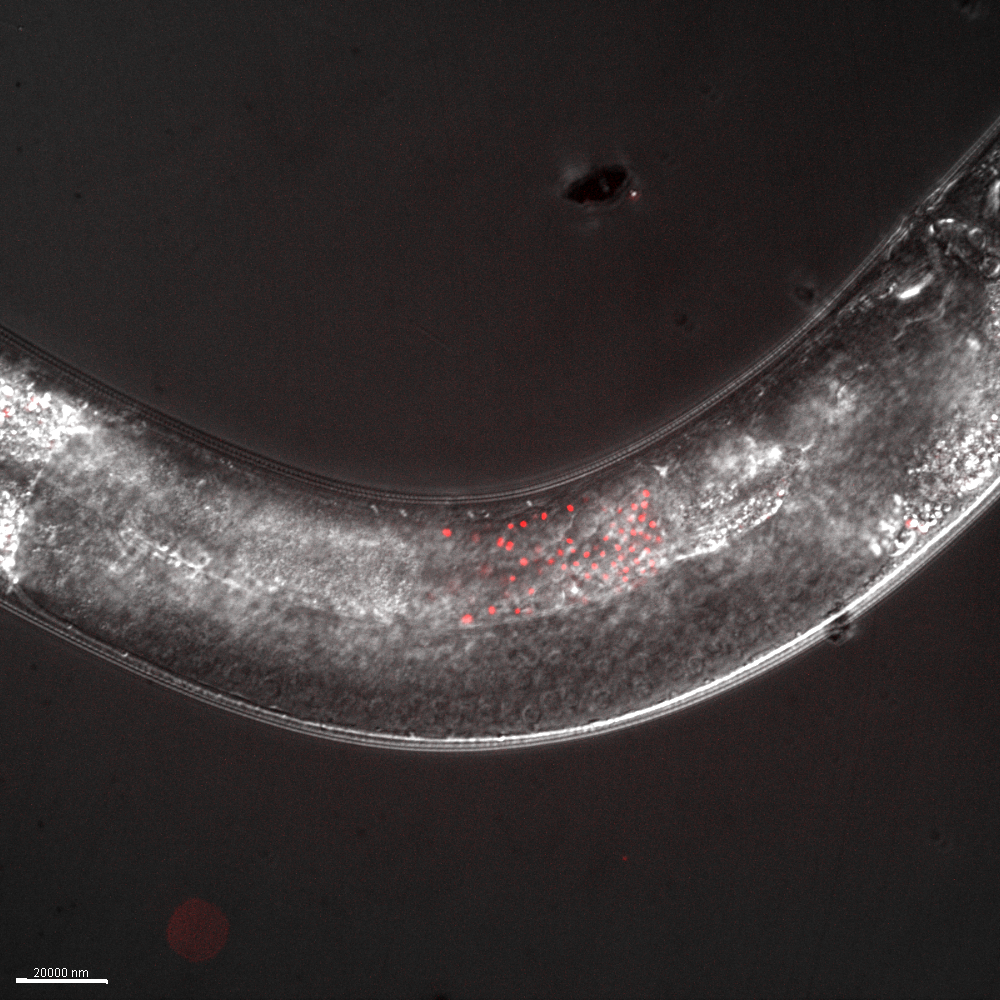

Supplement: Supplementary file 3 — Source data Fig. 1 [file 44318_2024_197_MOESM3_ESM.zip › SD figure 1/1D/1D.tif]

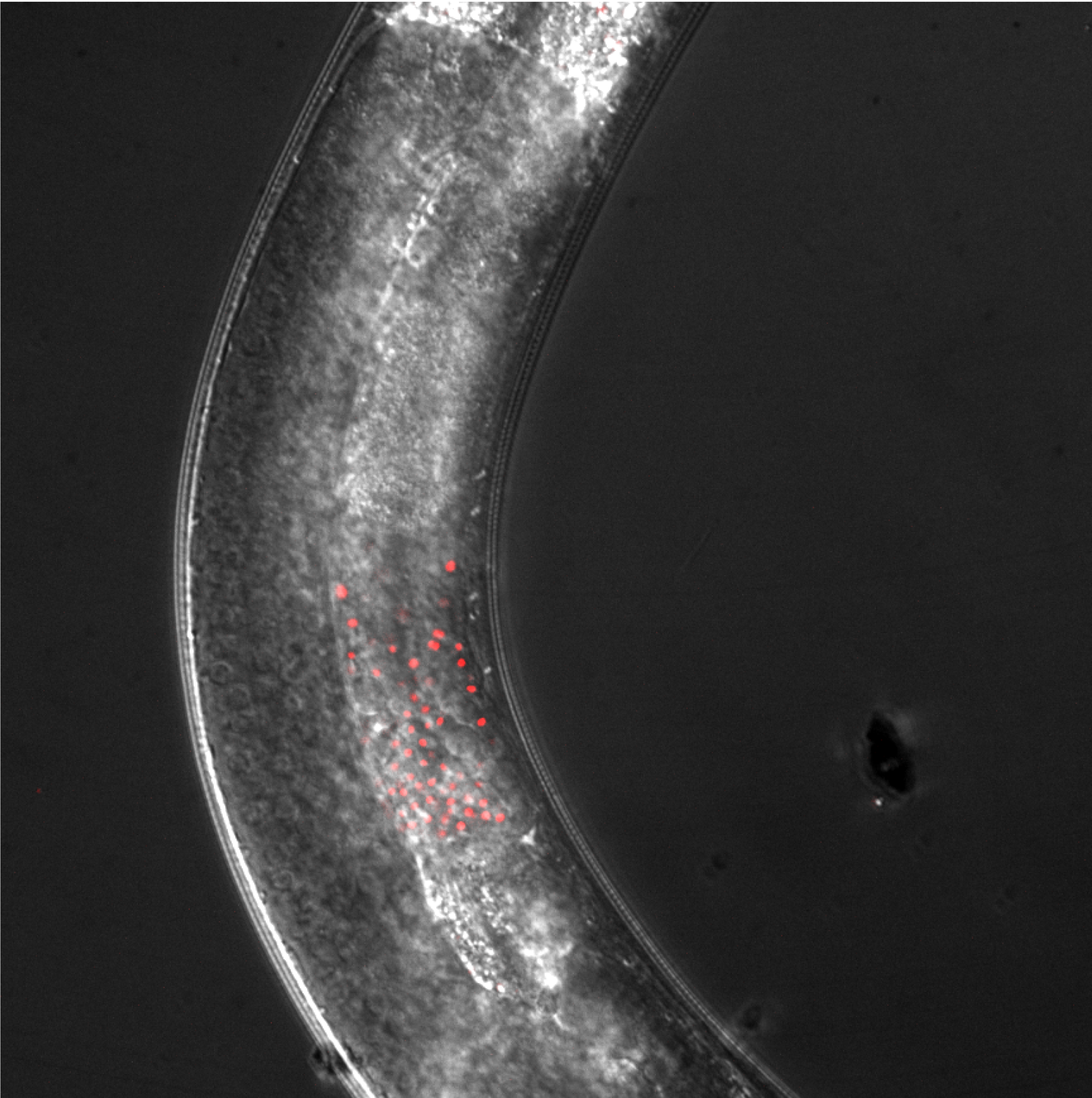

Supplement: Supplementary file 3 — Source data Fig. 1 [file 44318_2024_197_MOESM3_ESM.zip › SD figure 1/1D/crop/1D crop.tif]

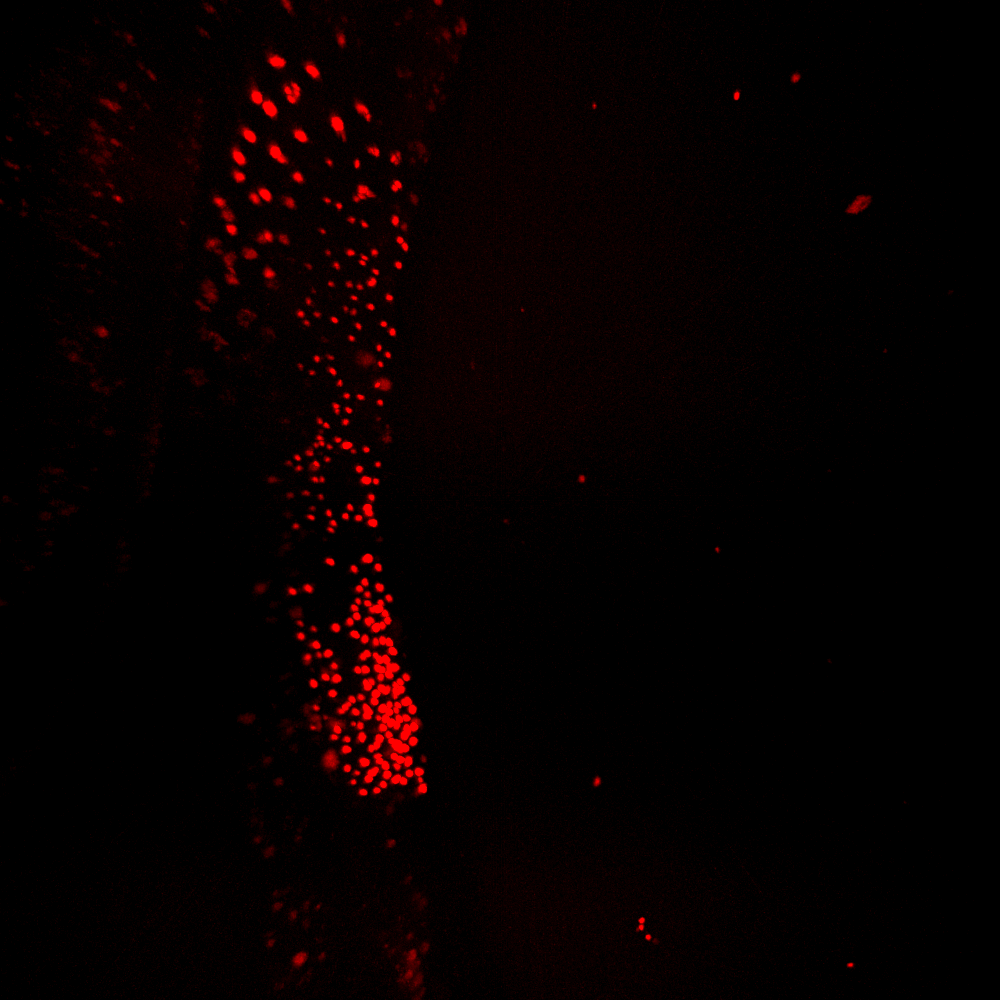

Supplement: Supplementary file 3 — Source data Fig. 1 [file 44318_2024_197_MOESM3_ESM.zip › SD figure 1/1E/1E.tif]

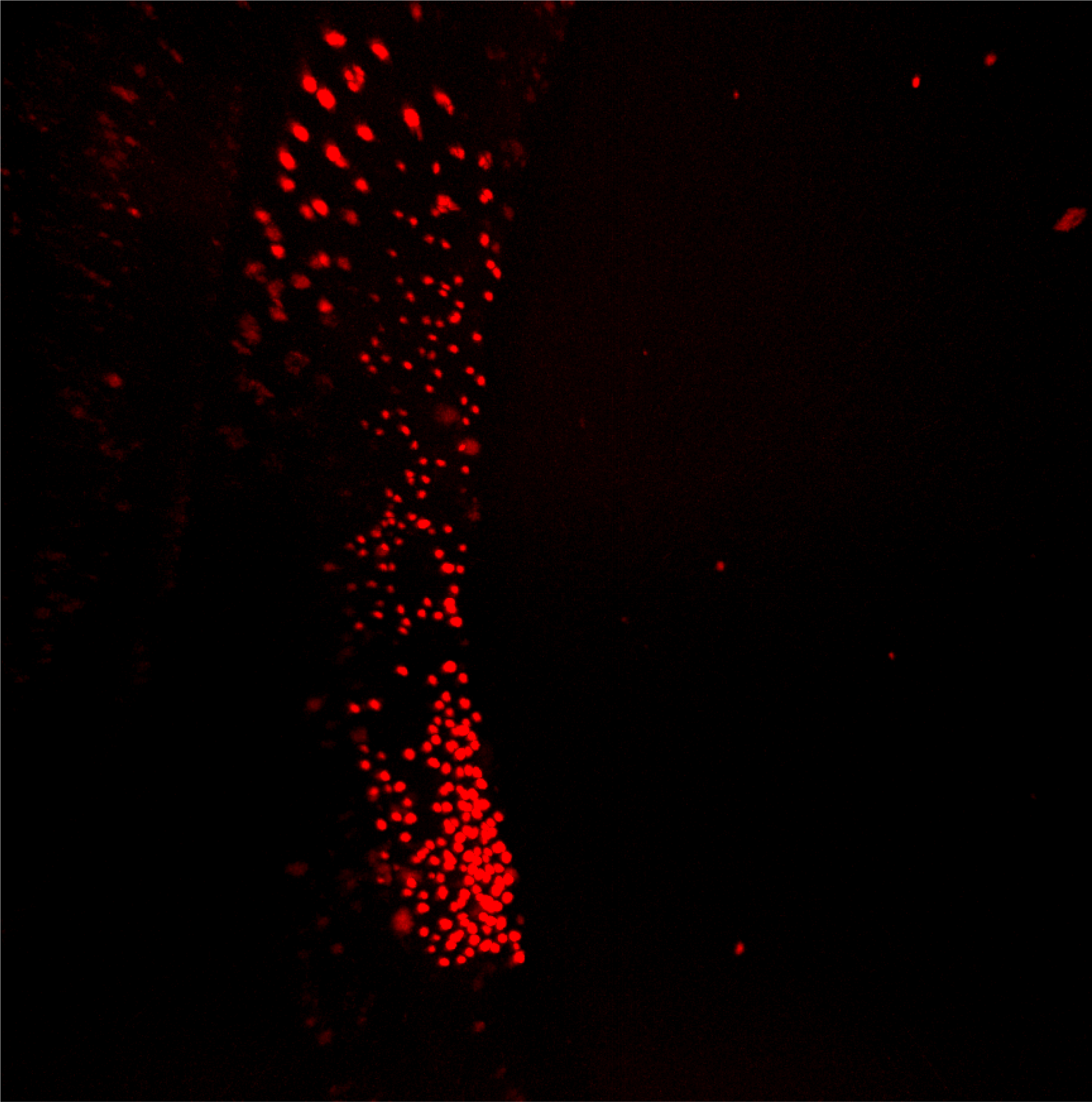

Supplement: Supplementary file 3 — Source data Fig. 1 [file 44318_2024_197_MOESM3_ESM.zip › SD figure 1/1E/crop/1E crop.tif]

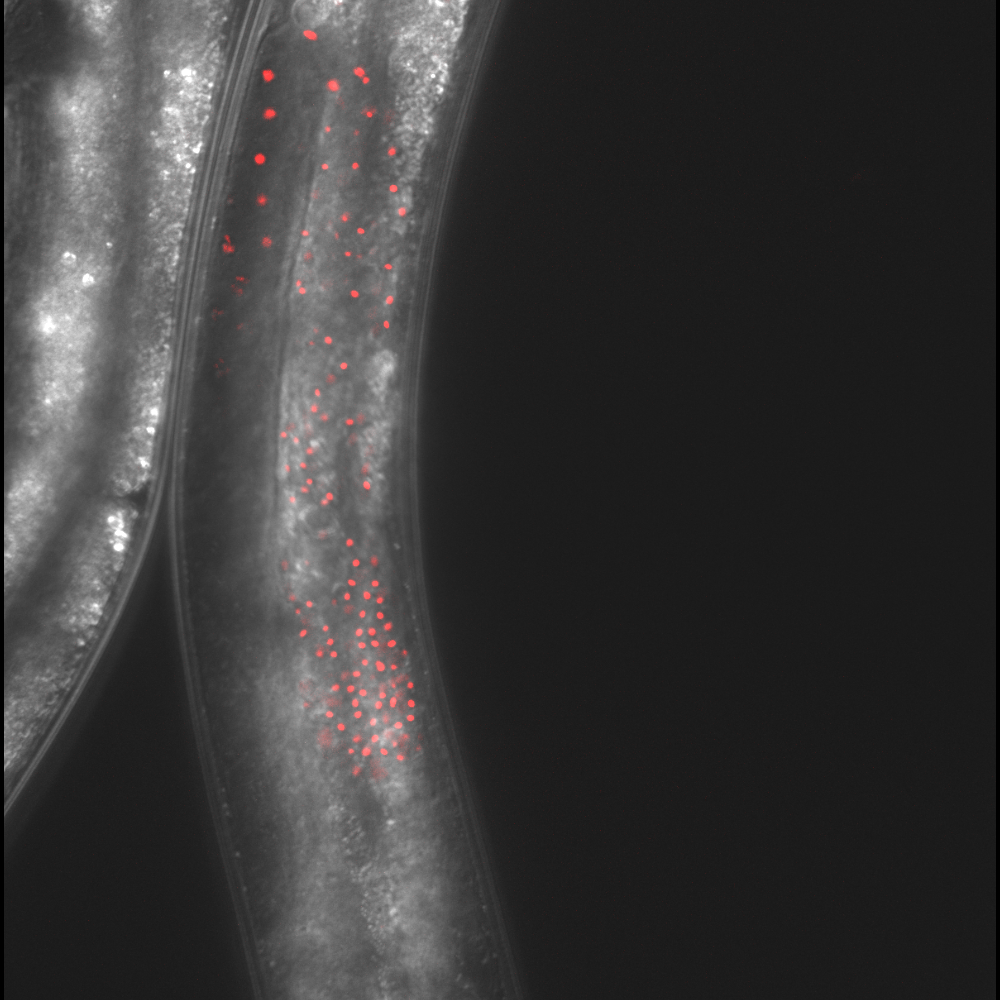

Supplement: Supplementary file 3 — Source data Fig. 1 [file 44318_2024_197_MOESM3_ESM.zip › SD figure 1/1F/1F.tif]

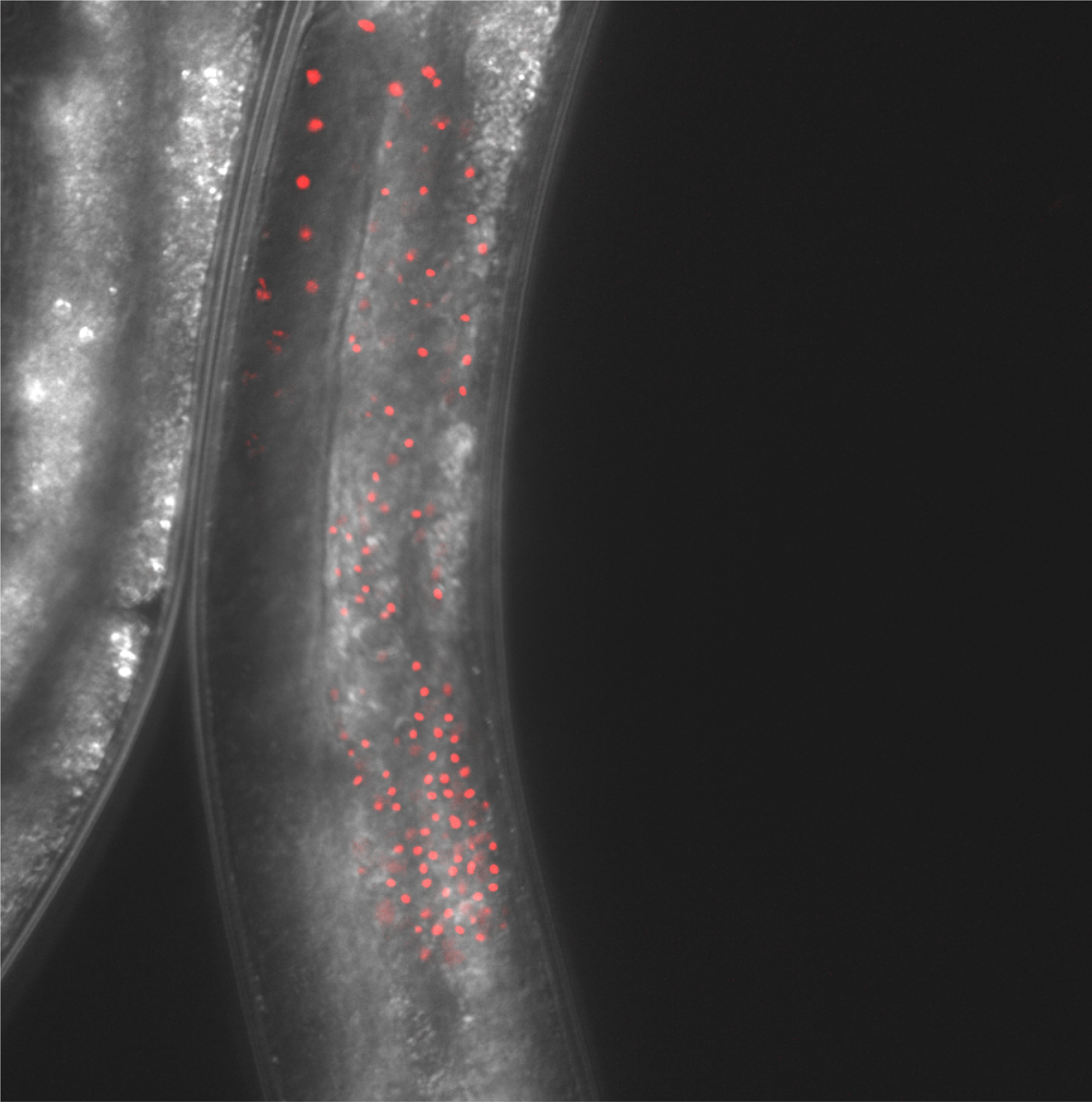

Supplement: Supplementary file 3 — Source data Fig. 1 [file 44318_2024_197_MOESM3_ESM.zip › SD figure 1/1F/crop/1F crop.tif]

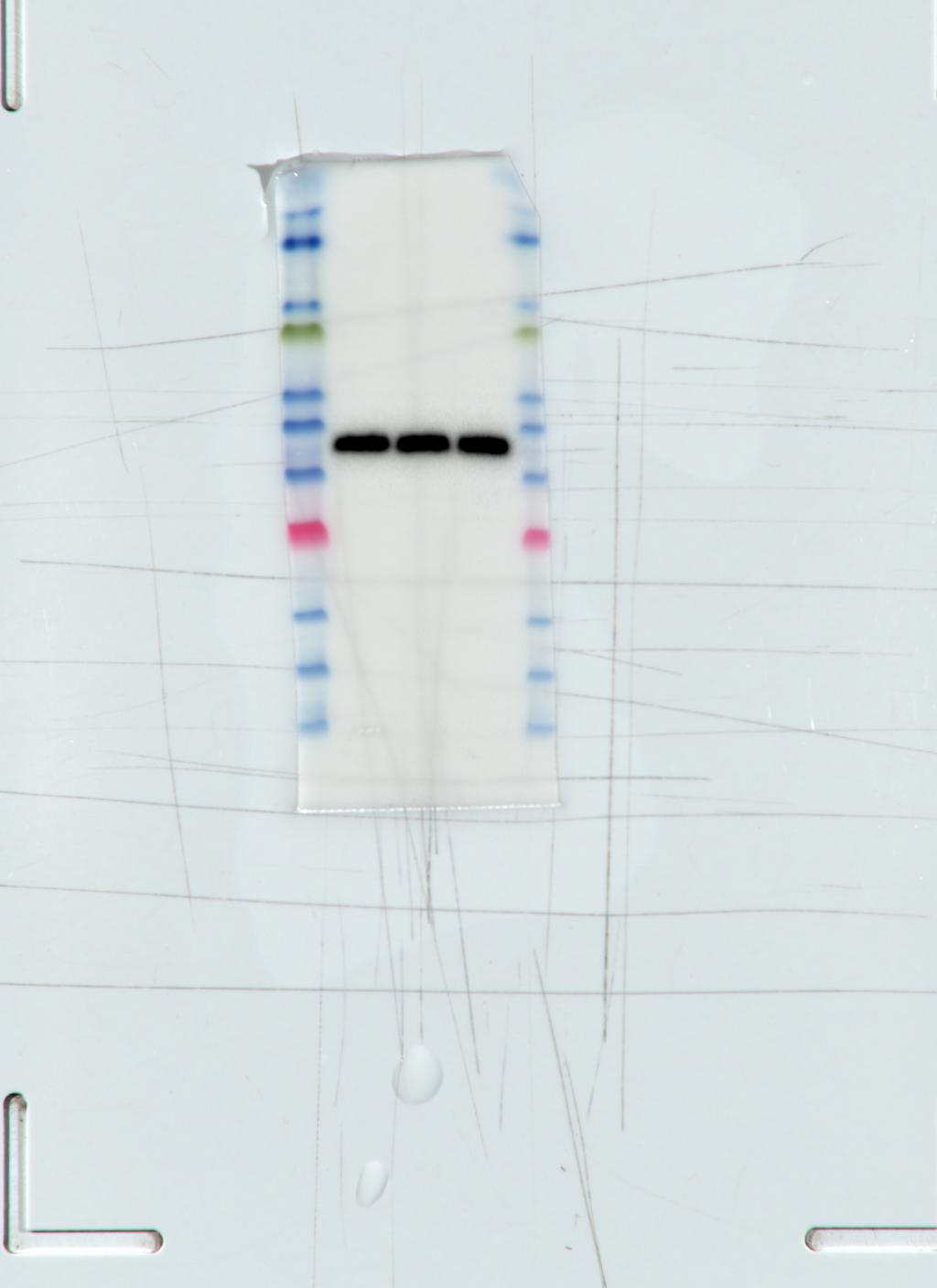

Supplement: Supplementary file 4 — Source data Fig. 2 [file 44318_2024_197_MOESM4_ESM.zip › SD figure 2/2C/2C replicate-1/beta-actin/2022.09.22_22.55.27_Ch+Marker.jpg]

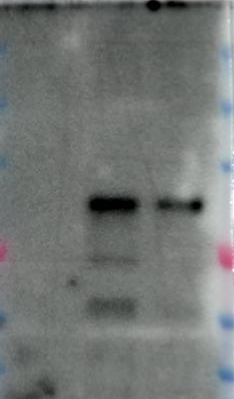

Supplement: Supplementary file 4 — Source data Fig. 2 [file 44318_2024_197_MOESM4_ESM.zip › SD figure 2/2C/2C replicate-1/TRA-2/TRA-2.tif]

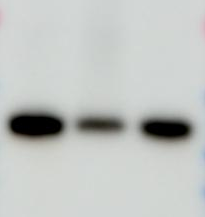

Supplement: Supplementary file 4 — Source data Fig. 2 [file 44318_2024_197_MOESM4_ESM.zip › SD figure 2/2C/2C replicate-2/beta-actin/beta-actin.tif]

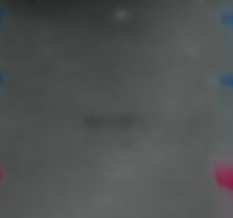

Supplement: Supplementary file 4 — Source data Fig. 2 [file 44318_2024_197_MOESM4_ESM.zip › SD figure 2/2C/2C replicate-2/TRA-2/TRA-2.tif]

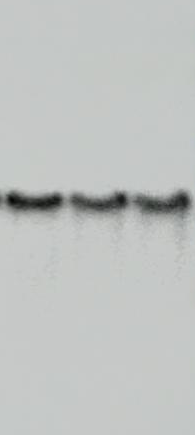

Supplement: Supplementary file 4 — Source data Fig. 2 [file 44318_2024_197_MOESM4_ESM.zip › SD figure 2/2C/2C replicate-3/beta-actin/beta-actin.tif]

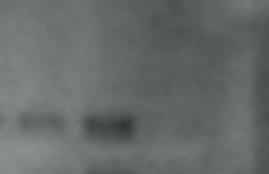

Supplement: Supplementary file 4 — Source data Fig. 2 [file 44318_2024_197_MOESM4_ESM.zip › SD figure 2/2C/2C replicate-3/TRA-2/TRA-2.tif]

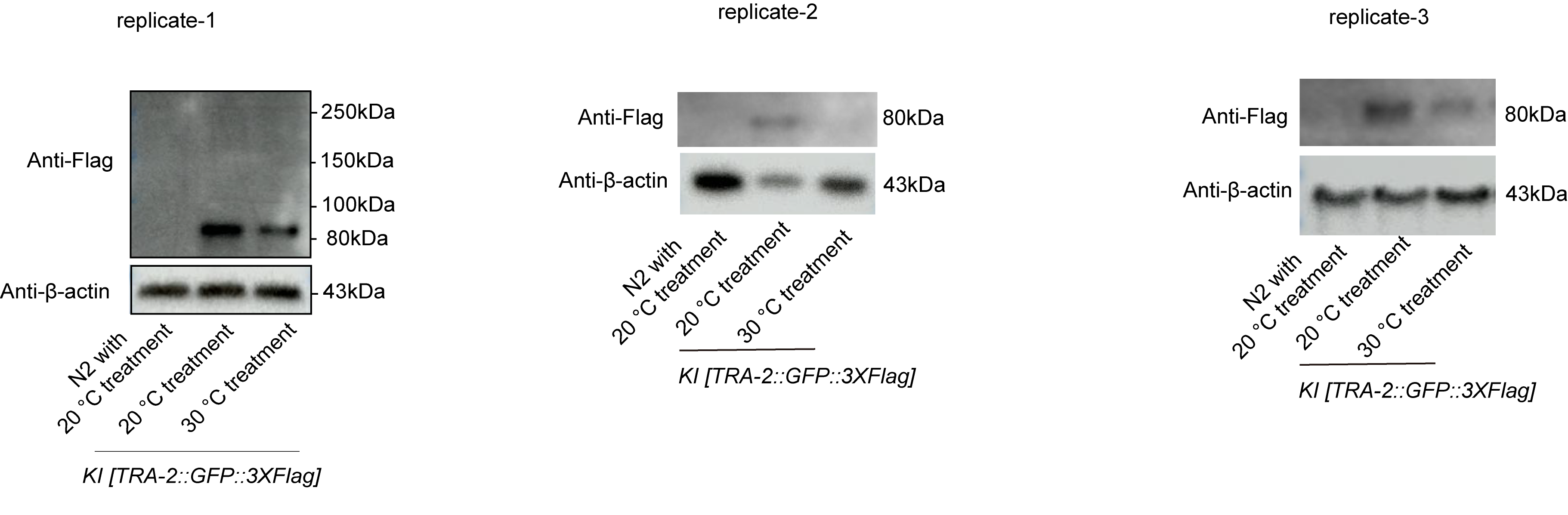

Supplement: Supplementary file 4 — Source data Fig. 2 [file 44318_2024_197_MOESM4_ESM.zip › SD figure 2/2C/2C.tif]

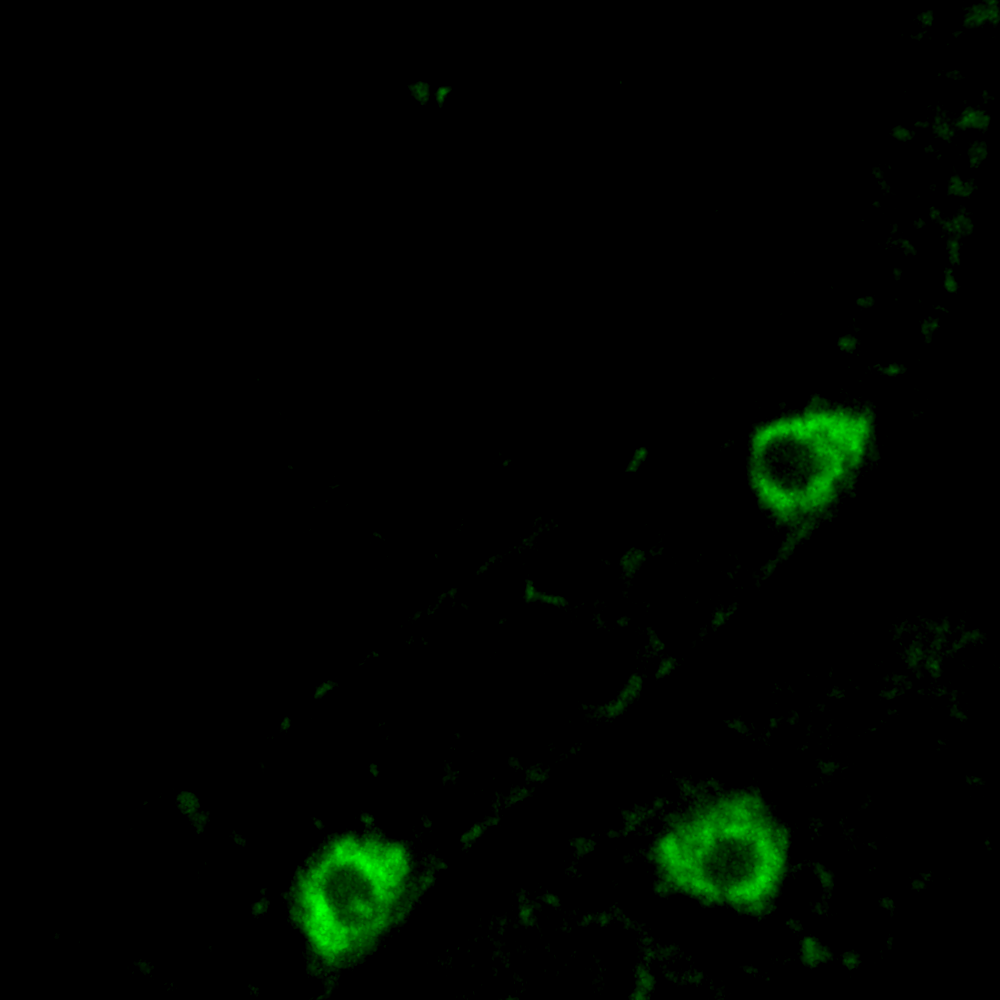

Supplement: Supplementary file 4 — Source data Fig. 2 [file 44318_2024_197_MOESM4_ESM.zip › SD figure 2/2E/2E.tif]

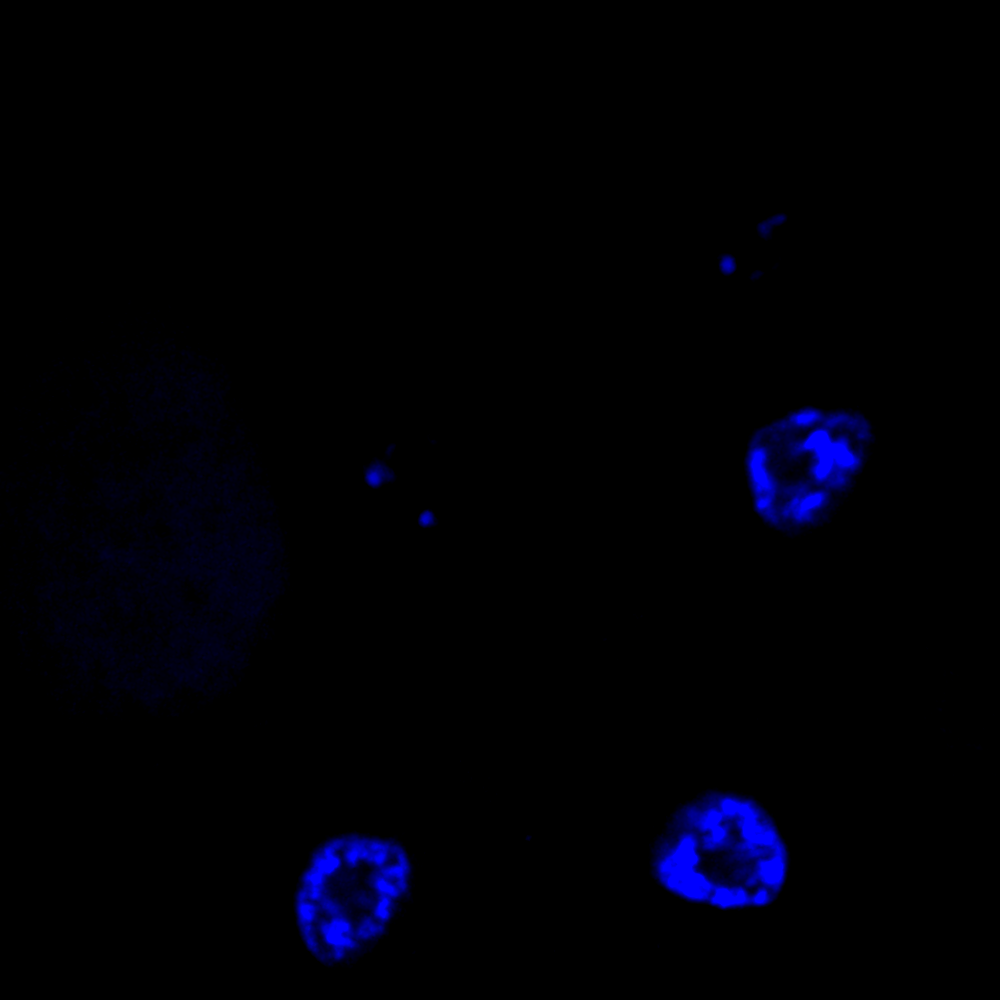

Supplement: Supplementary file 4 — Source data Fig. 2 [file 44318_2024_197_MOESM4_ESM.zip › SD figure 2/2F/2F.tif]

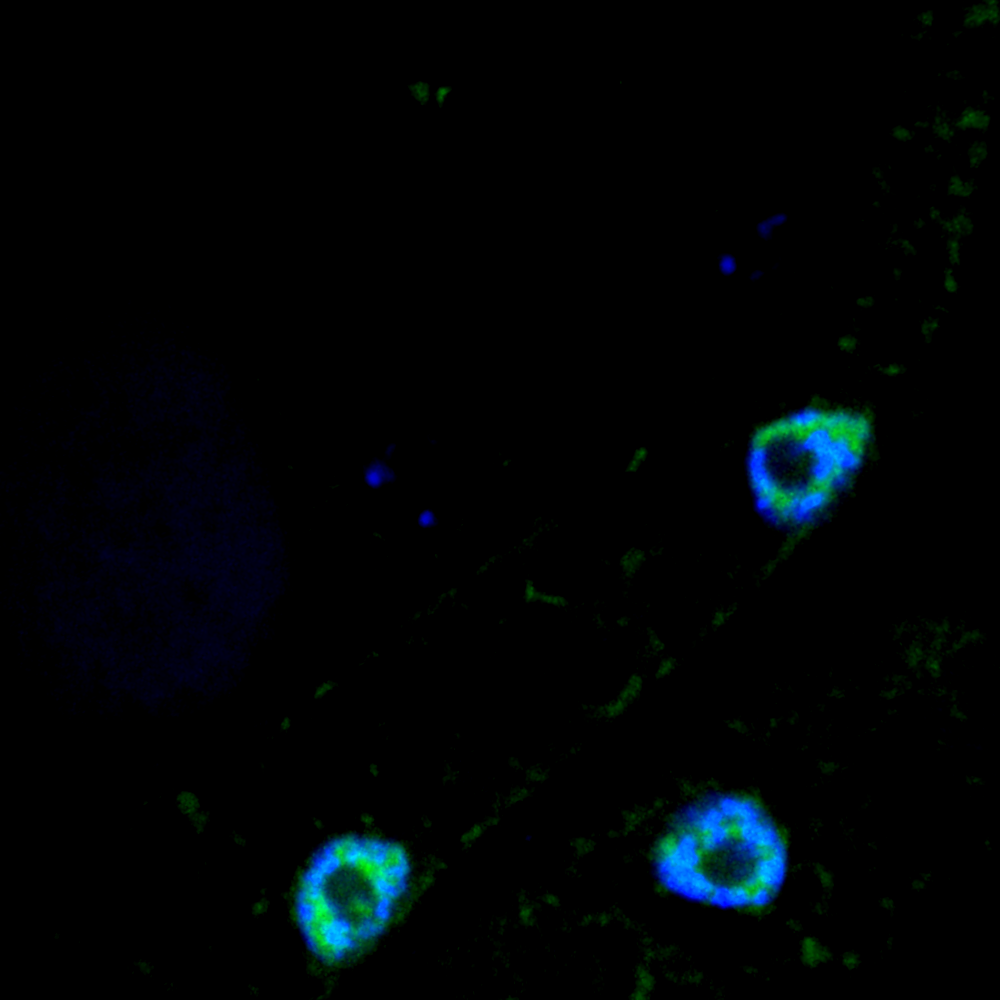

Supplement: Supplementary file 4 — Source data Fig. 2 [file 44318_2024_197_MOESM4_ESM.zip › SD figure 2/2G/2G.tif]

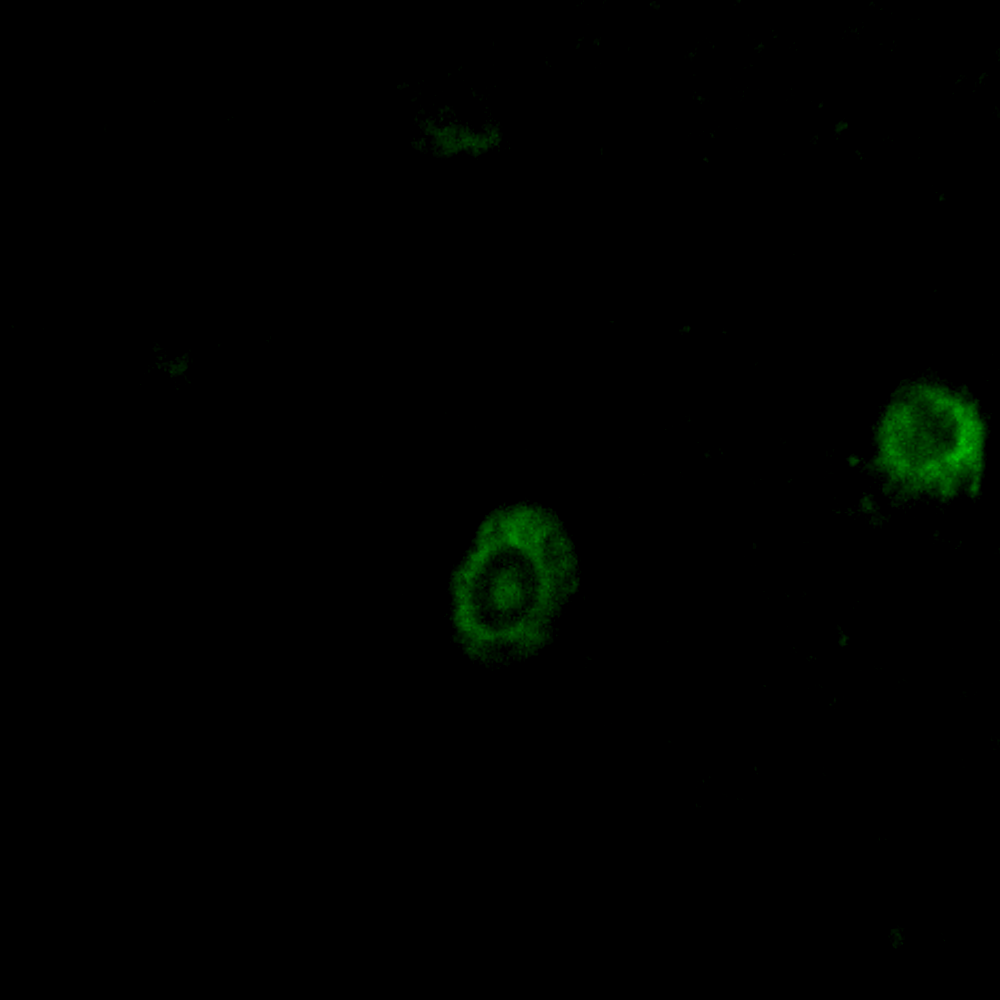

Supplement: Supplementary file 4 — Source data Fig. 2 [file 44318_2024_197_MOESM4_ESM.zip › SD figure 2/2H/2H.tif]

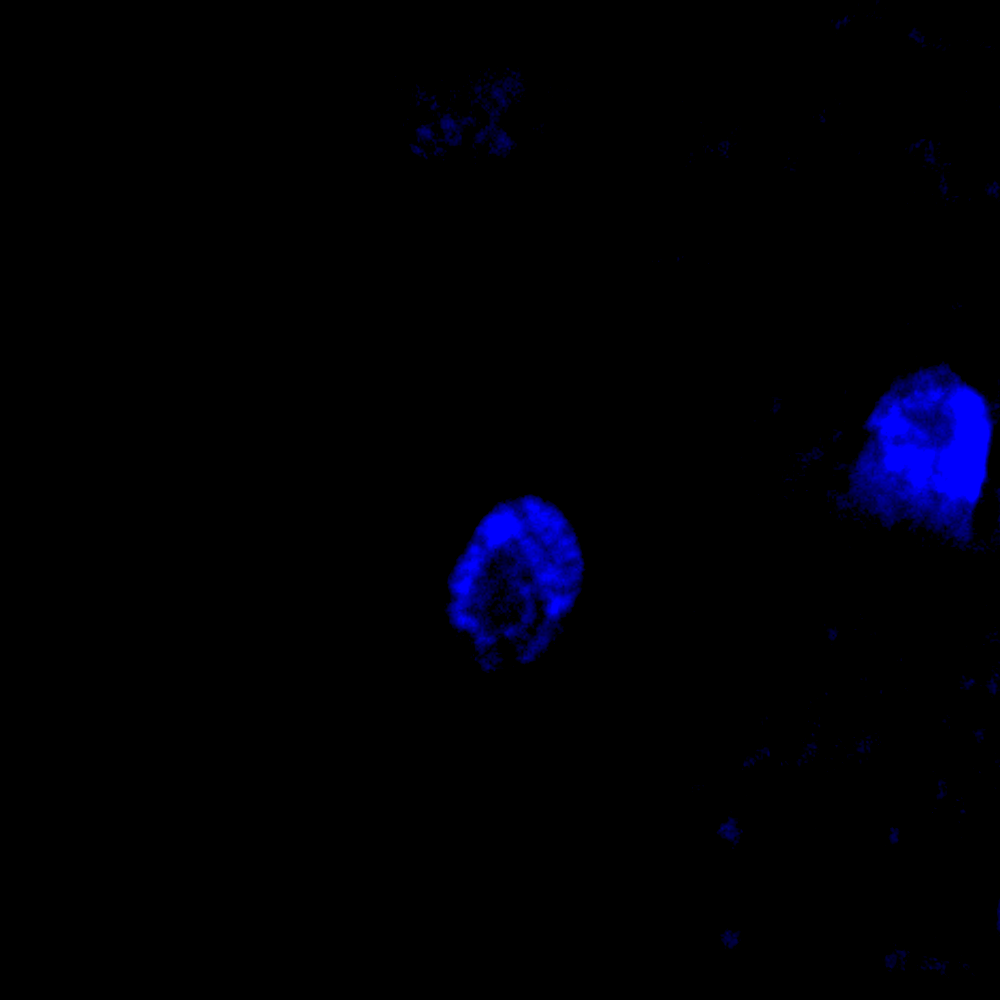

Supplement: Supplementary file 4 — Source data Fig. 2 [file 44318_2024_197_MOESM4_ESM.zip › SD figure 2/2I/2I.tif]

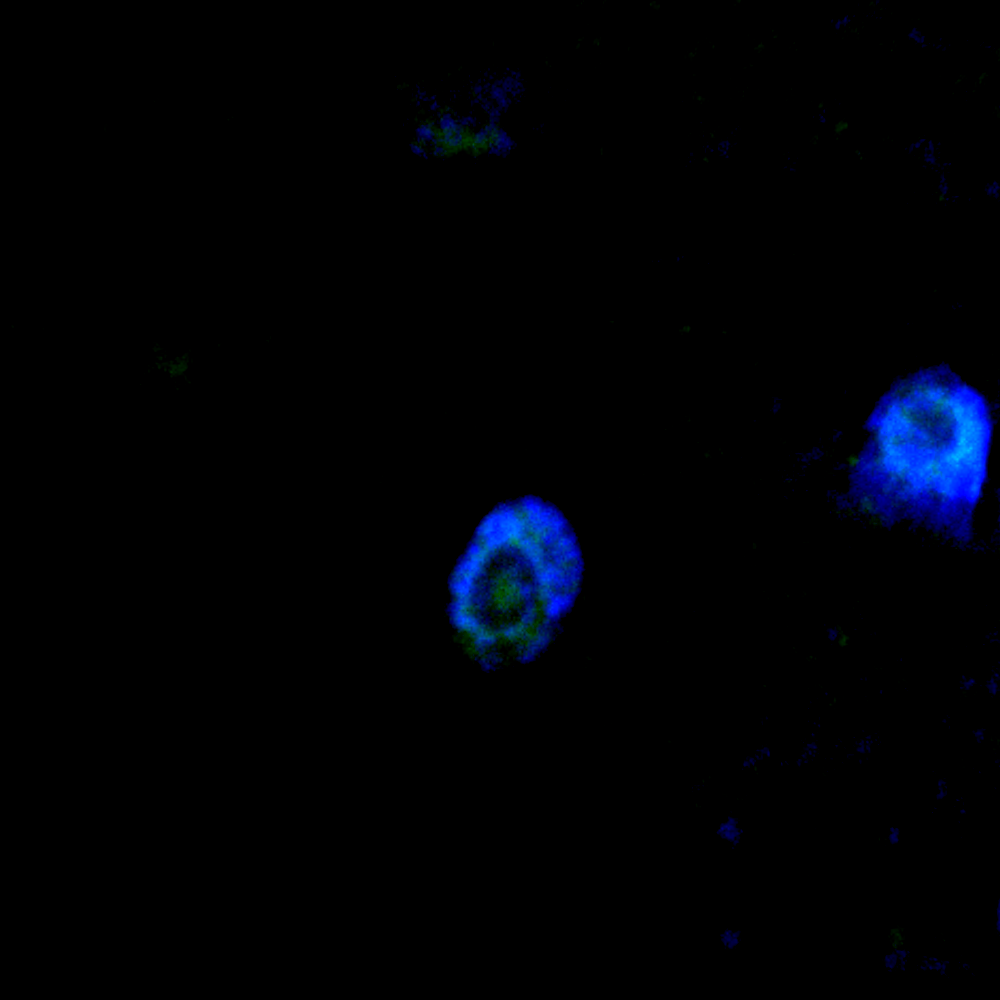

Supplement: Supplementary file 4 — Source data Fig. 2 [file 44318_2024_197_MOESM4_ESM.zip › SD figure 2/2J/2J.tif]

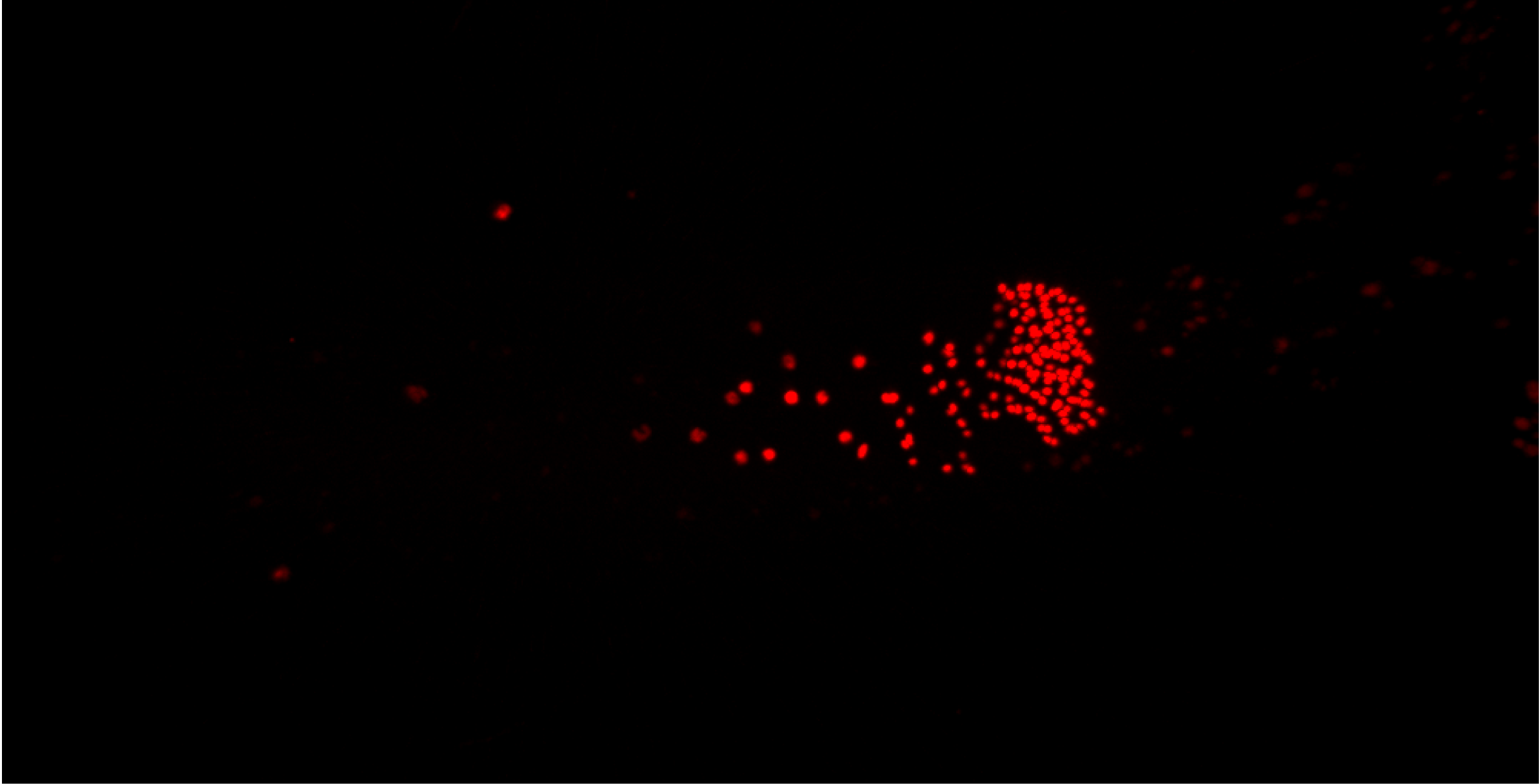

Supplement: Supplementary file 5 — Source data Fig. 3 [file 44318_2024_197_MOESM5_ESM.zip › SD figure 3/3A/3A.tif]

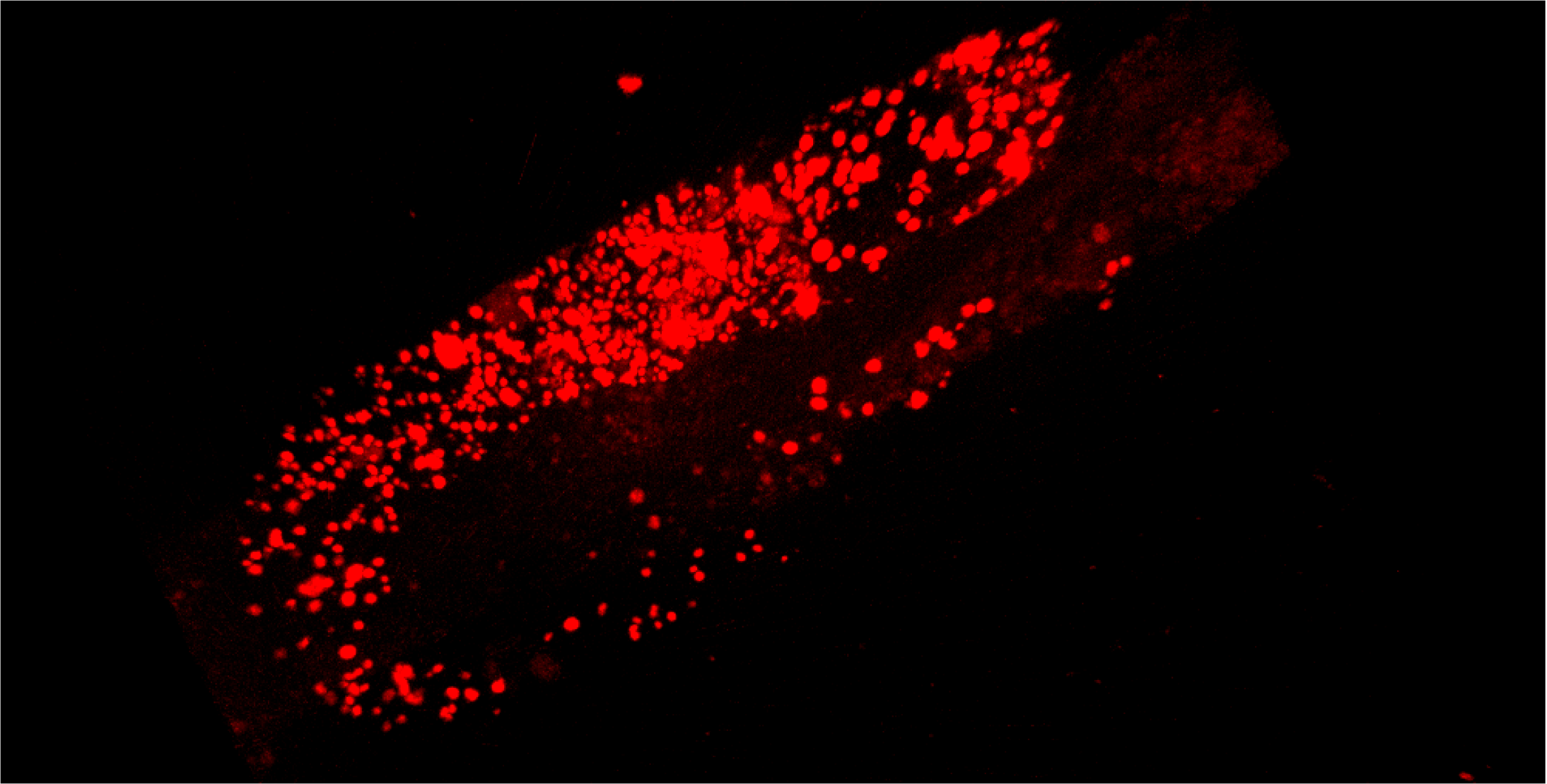

Supplement: Supplementary file 5 — Source data Fig. 3 [file 44318_2024_197_MOESM5_ESM.zip › SD figure 3/3B/3B.tif]

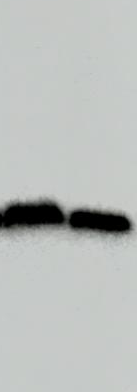

Supplement: Supplementary file 5 — Source data Fig. 3 [file 44318_2024_197_MOESM5_ESM.zip › SD figure 3/3F/3F replicate-1/beta-actin/beta-actin.tif]

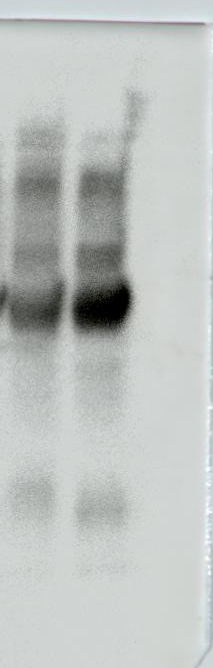

Supplement: Supplementary file 5 — Source data Fig. 3 [file 44318_2024_197_MOESM5_ESM.zip › SD figure 3/3F/3F replicate-1/IP-free BiP/IP-free BiP.tif]

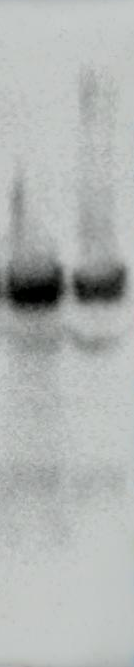

Supplement: Supplementary file 5 — Source data Fig. 3 [file 44318_2024_197_MOESM5_ESM.zip › SD figure 3/3F/3F replicate-1/lysate-total BiP/lysate-total BiP.tif]

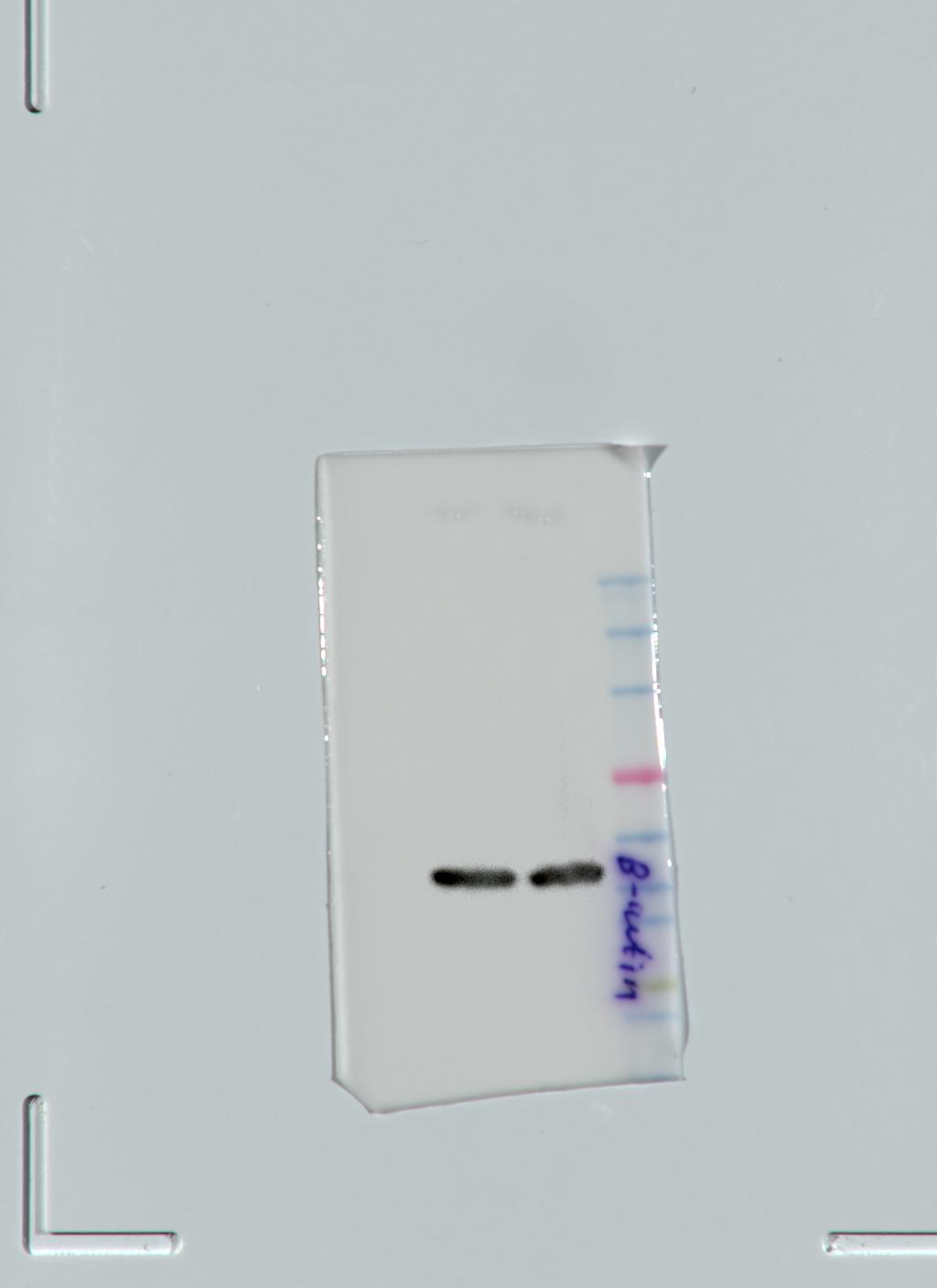

Supplement: Supplementary file 5 — Source data Fig. 3 [file 44318_2024_197_MOESM5_ESM.zip › SD figure 3/3F/3F replicate-2/beta-actin/beta-actin.tif]

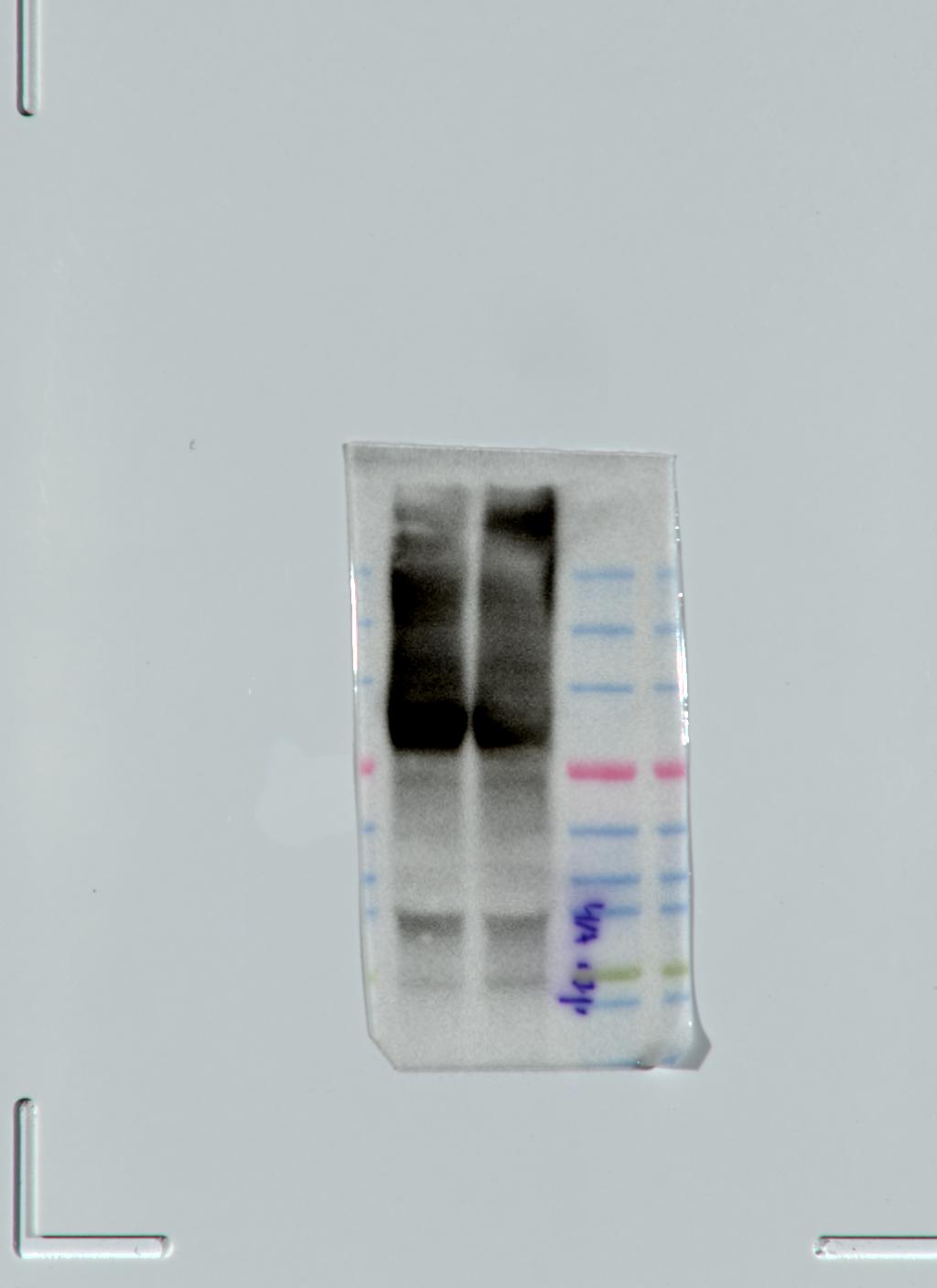

Supplement: Supplementary file 5 — Source data Fig. 3 [file 44318_2024_197_MOESM5_ESM.zip › SD figure 3/3F/3F replicate-2/IP-free BiP/IP-free BiP.tif]

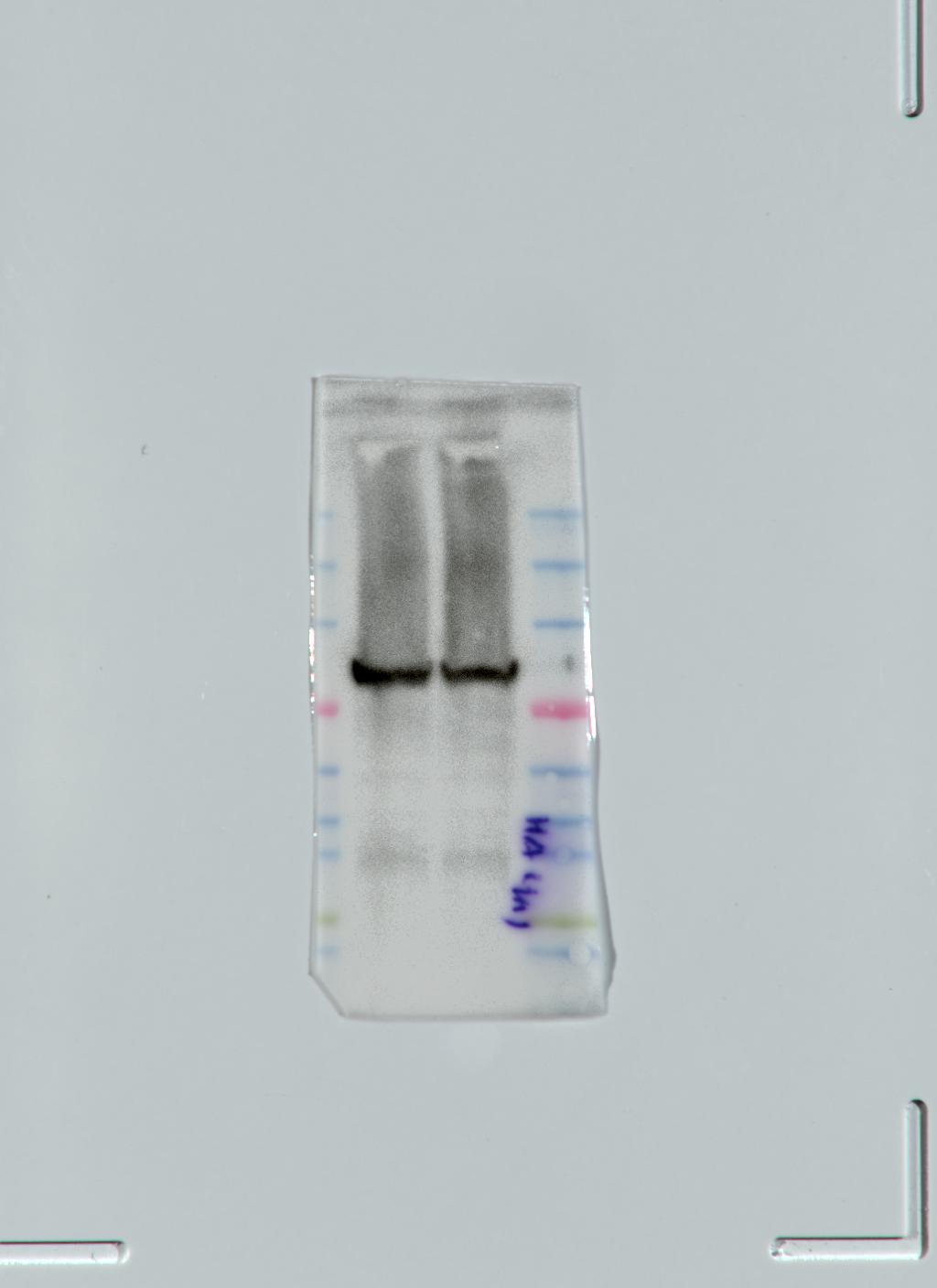

Supplement: Supplementary file 5 — Source data Fig. 3 [file 44318_2024_197_MOESM5_ESM.zip › SD figure 3/3F/3F replicate-2/lysate-total BiP/total BiP.tif]

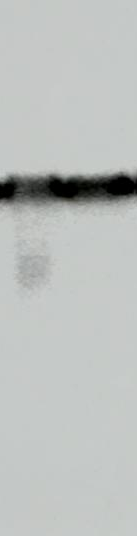

Supplement: Supplementary file 5 — Source data Fig. 3 [file 44318_2024_197_MOESM5_ESM.zip › SD figure 3/3F/3F replicate-3/beta-actin/beta-actin.tif]

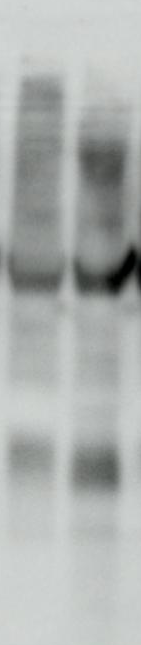

Supplement: Supplementary file 5 — Source data Fig. 3 [file 44318_2024_197_MOESM5_ESM.zip › SD figure 3/3F/3F replicate-3/IP-free BiP/IP-free BiP.tif]

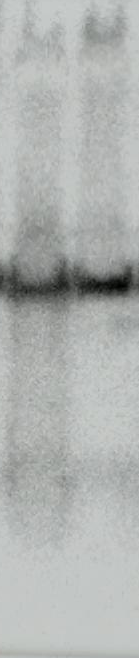

Supplement: Supplementary file 5 — Source data Fig. 3 [file 44318_2024_197_MOESM5_ESM.zip › SD figure 3/3F/3F replicate-3/lysate-total BiP/lysate-total BiP.tif]

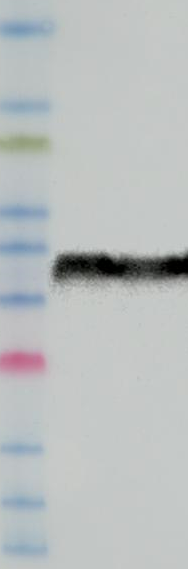

Supplement: Supplementary file 5 — Source data Fig. 3 [file 44318_2024_197_MOESM5_ESM.zip › SD figure 3/3F/3F replicate-4/beta-actin/beta-actin.tif]

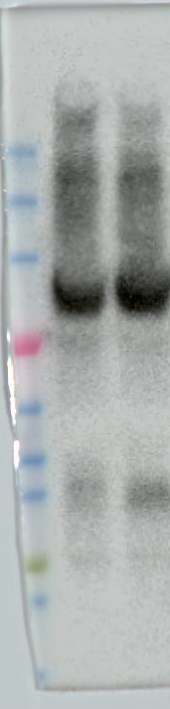

Supplement: Supplementary file 5 — Source data Fig. 3 [file 44318_2024_197_MOESM5_ESM.zip › SD figure 3/3F/3F replicate-4/IP-free BiP/IP-free BiP.tif]

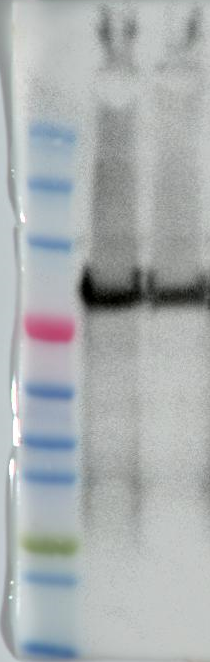

Supplement: Supplementary file 5 — Source data Fig. 3 [file 44318_2024_197_MOESM5_ESM.zip › SD figure 3/3F/3F replicate-4/lysate-total BiP/lysate-total BiP.tif]

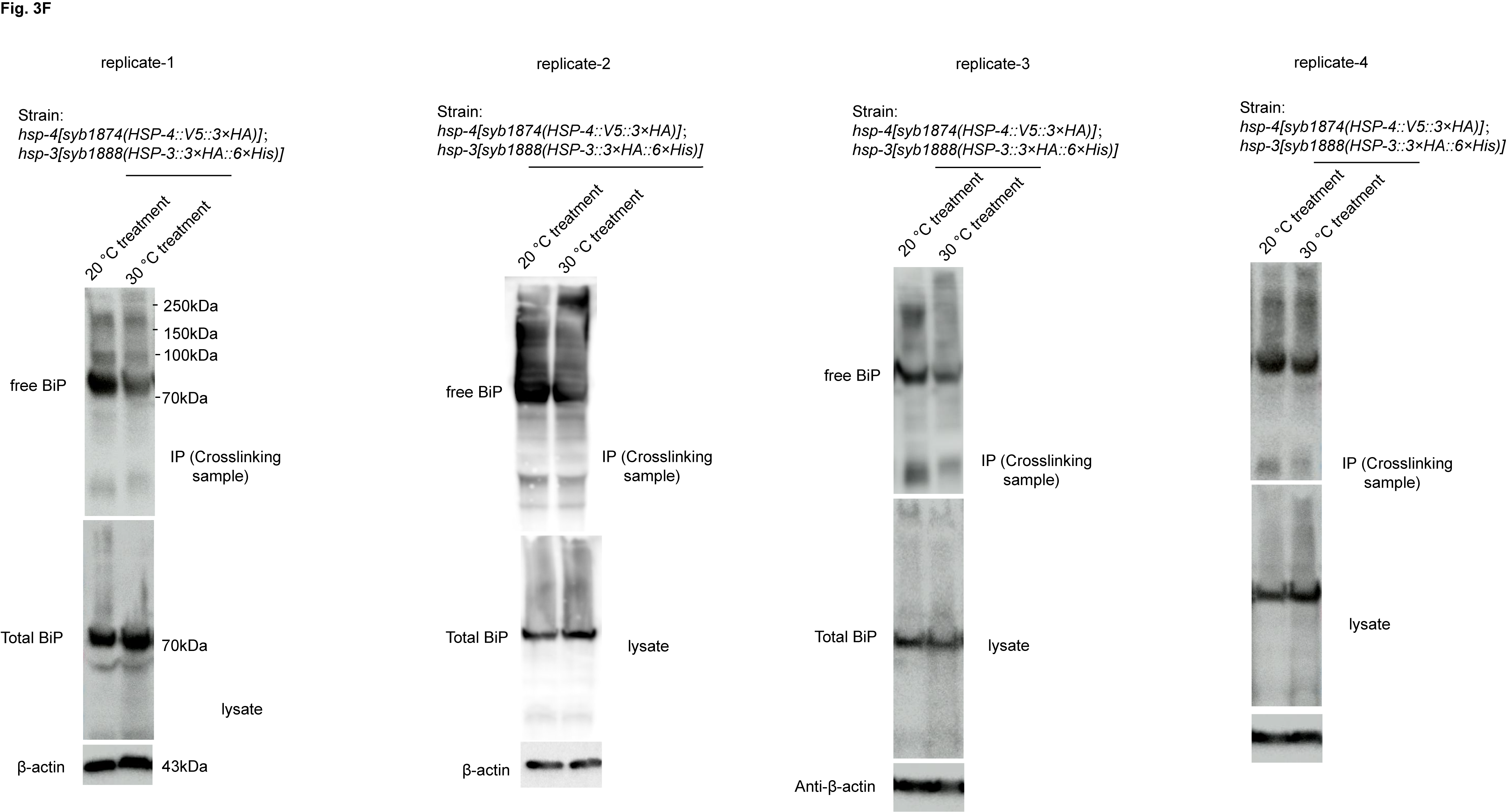

Supplement: Supplementary file 5 — Source data Fig. 3 [file 44318_2024_197_MOESM5_ESM.zip › SD figure 3/3F/3F.tif]

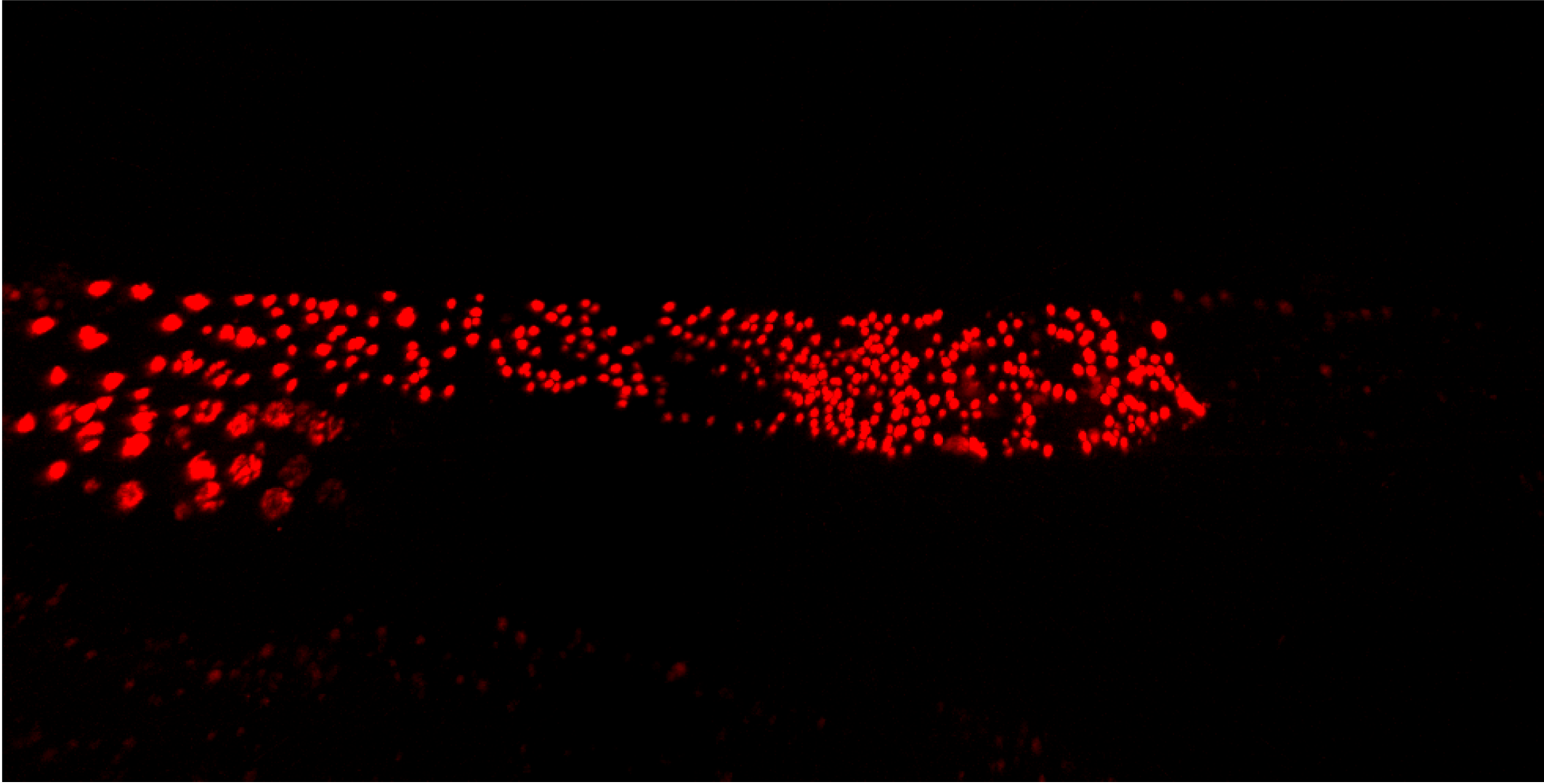

Supplement: Supplementary file 5 — Source data Fig. 3 [file 44318_2024_197_MOESM5_ESM.zip › SD figure 3/3H/3H.tif]

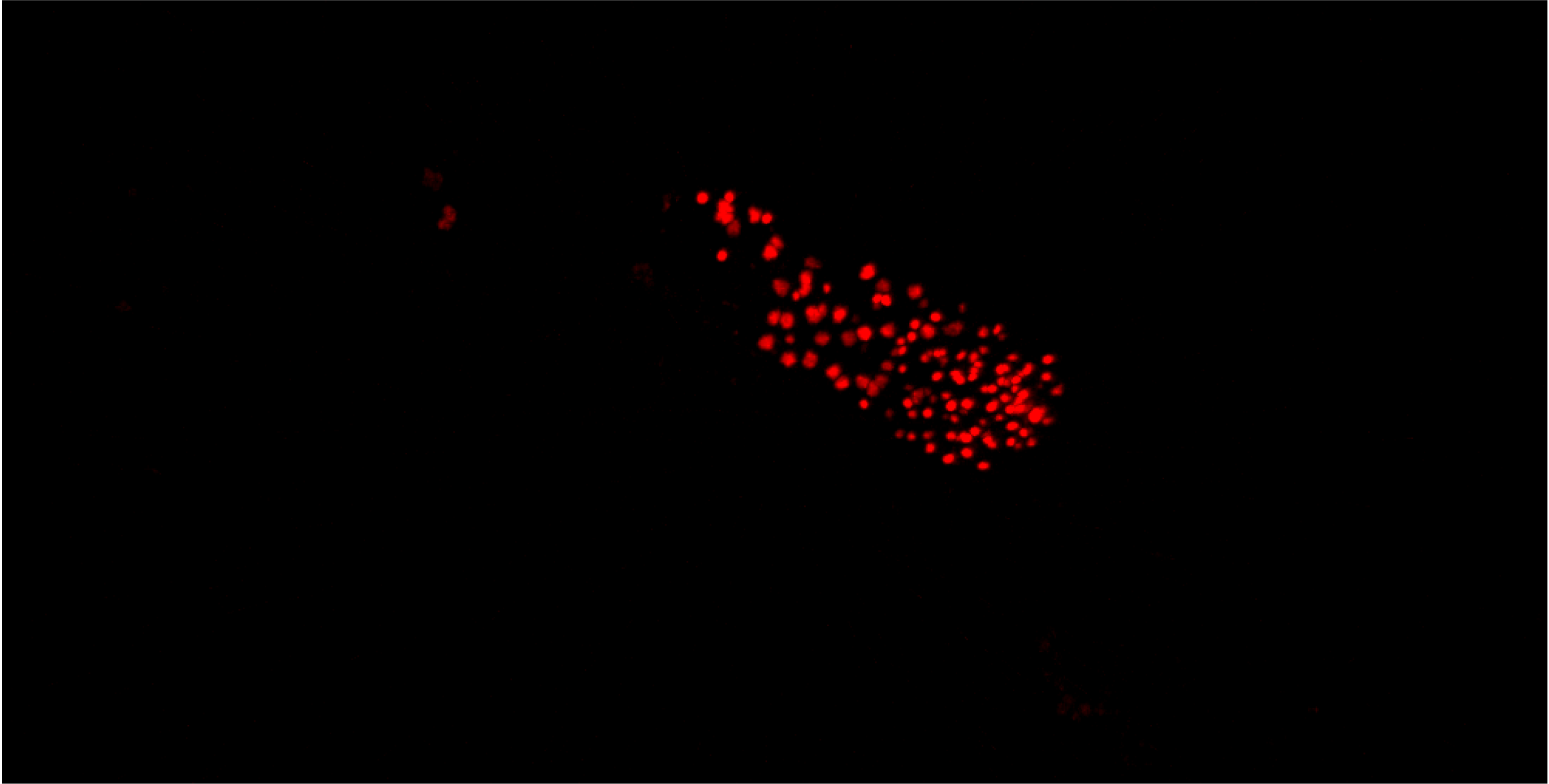

Supplement: Supplementary file 5 — Source data Fig. 3 [file 44318_2024_197_MOESM5_ESM.zip › SD figure 3/3I/3I.tif]

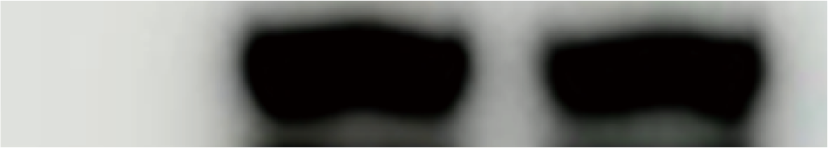

Supplement: Supplementary file 6 — Source data Fig. 4 [file 44318_2024_197_MOESM6_ESM.zip › SD figure 4/4D/4D replicate-1/IP HSP-4/IP HSP-4.tif]

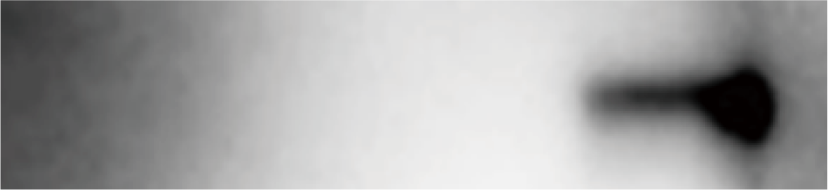

Supplement: Supplementary file 6 — Source data Fig. 4 [file 44318_2024_197_MOESM6_ESM.zip › SD figure 4/4D/4D replicate-1/IP TRA-2/IP TRA-2.tif]

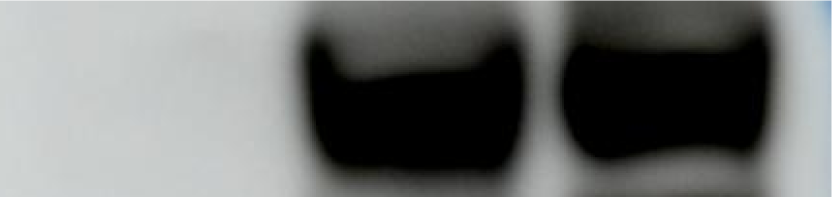

Supplement: Supplementary file 6 — Source data Fig. 4 [file 44318_2024_197_MOESM6_ESM.zip › SD figure 4/4D/4D replicate-1/lysate HSP-4/lysate HSP-4.tif]

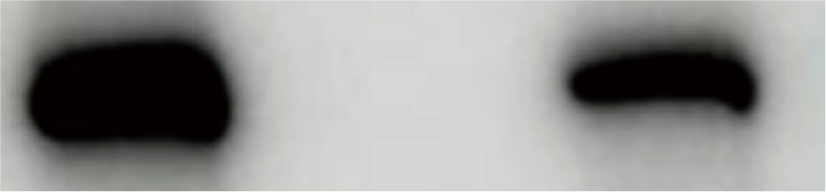

Supplement: Supplementary file 6 — Source data Fig. 4 [file 44318_2024_197_MOESM6_ESM.zip › SD figure 4/4D/4D replicate-1/lysate TRA-2/lysate TRA-2.tif]

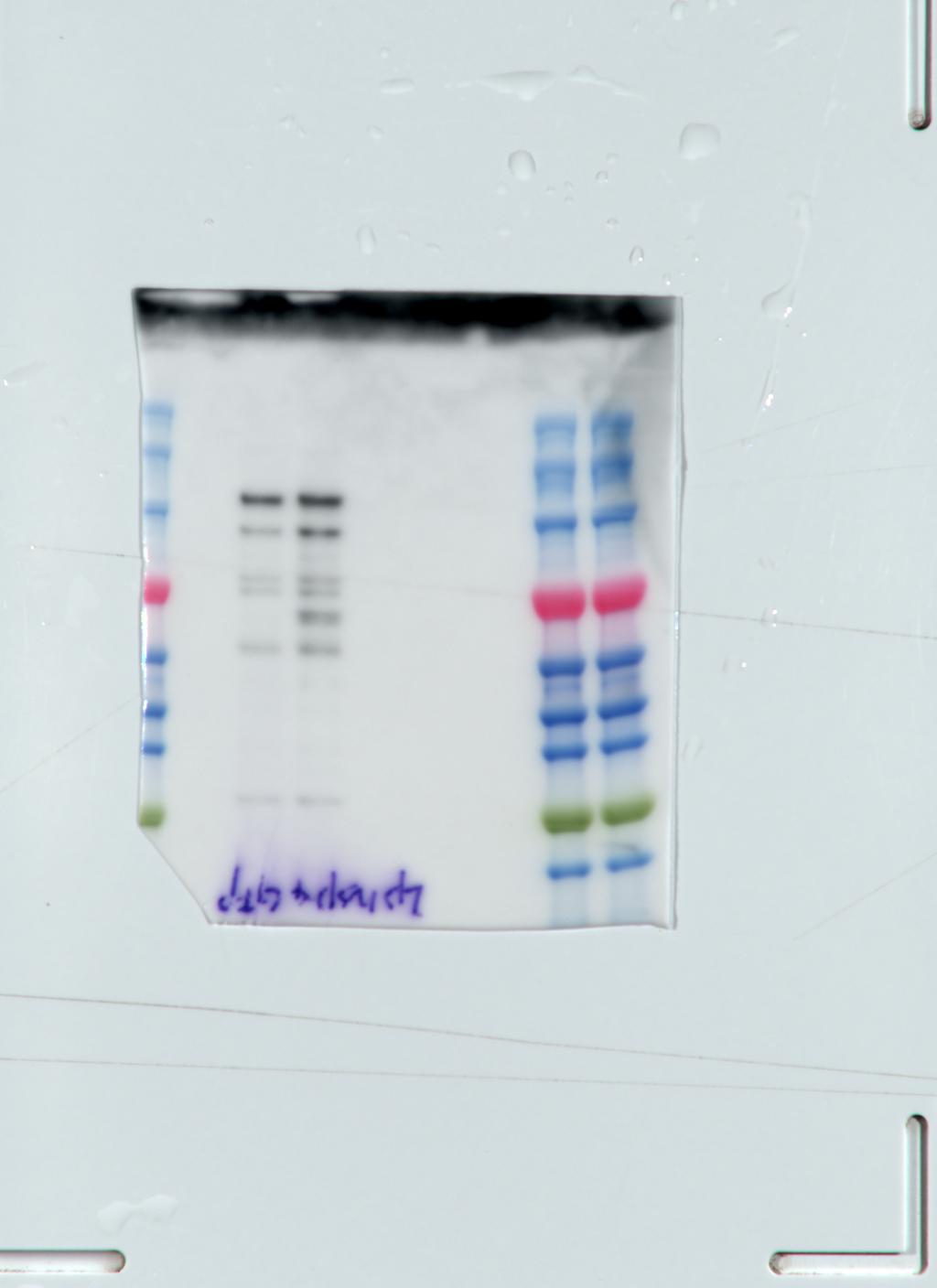

Supplement: Supplementary file 6 — Source data Fig. 4 [file 44318_2024_197_MOESM6_ESM.zip › SD figure 4/4D/4D replicate-2/IP HSP-4/IP HSP-4.tif]

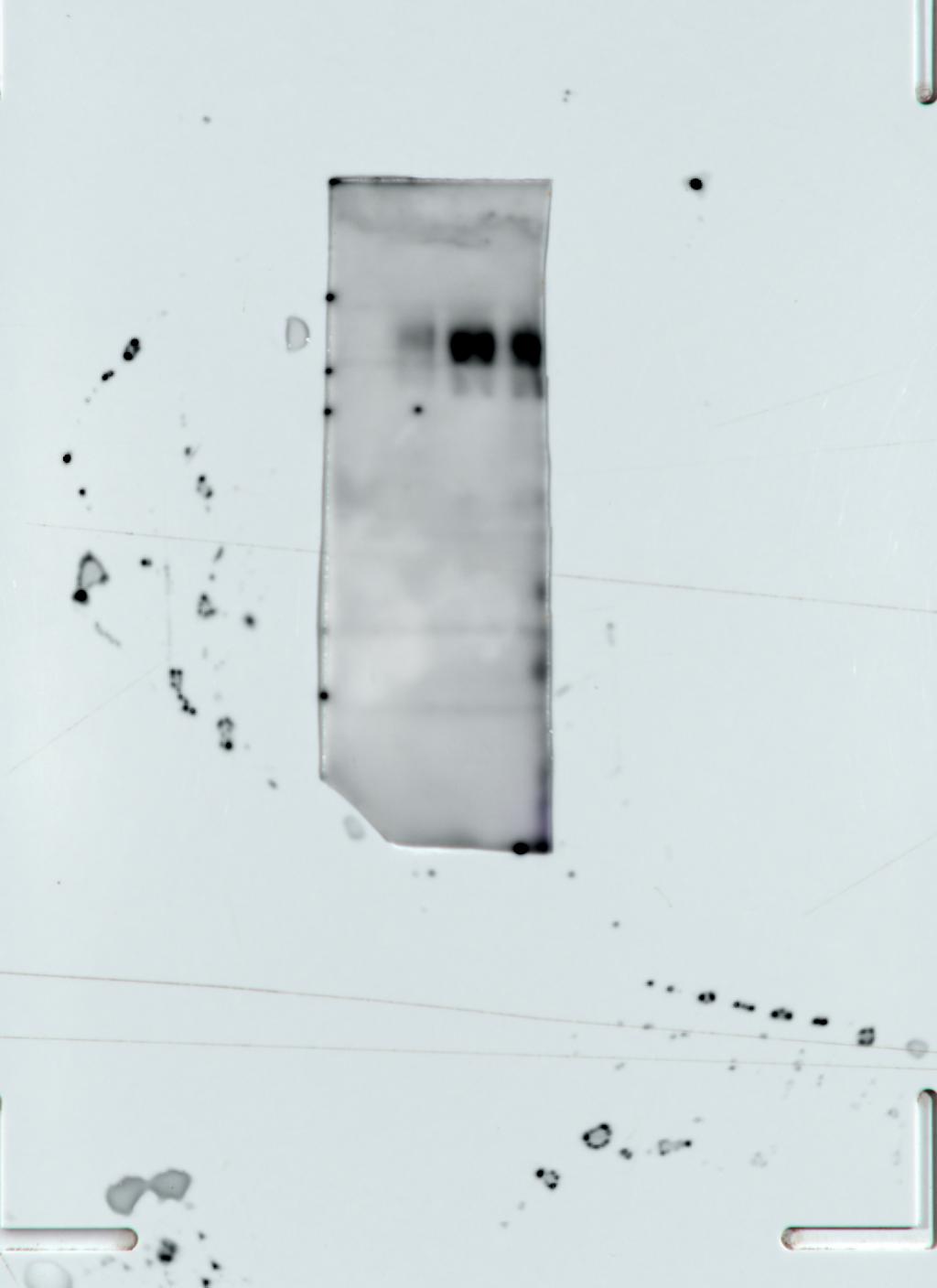

Supplement: Supplementary file 6 — Source data Fig. 4 [file 44318_2024_197_MOESM6_ESM.zip › SD figure 4/4D/4D replicate-2/IP TRA-2/IP TRA-2.tif]

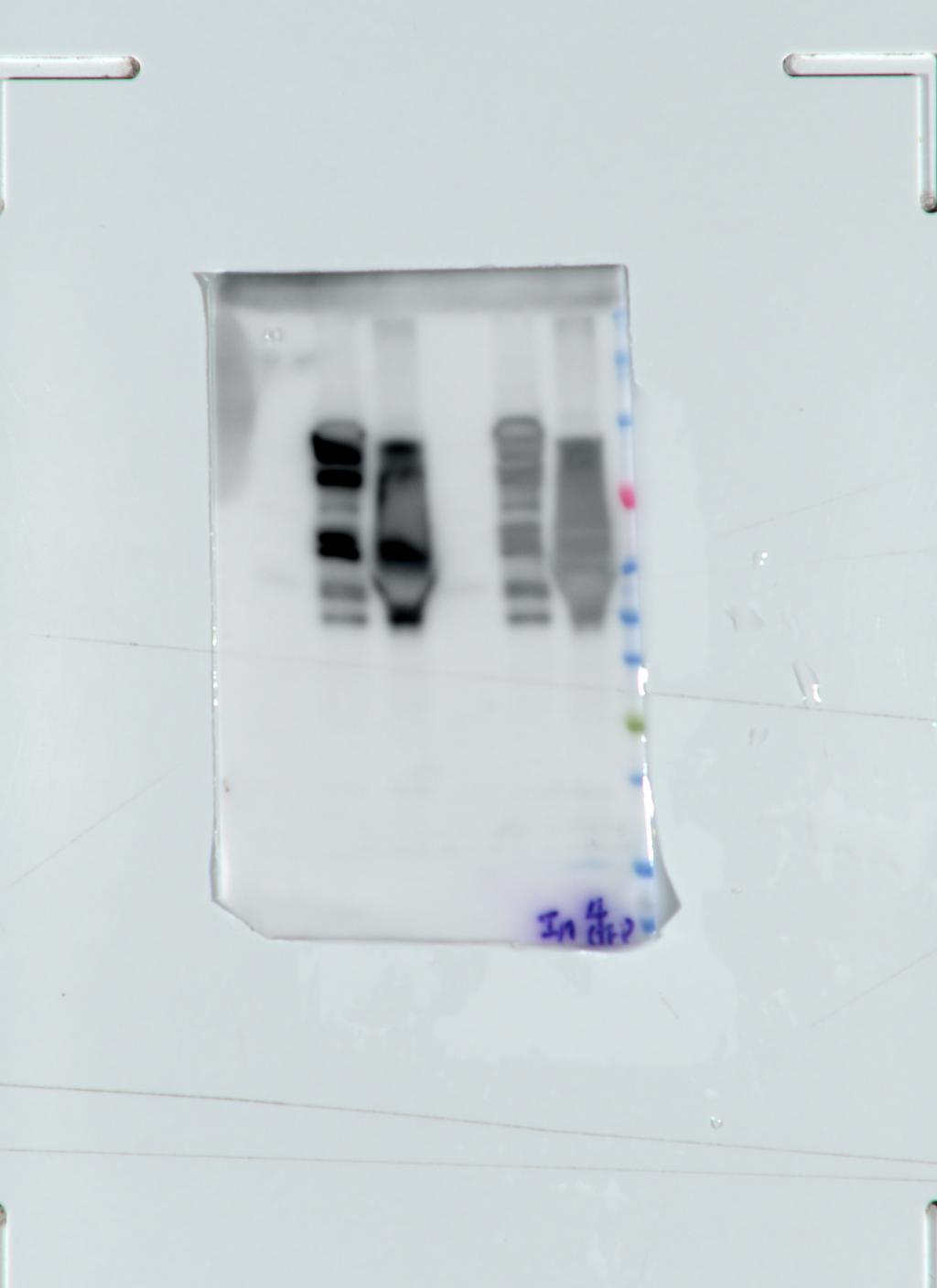

Supplement: Supplementary file 6 — Source data Fig. 4 [file 44318_2024_197_MOESM6_ESM.zip › SD figure 4/4D/4D replicate-2/lysate HSP-4/lysate HSP-4.tif]

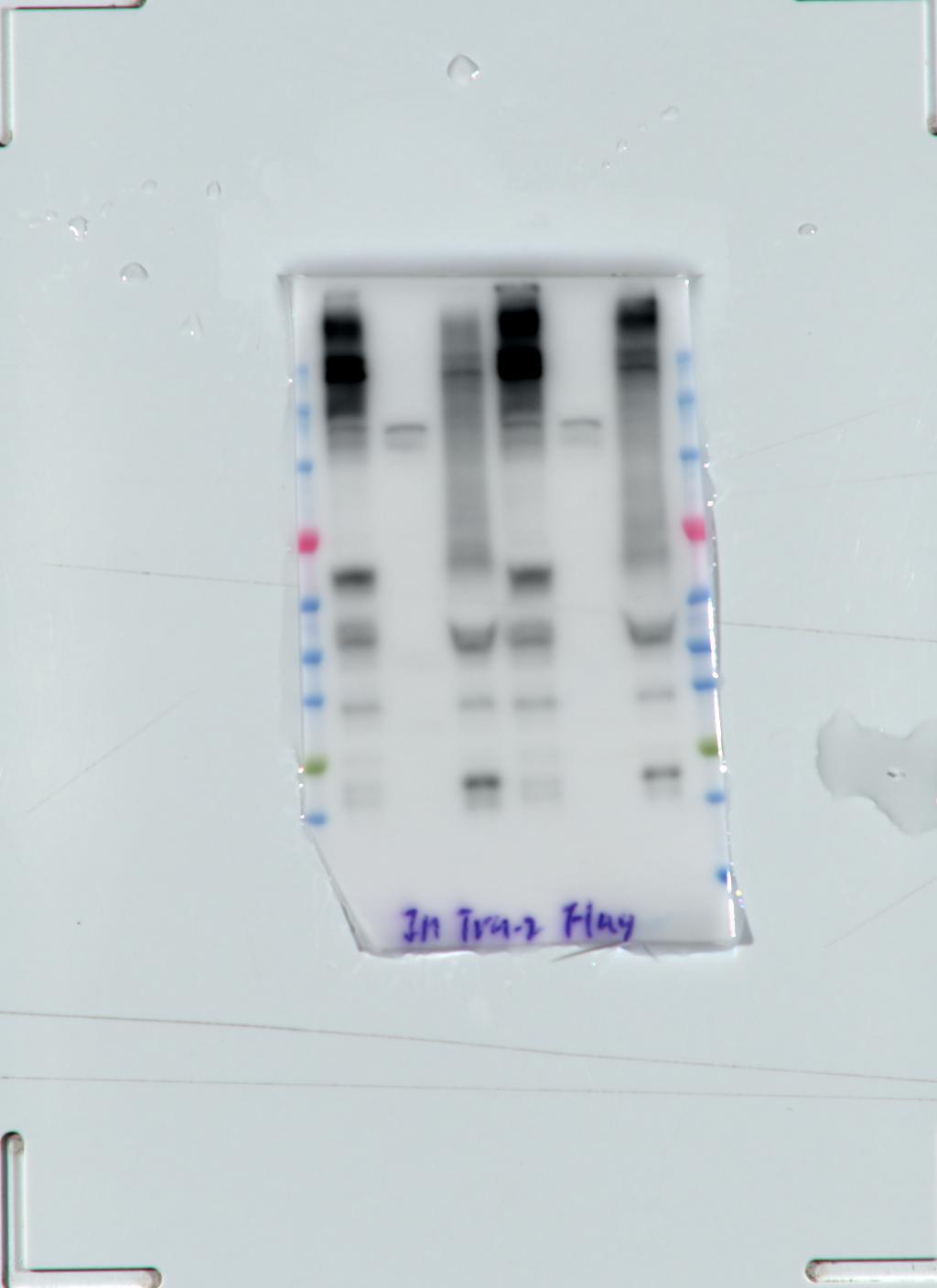

Supplement: Supplementary file 6 — Source data Fig. 4 [file 44318_2024_197_MOESM6_ESM.zip › SD figure 4/4D/4D replicate-2/lysate TRA-2/lysate TRA-2.tif]

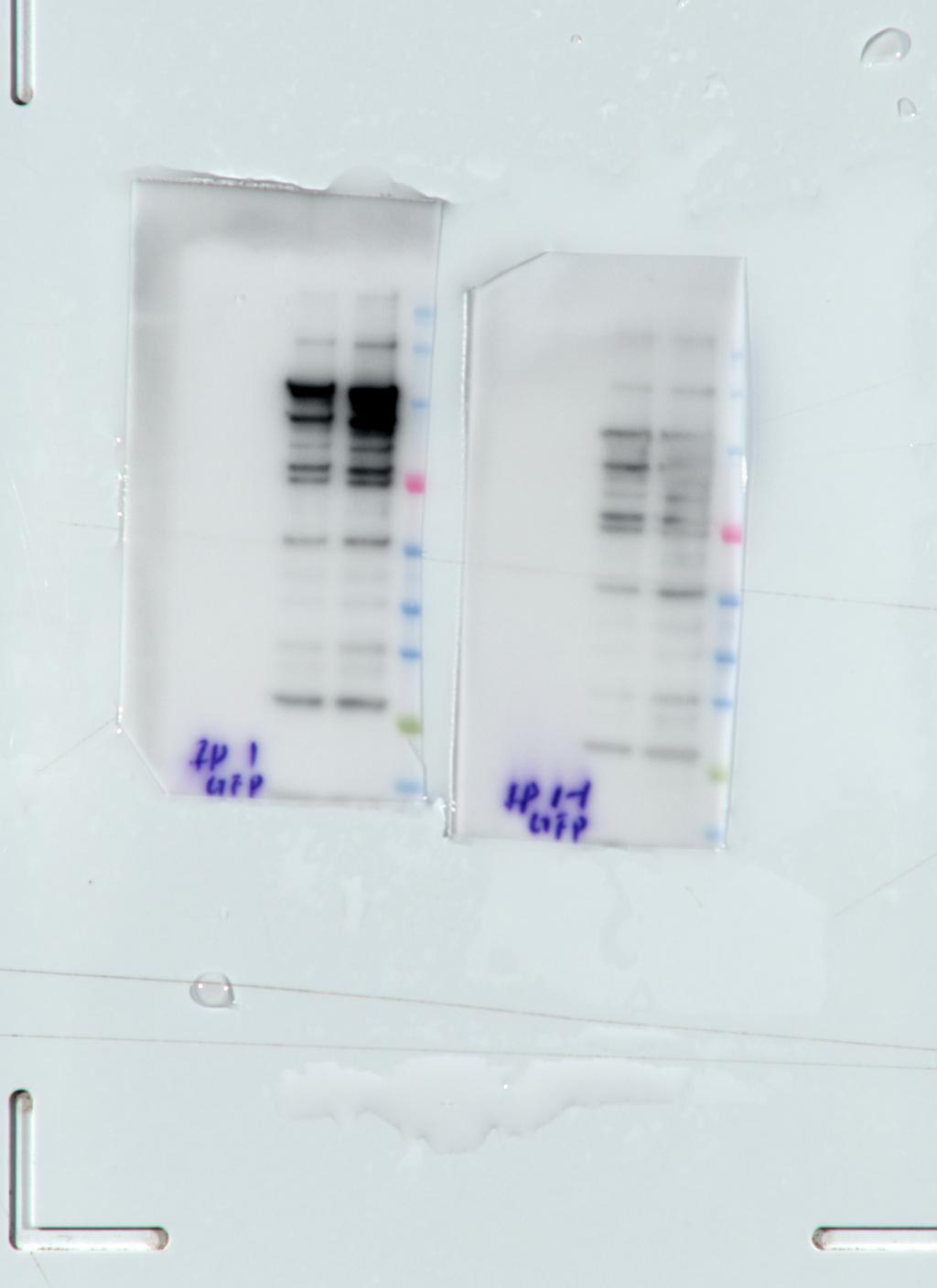

Supplement: Supplementary file 6 — Source data Fig. 4 [file 44318_2024_197_MOESM6_ESM.zip › SD figure 4/4D/4D replicate-3/IP HSP-4/IP HSP-4.tif]

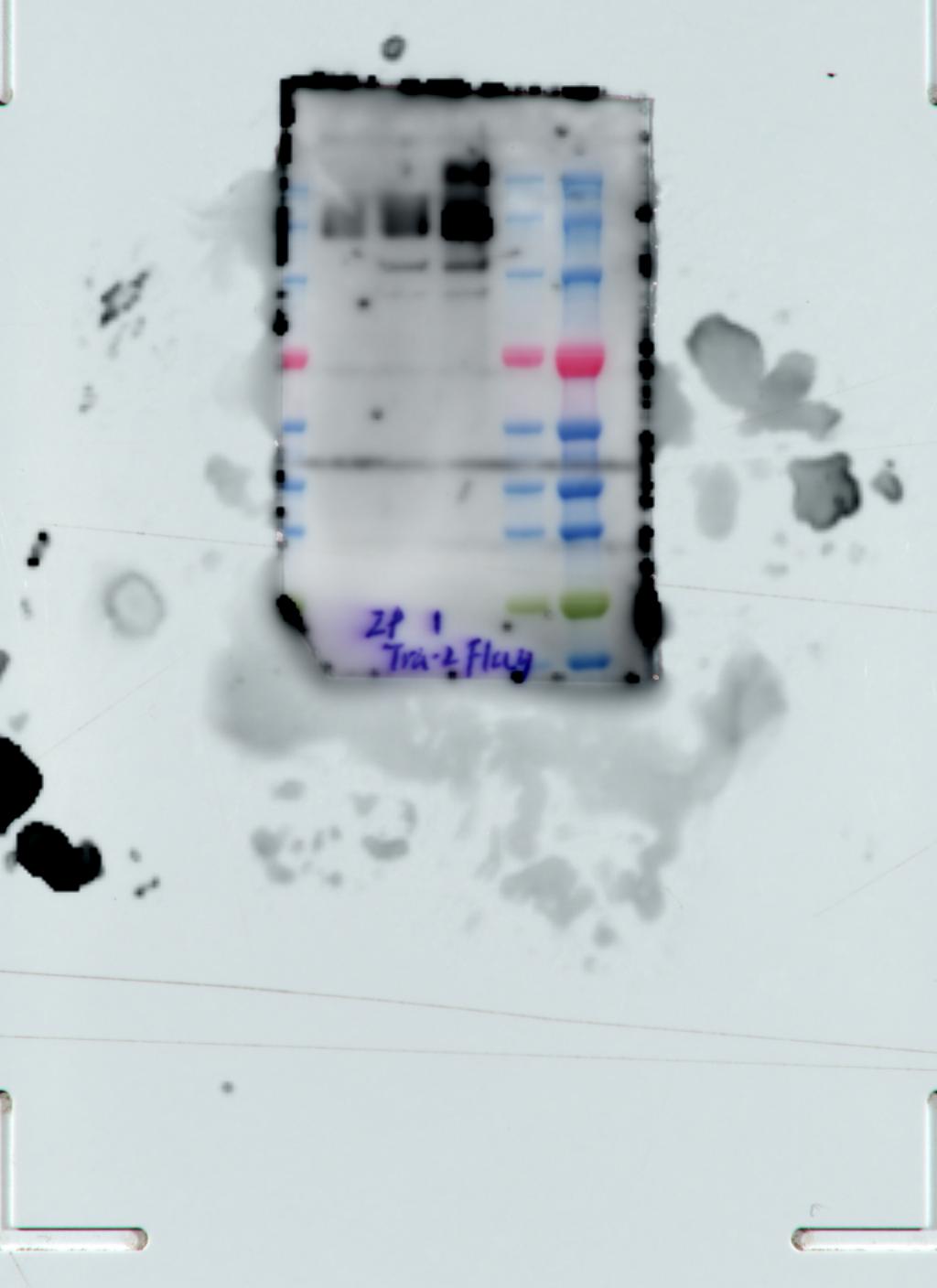

Supplement: Supplementary file 6 — Source data Fig. 4 [file 44318_2024_197_MOESM6_ESM.zip › SD figure 4/4D/4D replicate-3/IP TRA-2/IP TRA-2.tif]

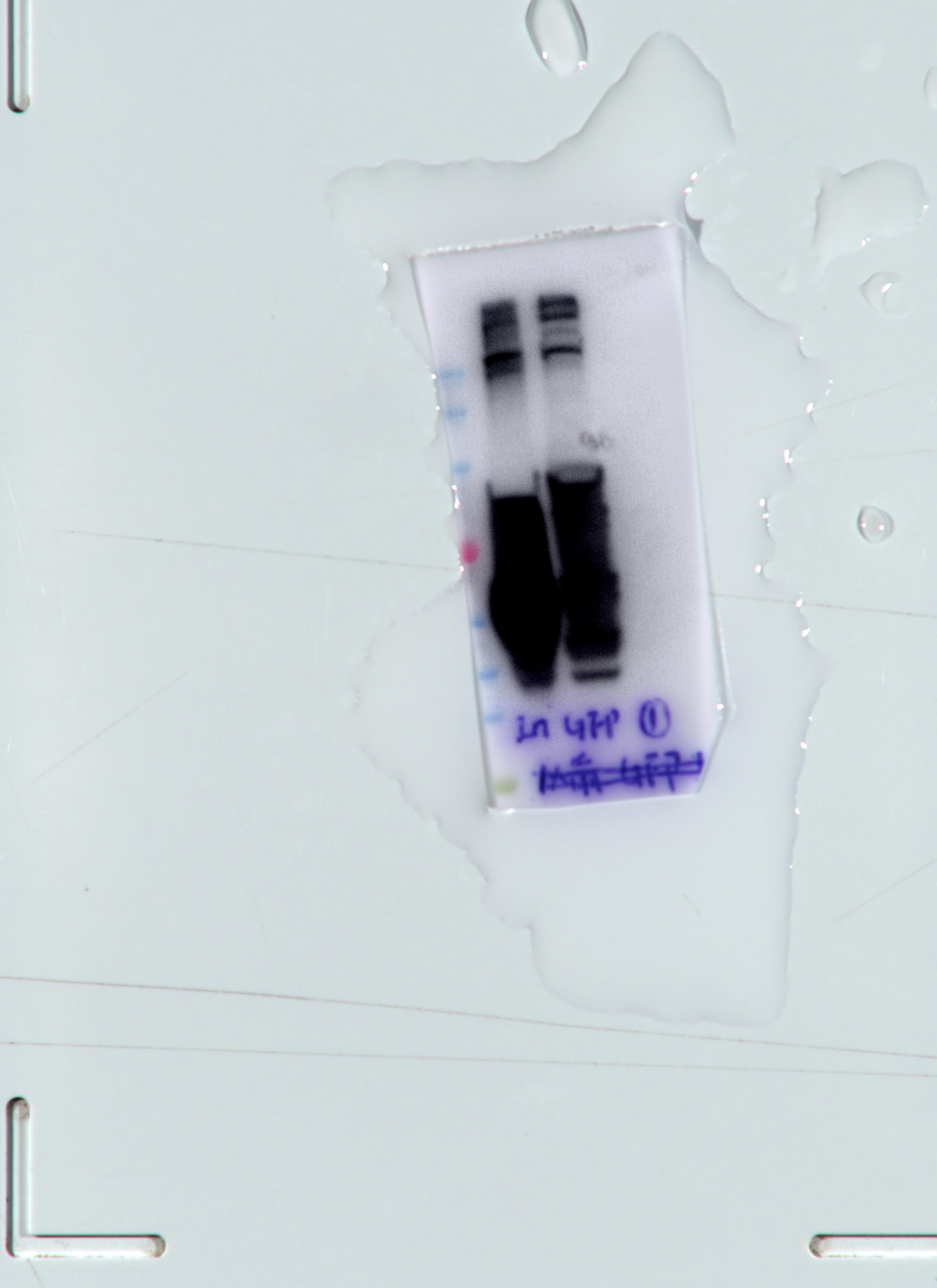

Supplement: Supplementary file 6 — Source data Fig. 4 [file 44318_2024_197_MOESM6_ESM.zip › SD figure 4/4D/4D replicate-3/lysate HSP-4/lysate HSP-4.tif]

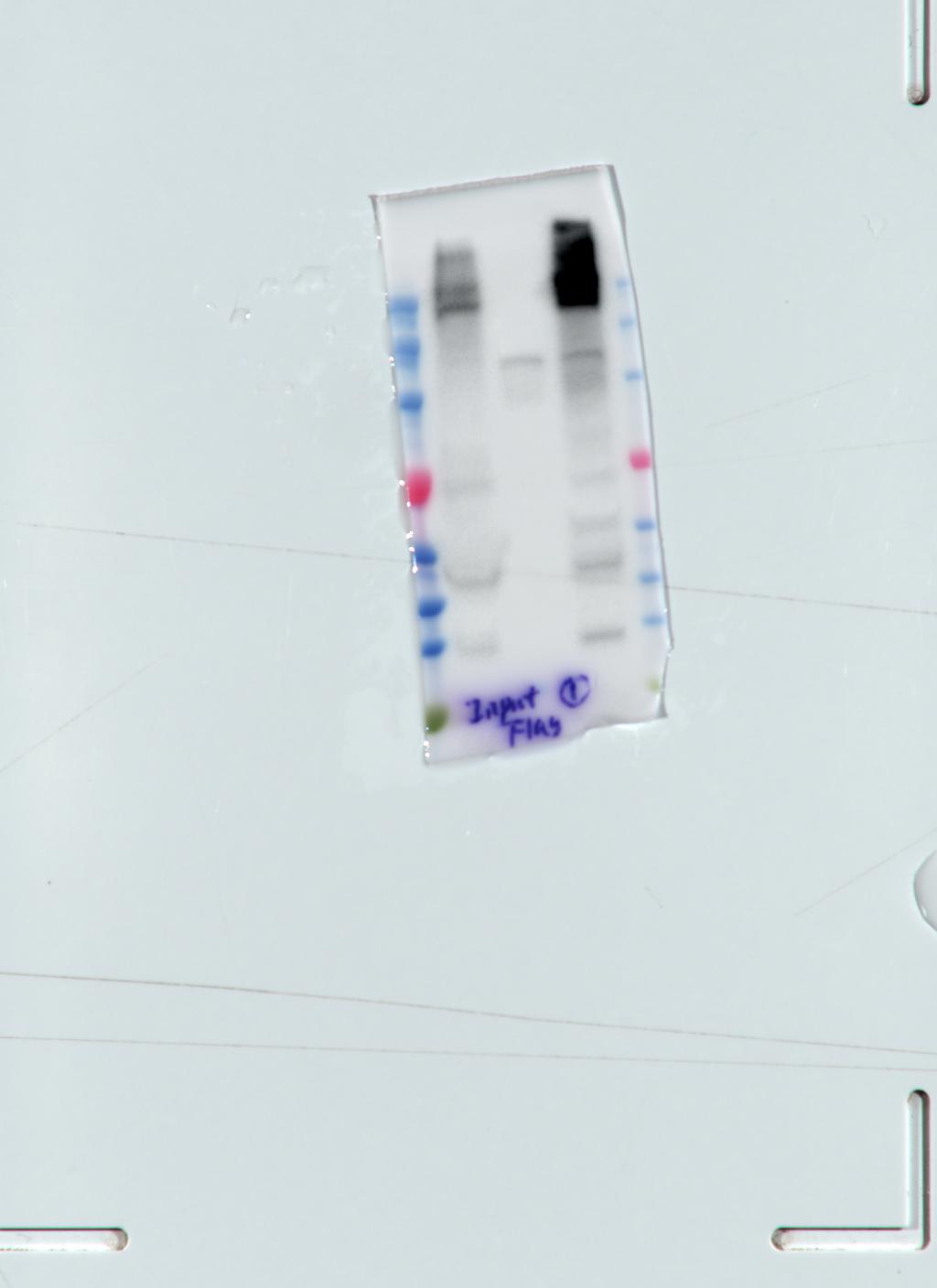

Supplement: Supplementary file 6 — Source data Fig. 4 [file 44318_2024_197_MOESM6_ESM.zip › SD figure 4/4D/4D replicate-3/lysate TRA-2/lysate TRA-2.tif]

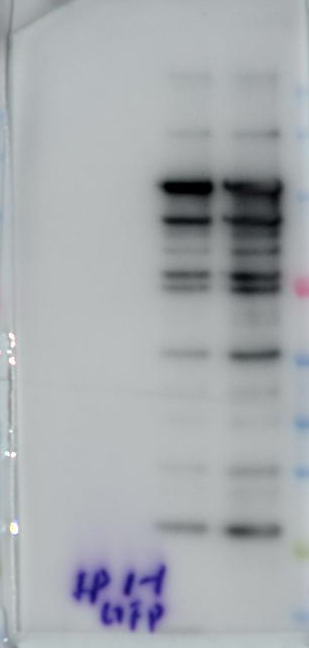

Supplement: Supplementary file 6 — Source data Fig. 4 [file 44318_2024_197_MOESM6_ESM.zip › SD figure 4/4D/4D replicate-4/IP HSP-4/IP HSP-4.tif]

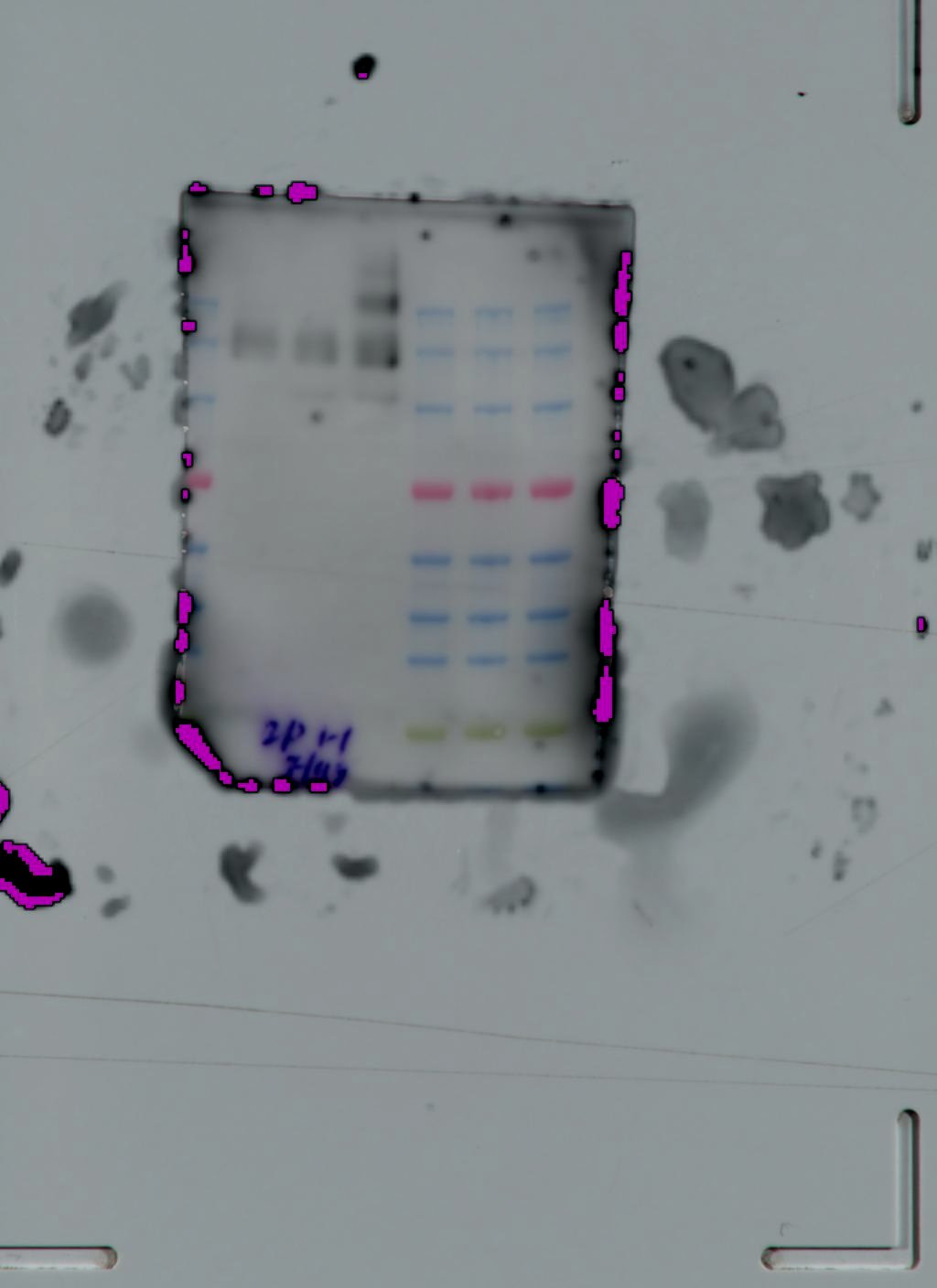

Supplement: Supplementary file 6 — Source data Fig. 4 [file 44318_2024_197_MOESM6_ESM.zip › SD figure 4/4D/4D replicate-4/IP TRA-2/IP TRA-2.tif]

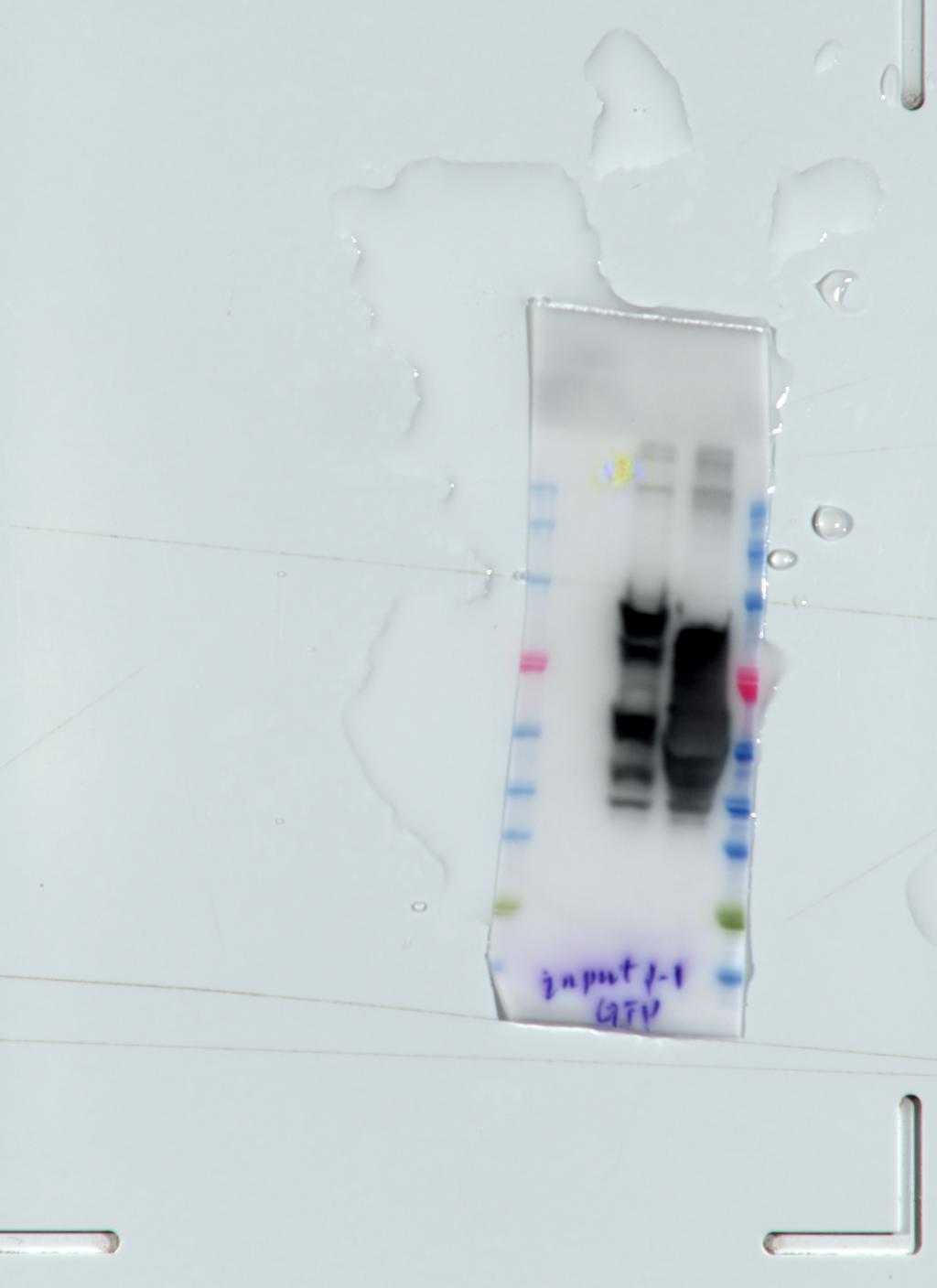

Supplement: Supplementary file 6 — Source data Fig. 4 [file 44318_2024_197_MOESM6_ESM.zip › SD figure 4/4D/4D replicate-4/lysate HSP-4/lysate HSP-4.tif]

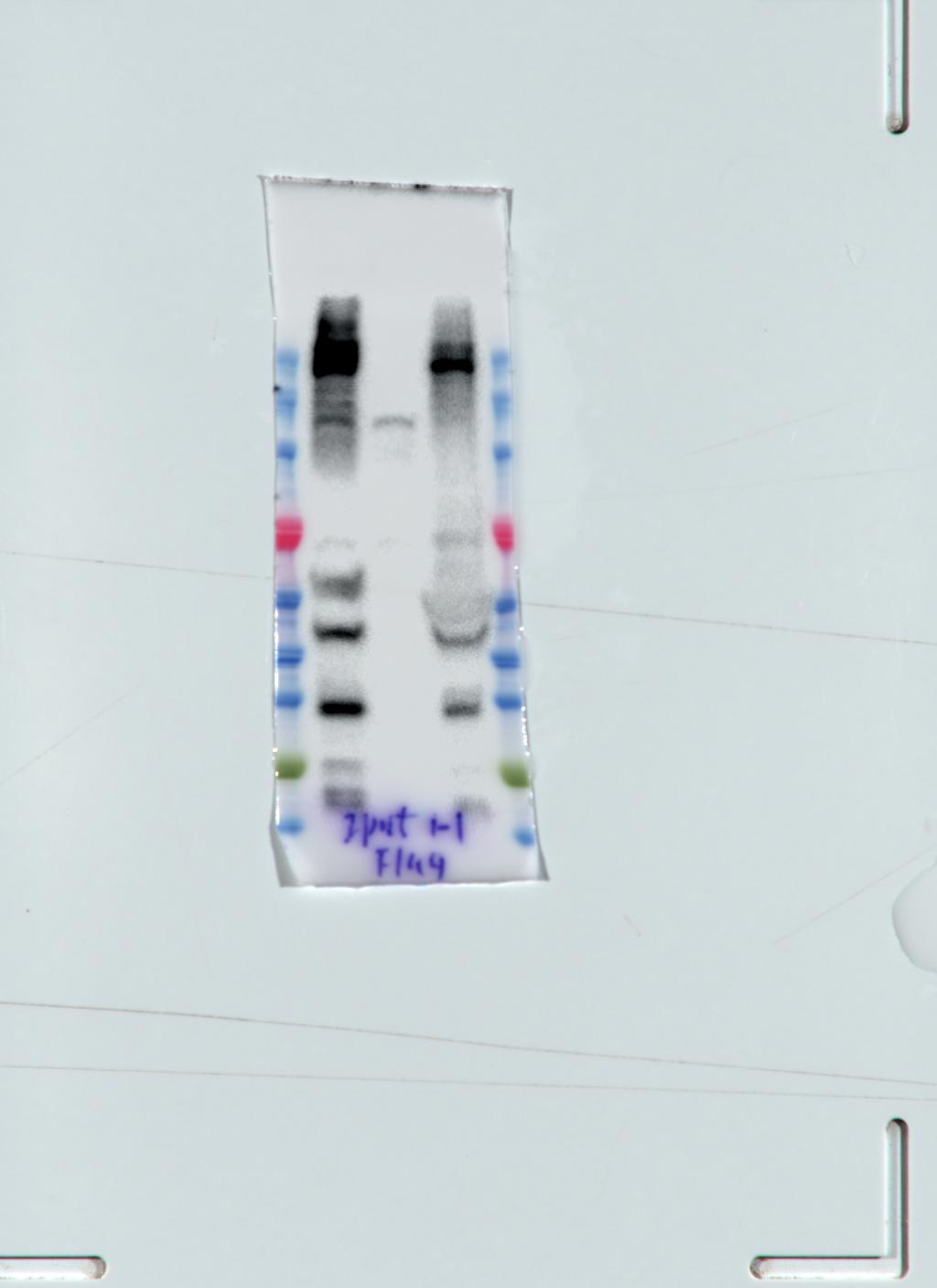

Supplement: Supplementary file 6 — Source data Fig. 4 [file 44318_2024_197_MOESM6_ESM.zip › SD figure 4/4D/4D replicate-4/lysate TRA-2/lysate TRA-2.tif]

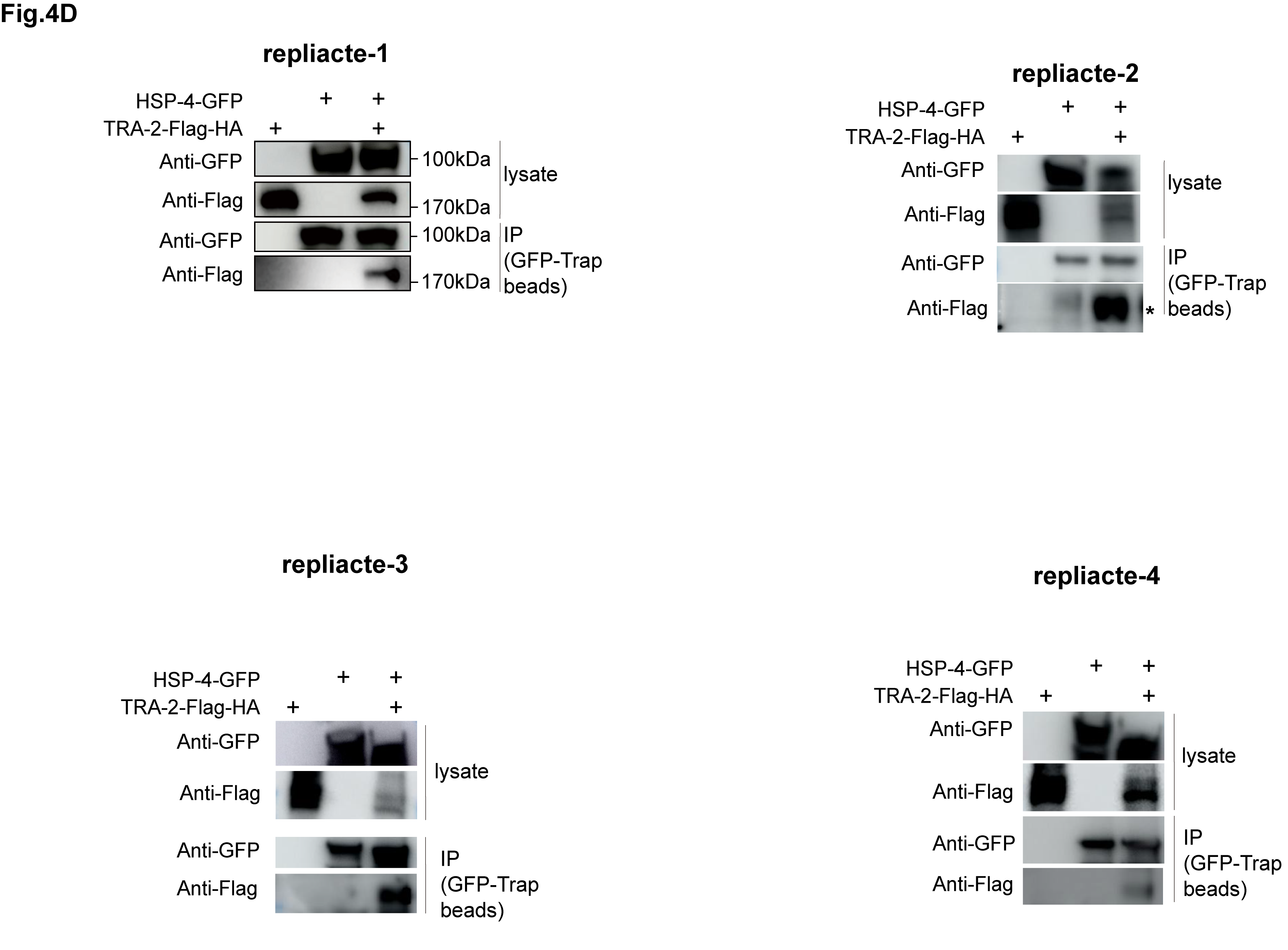

Supplement: Supplementary file 6 — Source data Fig. 4 [file 44318_2024_197_MOESM6_ESM.zip › SD figure 4/4D/4D.tif]

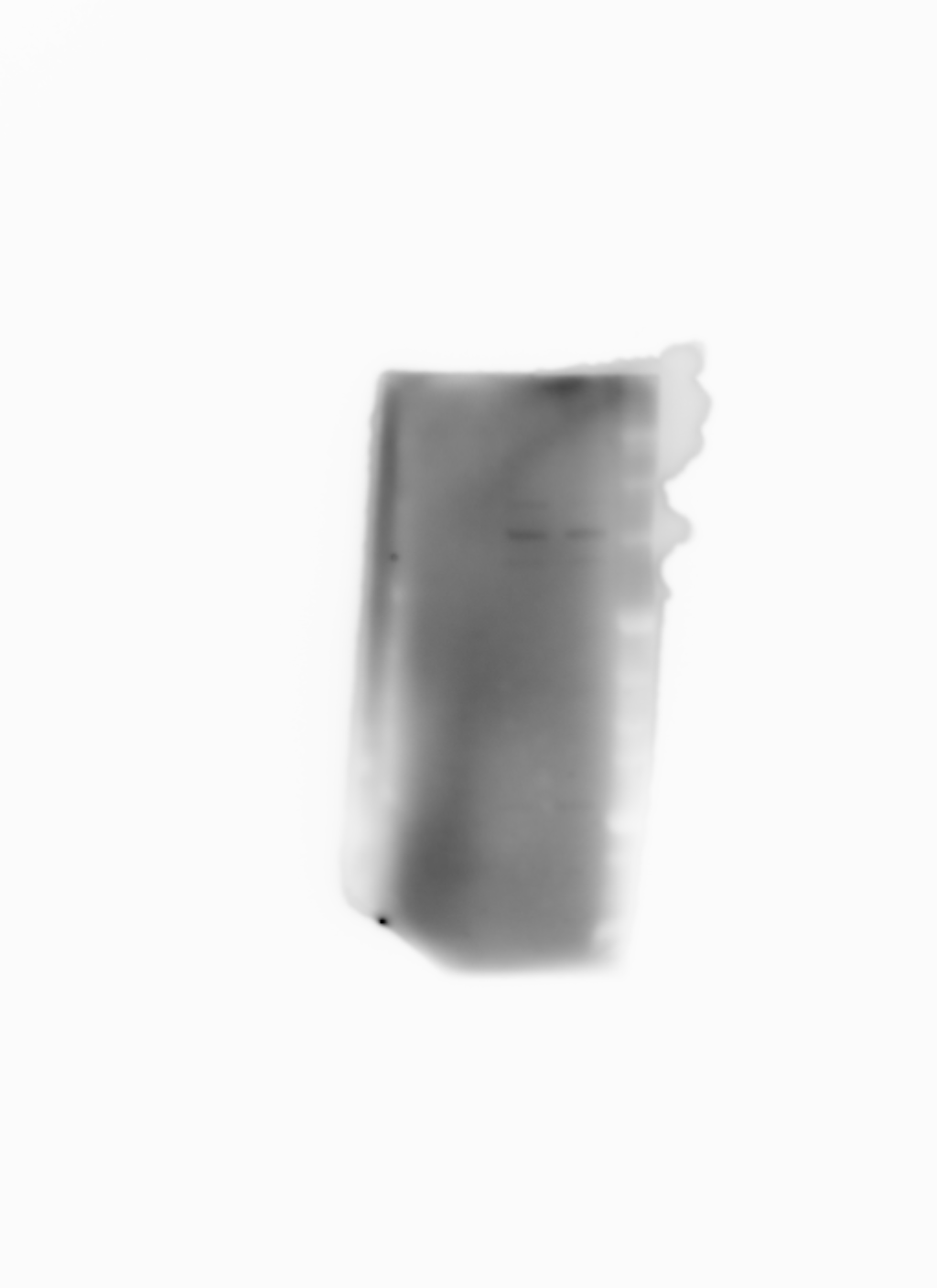

Supplement: Supplementary file 6 — Source data Fig. 4 [file 44318_2024_197_MOESM6_ESM.zip › SD figure 4/4E/4E replicate-1/IP FEM-3/IP FEM-3.tif]

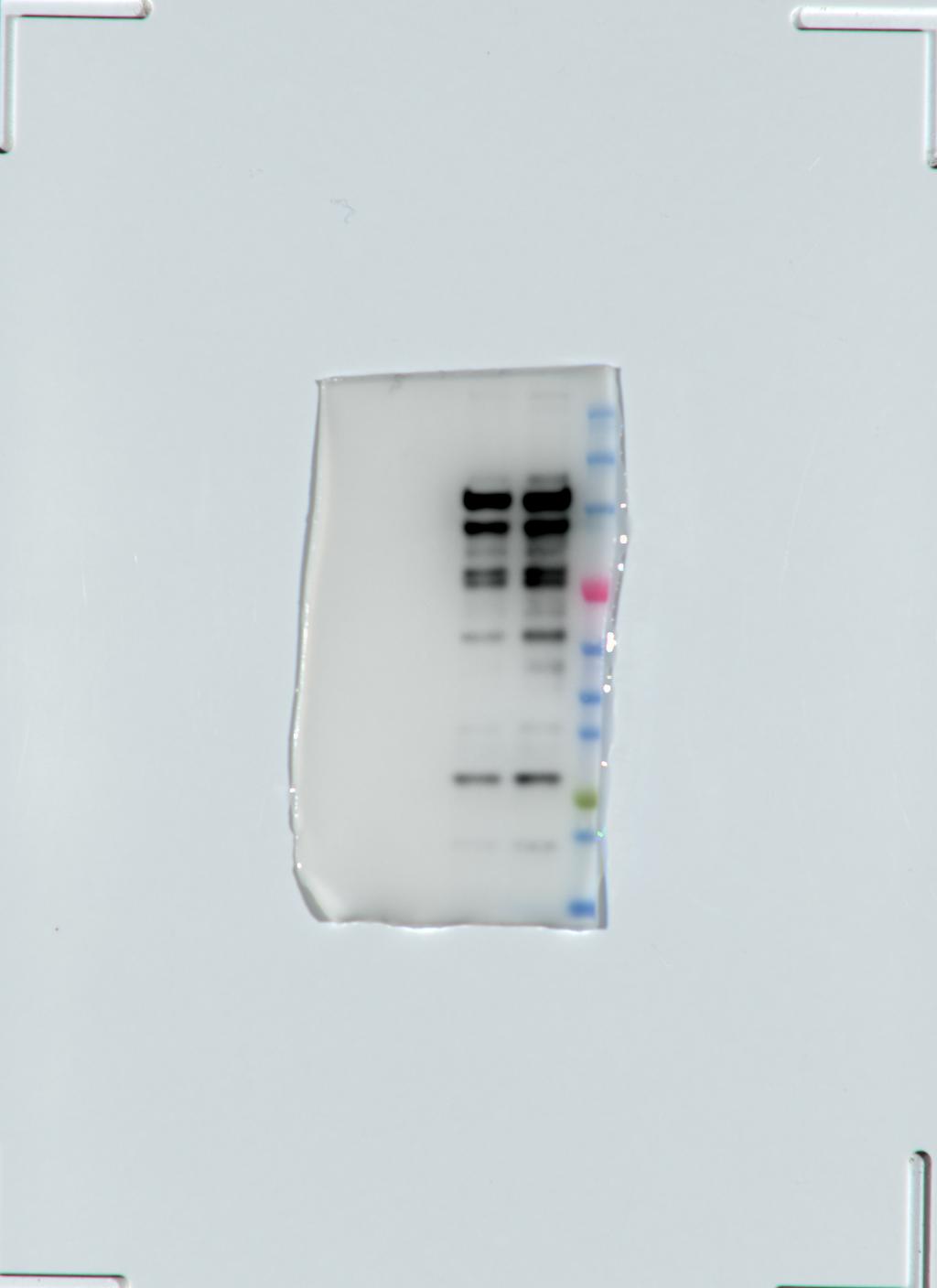

Supplement: Supplementary file 6 — Source data Fig. 4 [file 44318_2024_197_MOESM6_ESM.zip › SD figure 4/4E/4E replicate-1/IP HSP-4/IP HSP-4.tif]

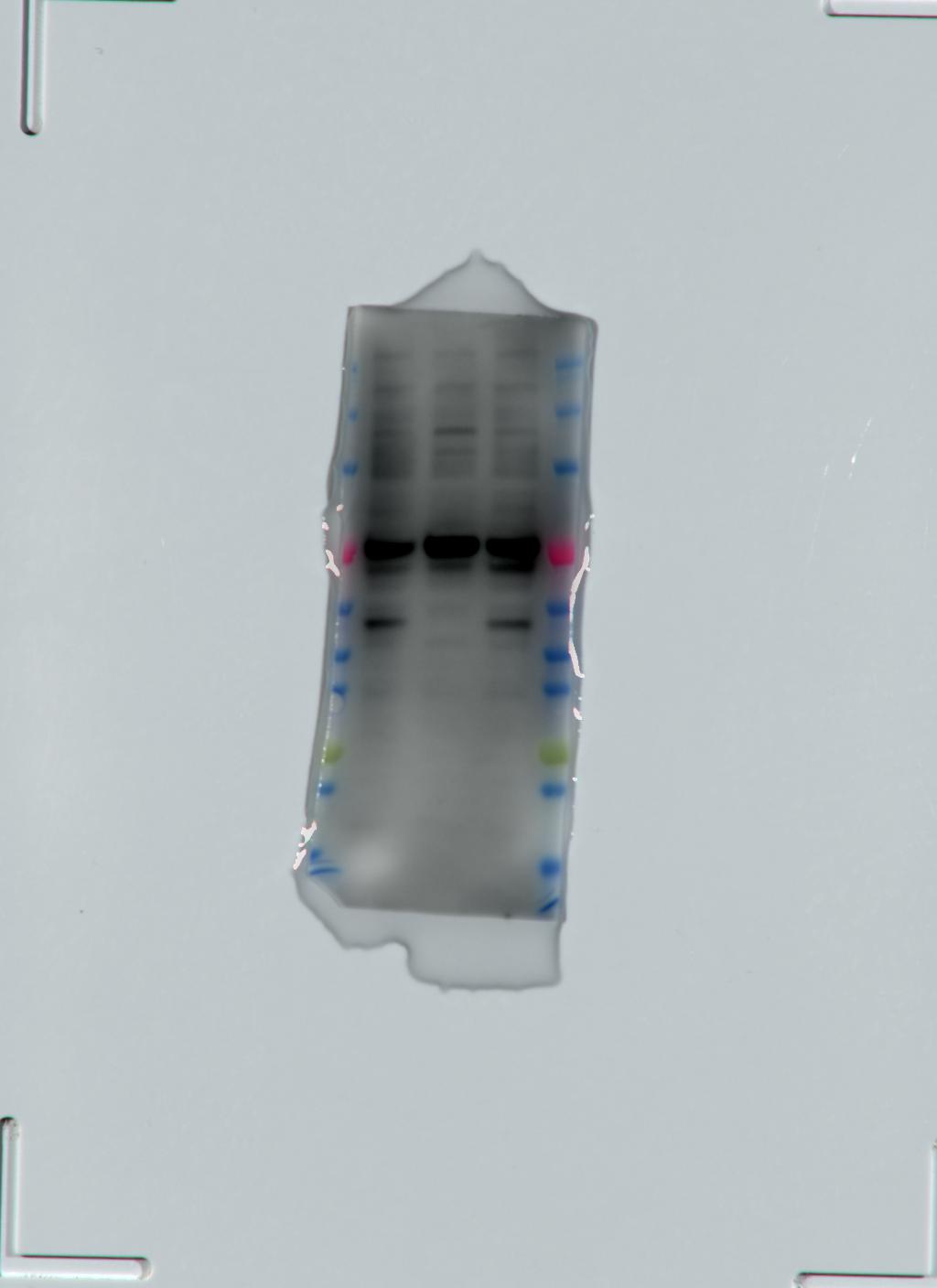

Supplement: Supplementary file 6 — Source data Fig. 4 [file 44318_2024_197_MOESM6_ESM.zip › SD figure 4/4E/4E replicate-1/lysate FEM-3/lysate FEM-3.tif]

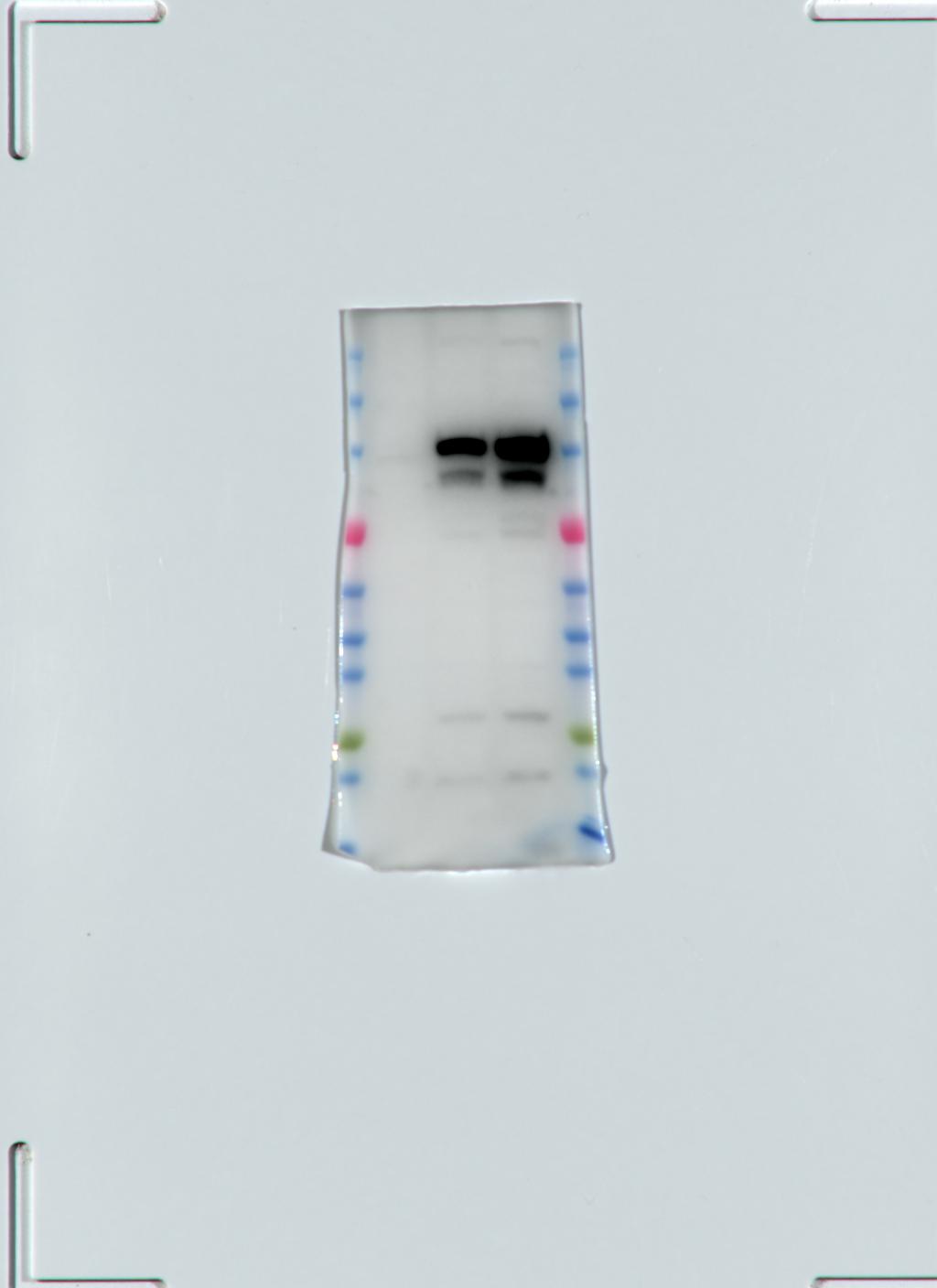

Supplement: Supplementary file 6 — Source data Fig. 4 [file 44318_2024_197_MOESM6_ESM.zip › SD figure 4/4E/4E replicate-1/lysate HSP-4/lysate HSP-4.tif]

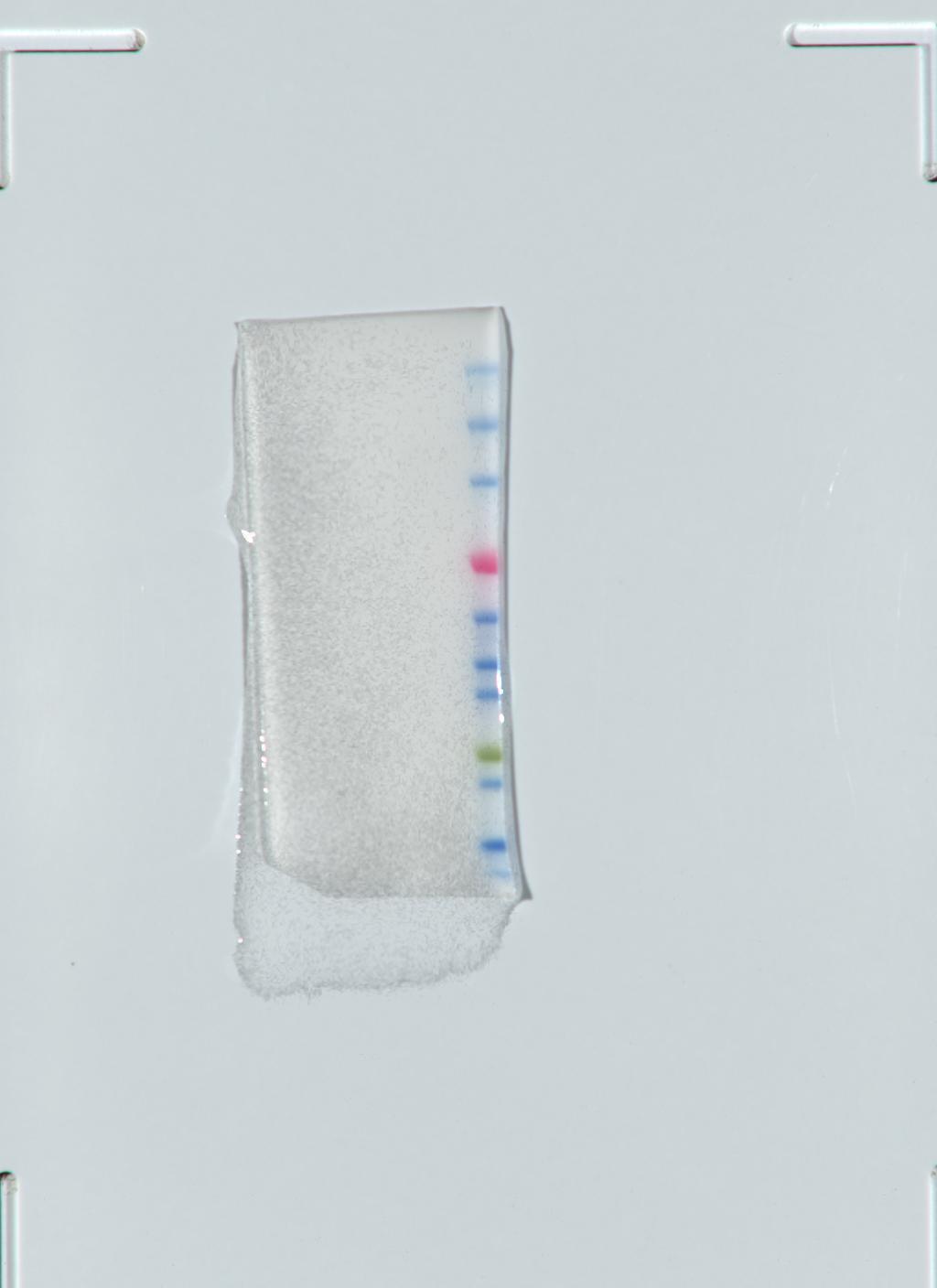

Supplement: Supplementary file 6 — Source data Fig. 4 [file 44318_2024_197_MOESM6_ESM.zip › SD figure 4/4E/4E replicate-2/IP FEM-3/IP FEM-3.tif]

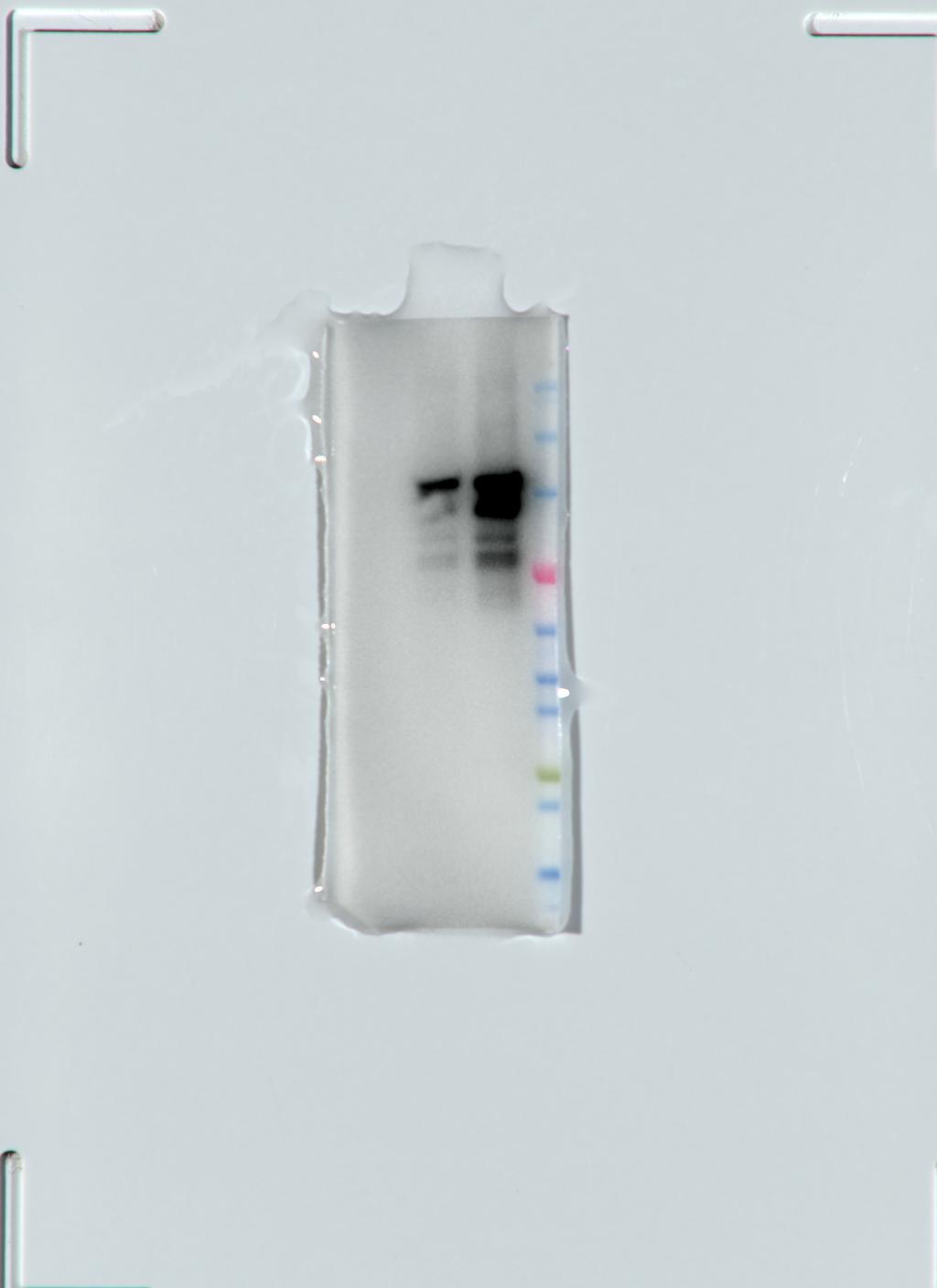

Supplement: Supplementary file 6 — Source data Fig. 4 [file 44318_2024_197_MOESM6_ESM.zip › SD figure 4/4E/4E replicate-2/IP HSP-4/IP HSP-4.tif]

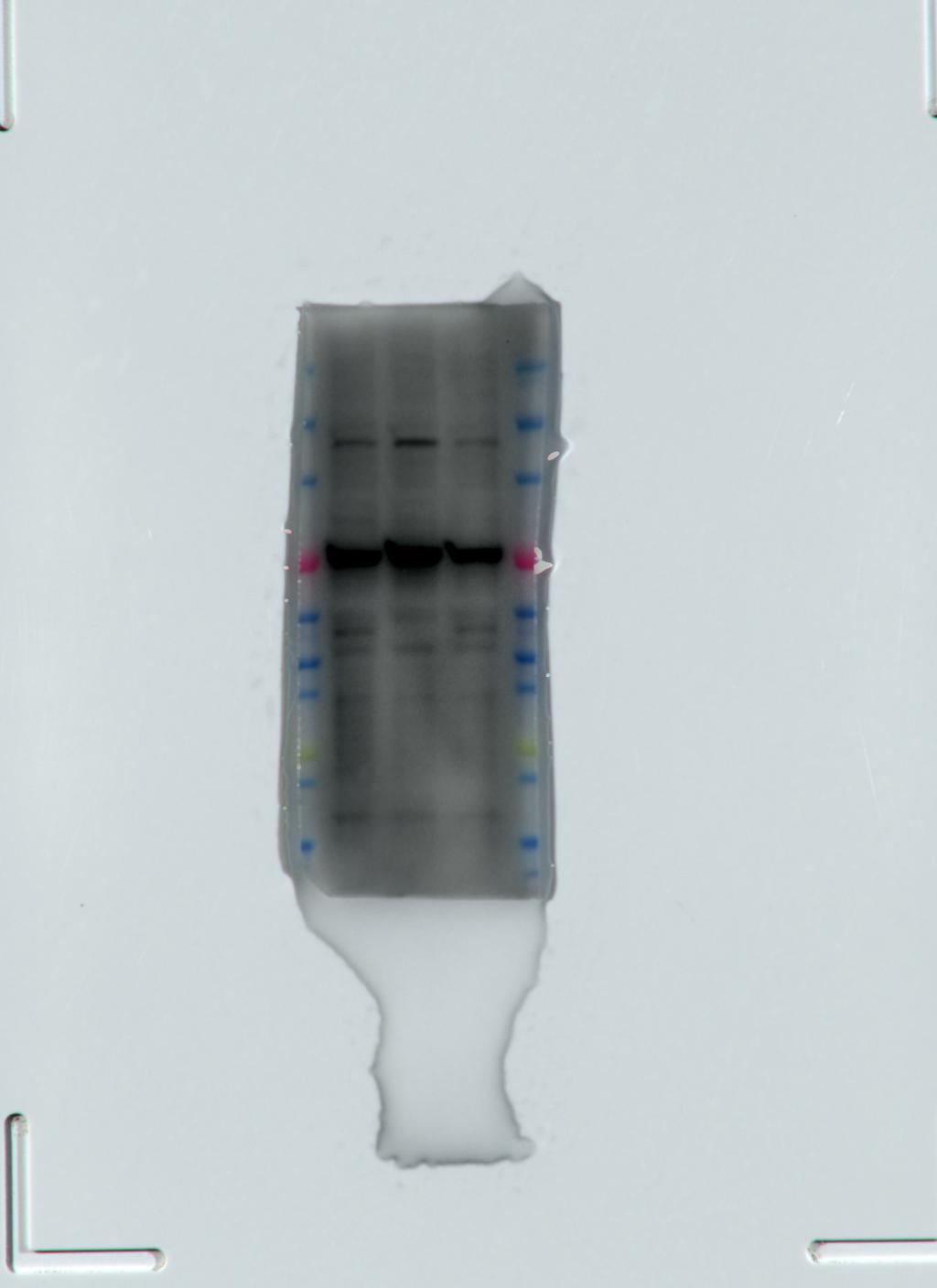

Supplement: Supplementary file 6 — Source data Fig. 4 [file 44318_2024_197_MOESM6_ESM.zip › SD figure 4/4E/4E replicate-2/lysate FEM-3/lysate FEM-3.tif]

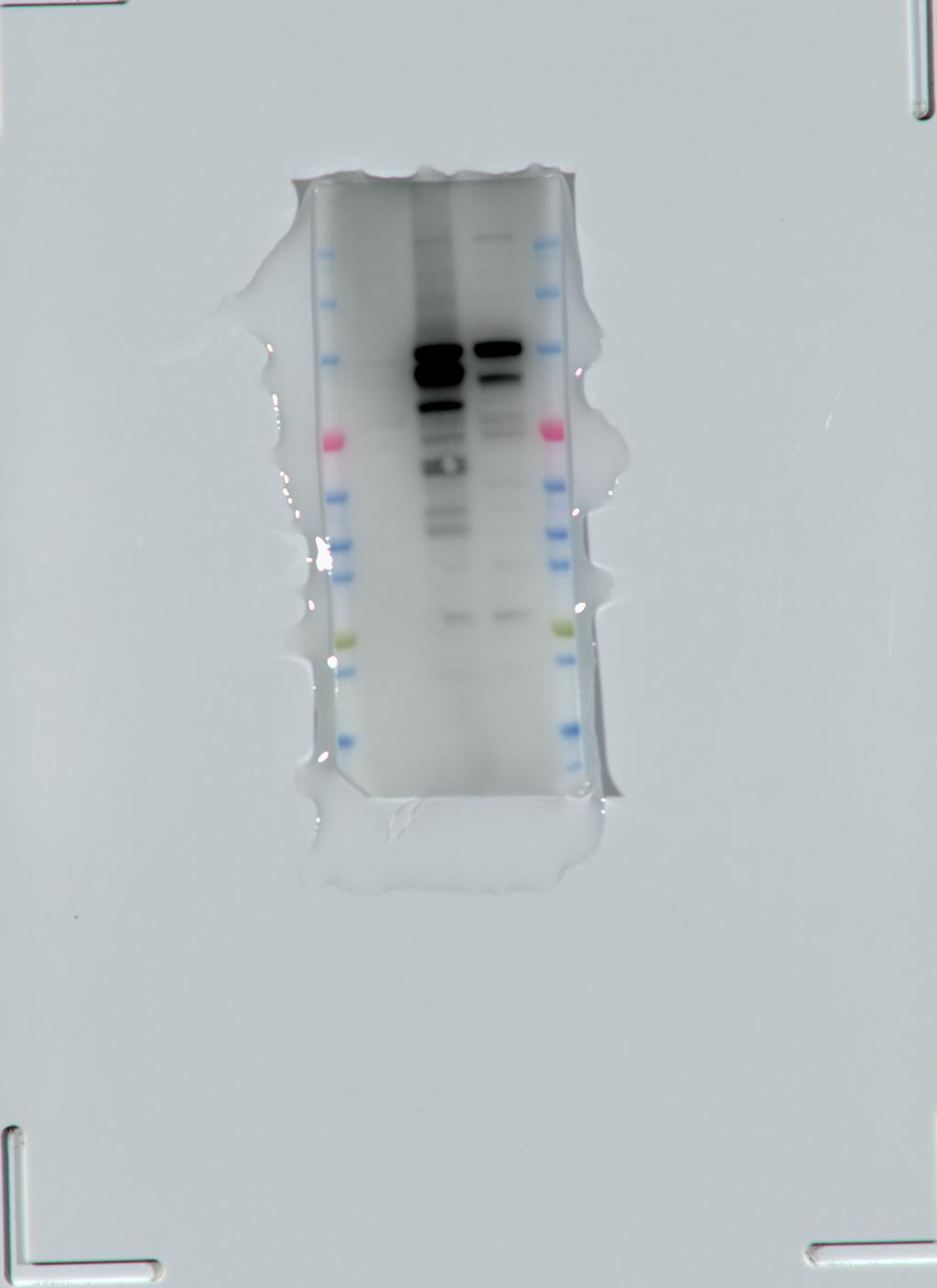

Supplement: Supplementary file 6 — Source data Fig. 4 [file 44318_2024_197_MOESM6_ESM.zip › SD figure 4/4E/4E replicate-2/lysate HSP-4/lysate HSP-4.tif]

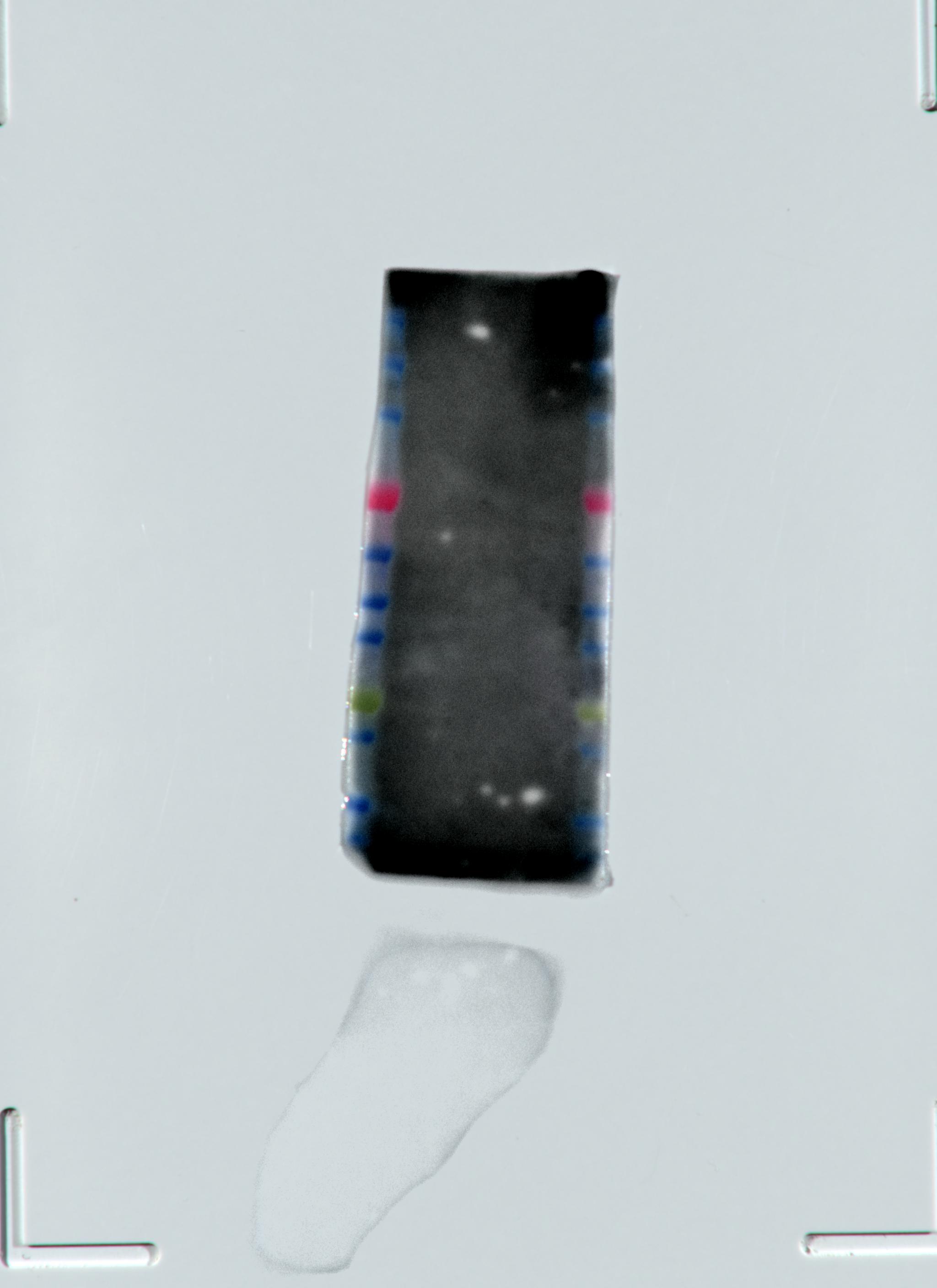

Supplement: Supplementary file 6 — Source data Fig. 4 [file 44318_2024_197_MOESM6_ESM.zip › SD figure 4/4E/4E replicate-3/IP FEM-3/IP FEM-3.tif]

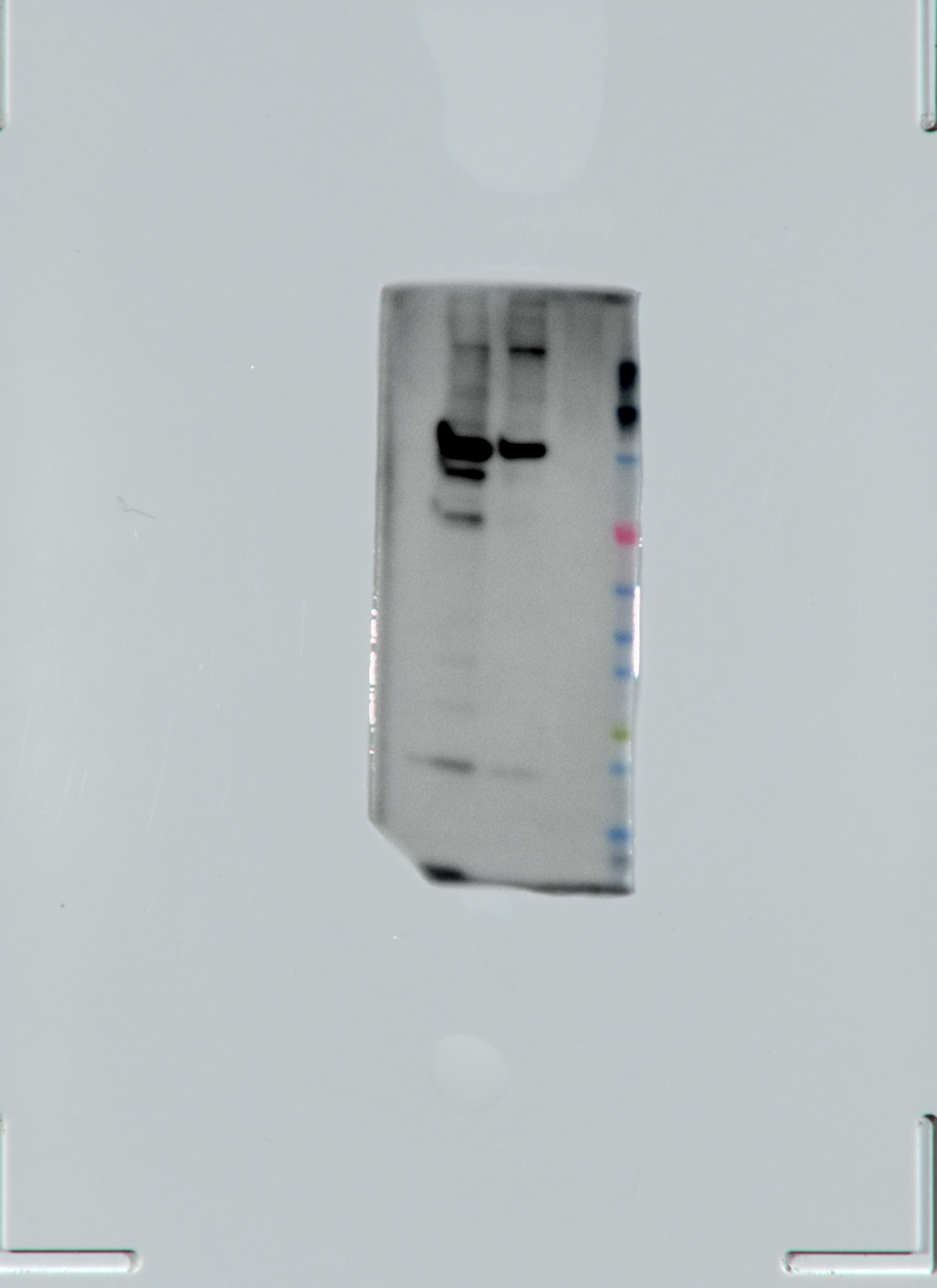

Supplement: Supplementary file 6 — Source data Fig. 4 [file 44318_2024_197_MOESM6_ESM.zip › SD figure 4/4E/4E replicate-3/IP HSP-4/IP HSP-4.tif]

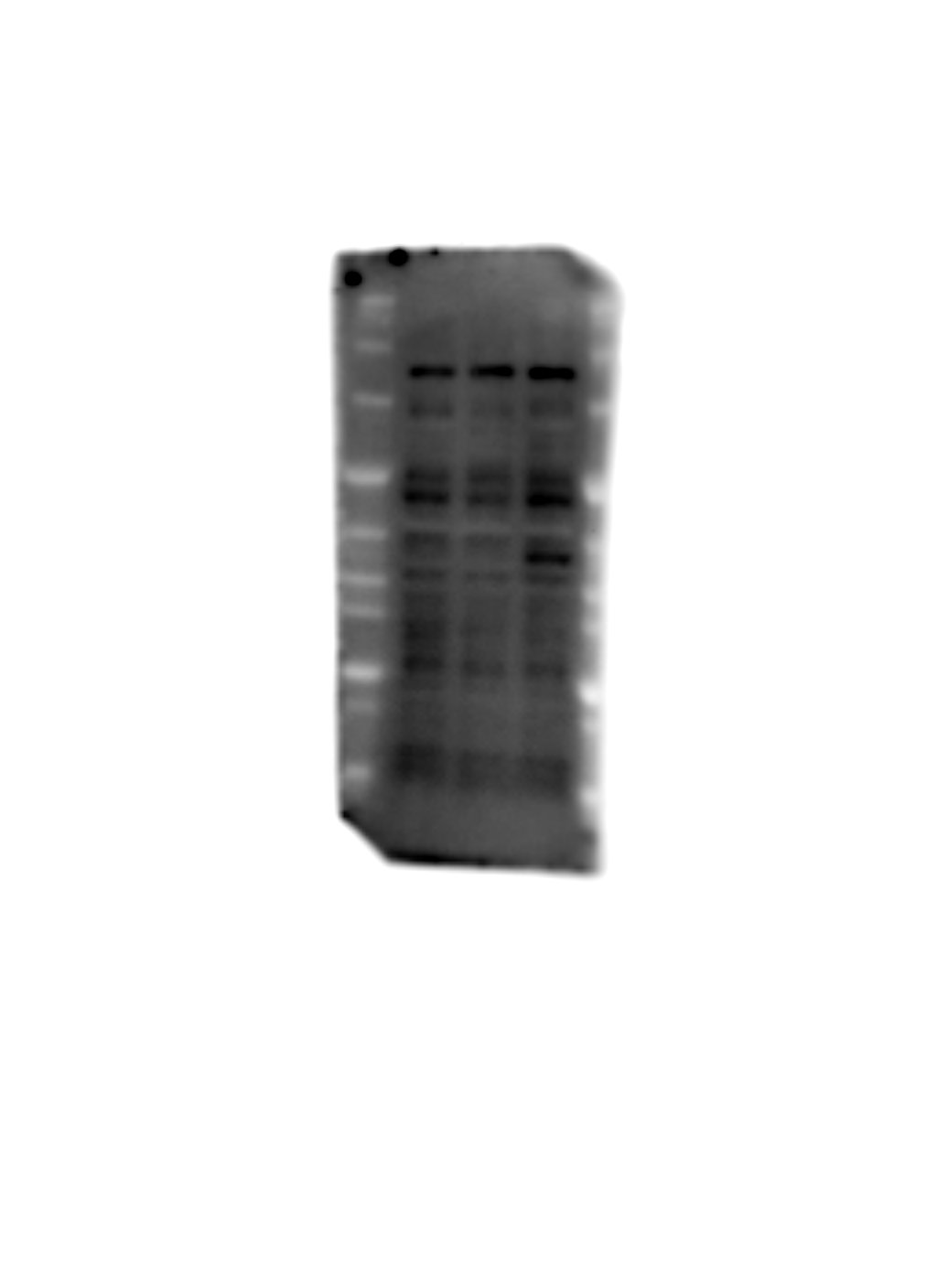

Supplement: Supplementary file 6 — Source data Fig. 4 [file 44318_2024_197_MOESM6_ESM.zip › SD figure 4/4E/4E replicate-3/lysate FEM-3/lysate FEM-3.tif]

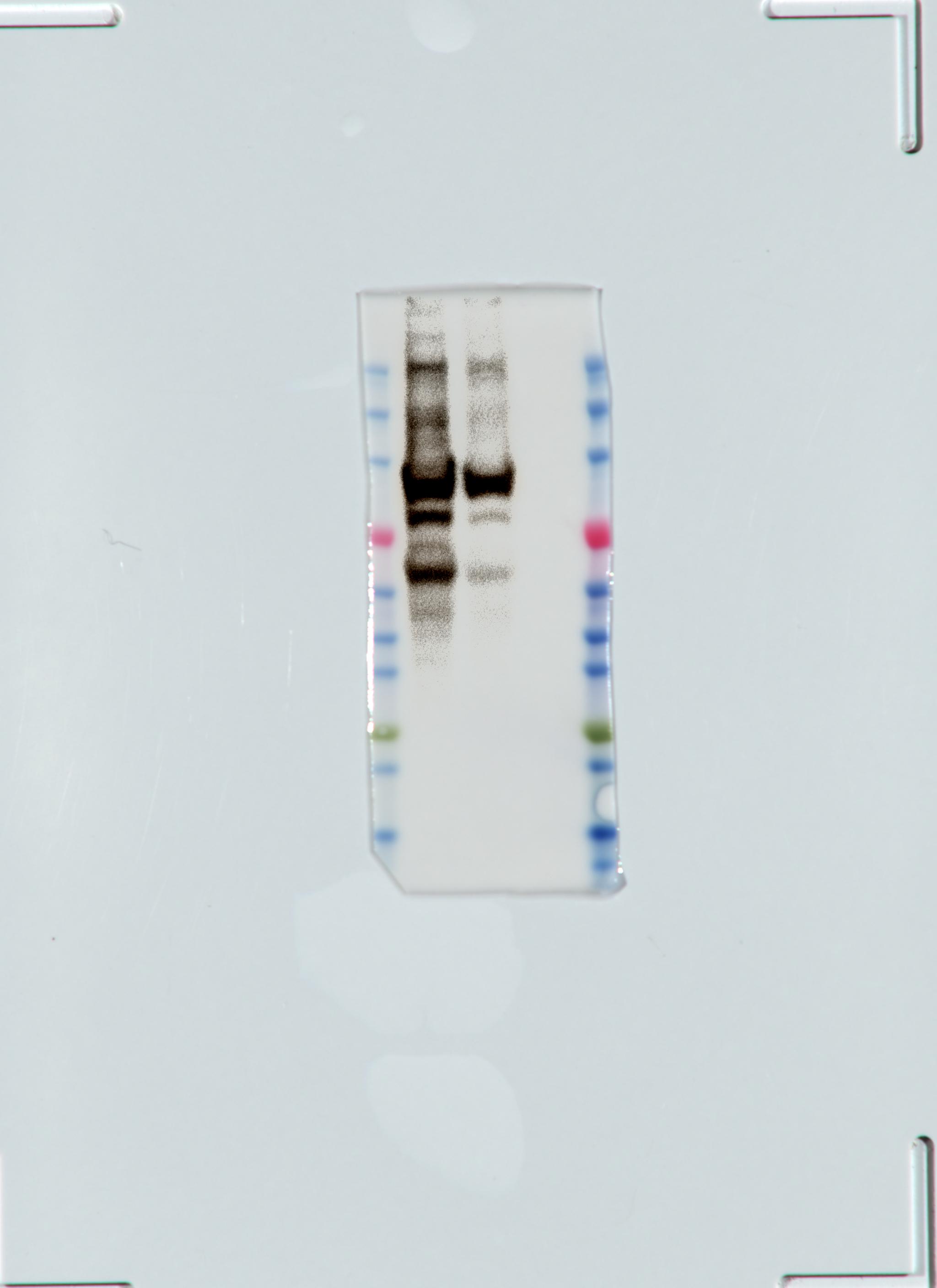

Supplement: Supplementary file 6 — Source data Fig. 4 [file 44318_2024_197_MOESM6_ESM.zip › SD figure 4/4E/4E replicate-3/lysate HSP-4/lysate HSP-4.tif]

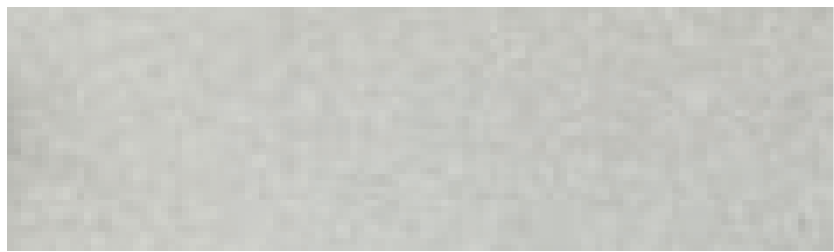

Supplement: Supplementary file 6 — Source data Fig. 4 [file 44318_2024_197_MOESM6_ESM.zip › SD figure 4/4E/4E replicate-4/IP FEM-3/IP FEM-3.tif]

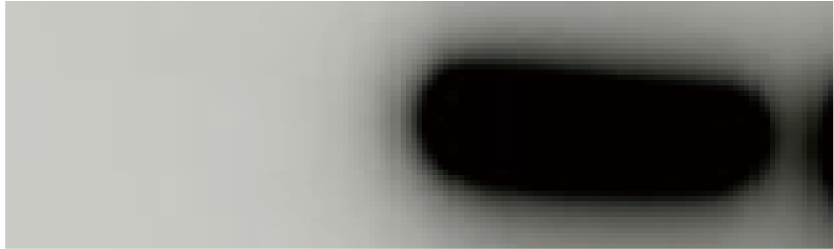

Supplement: Supplementary file 6 — Source data Fig. 4 [file 44318_2024_197_MOESM6_ESM.zip › SD figure 4/4E/4E replicate-4/IP HSP-4/IP HSP-4.tif]

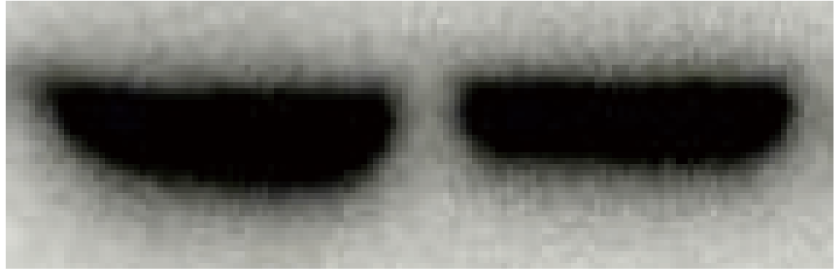

Supplement: Supplementary file 6 — Source data Fig. 4 [file 44318_2024_197_MOESM6_ESM.zip › SD figure 4/4E/4E replicate-4/lysate FEM-3/lysate FEM-3.tif]

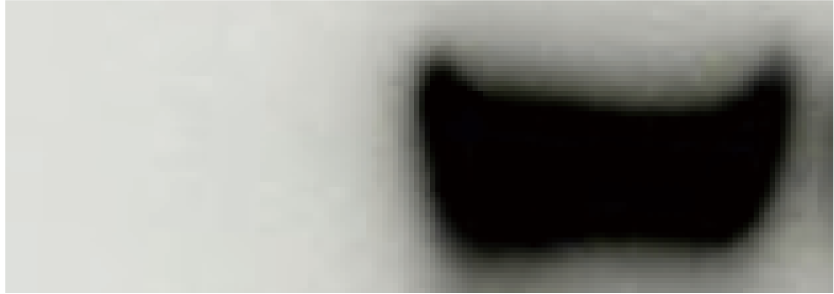

Supplement: Supplementary file 6 — Source data Fig. 4 [file 44318_2024_197_MOESM6_ESM.zip › SD figure 4/4E/4E replicate-4/lysate HSP-4/lysate HSP-4.tif]

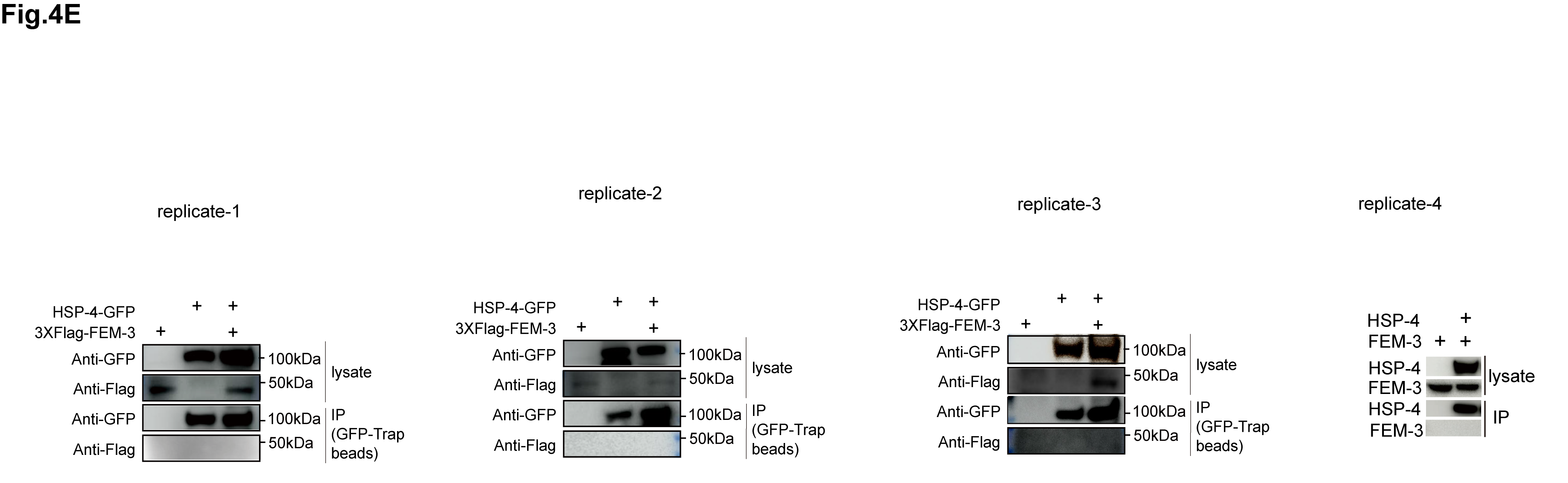

Supplement: Supplementary file 6 — Source data Fig. 4 [file 44318_2024_197_MOESM6_ESM.zip › SD figure 4/4E/4E.tif]

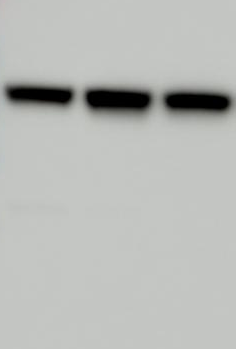

Supplement: Supplementary file 6 — Source data Fig. 4 [file 44318_2024_197_MOESM6_ESM.zip › SD figure 4/4F/4F replicate-1/beta-actin/beta-actin.tif]

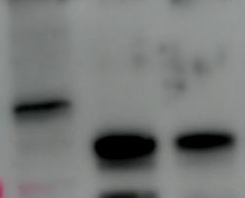

Supplement: Supplementary file 6 — Source data Fig. 4 [file 44318_2024_197_MOESM6_ESM.zip › SD figure 4/4F/4F replicate-1/TRA-2/TRA-2.tif]

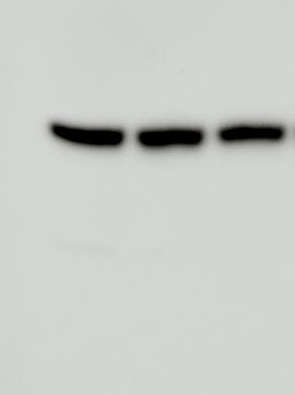

Supplement: Supplementary file 6 — Source data Fig. 4 [file 44318_2024_197_MOESM6_ESM.zip › SD figure 4/4F/4F replicate-2/beta-actin/beta-actin.tif]

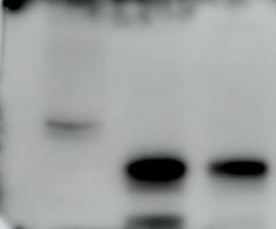

Supplement: Supplementary file 6 — Source data Fig. 4 [file 44318_2024_197_MOESM6_ESM.zip › SD figure 4/4F/4F replicate-2/TRA-2/TRA-2.tif]

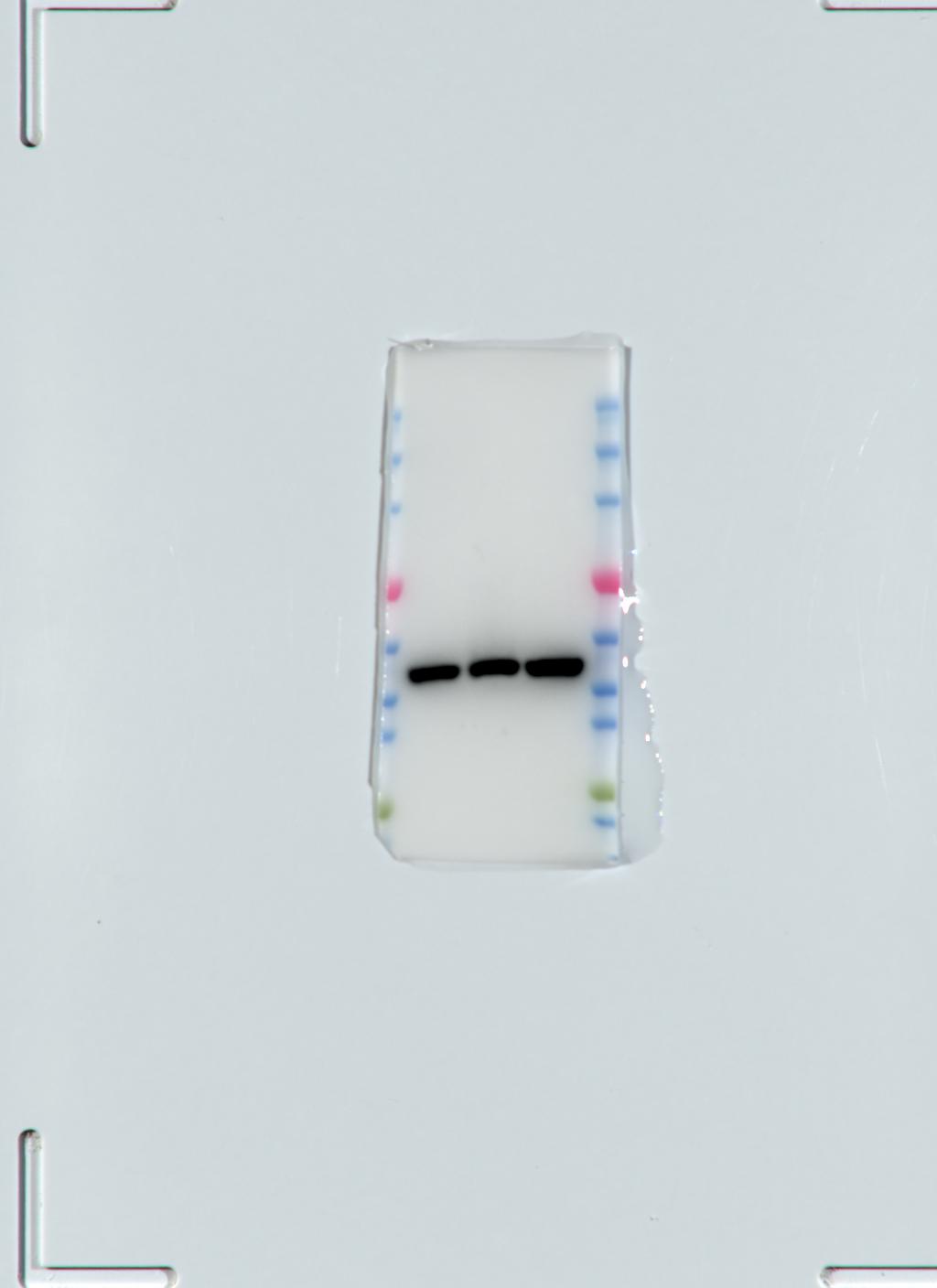

Supplement: Supplementary file 6 — Source data Fig. 4 [file 44318_2024_197_MOESM6_ESM.zip › SD figure 4/4F/4F replicate-3/beta-actin/beta-actin.tif]

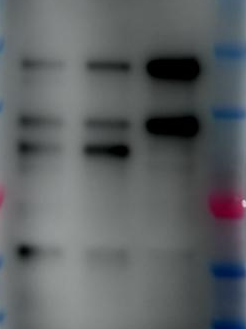

Supplement: Supplementary file 6 — Source data Fig. 4 [file 44318_2024_197_MOESM6_ESM.zip › SD figure 4/4F/4F replicate-3/TRA-2/TRA-2.tif]

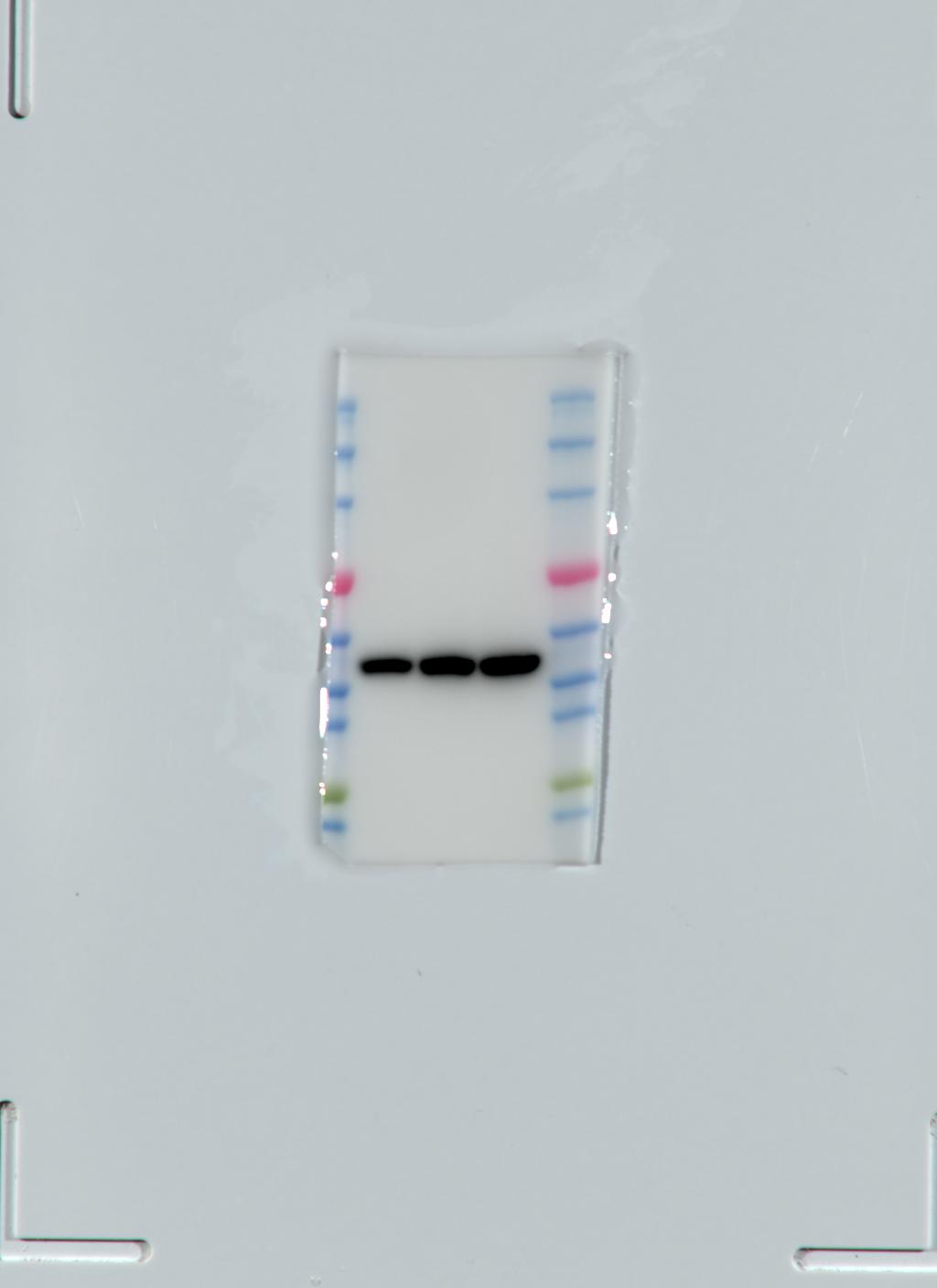

Supplement: Supplementary file 6 — Source data Fig. 4 [file 44318_2024_197_MOESM6_ESM.zip › SD figure 4/4F/4F replicate-4/beta-actin/bta-actin.tif]

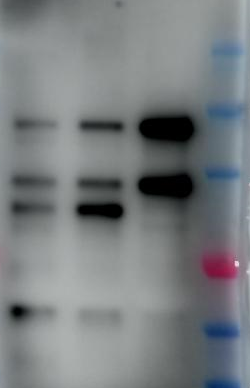

Supplement: Supplementary file 6 — Source data Fig. 4 [file 44318_2024_197_MOESM6_ESM.zip › SD figure 4/4F/4F replicate-4/TRA-2/TRA-2.tif]

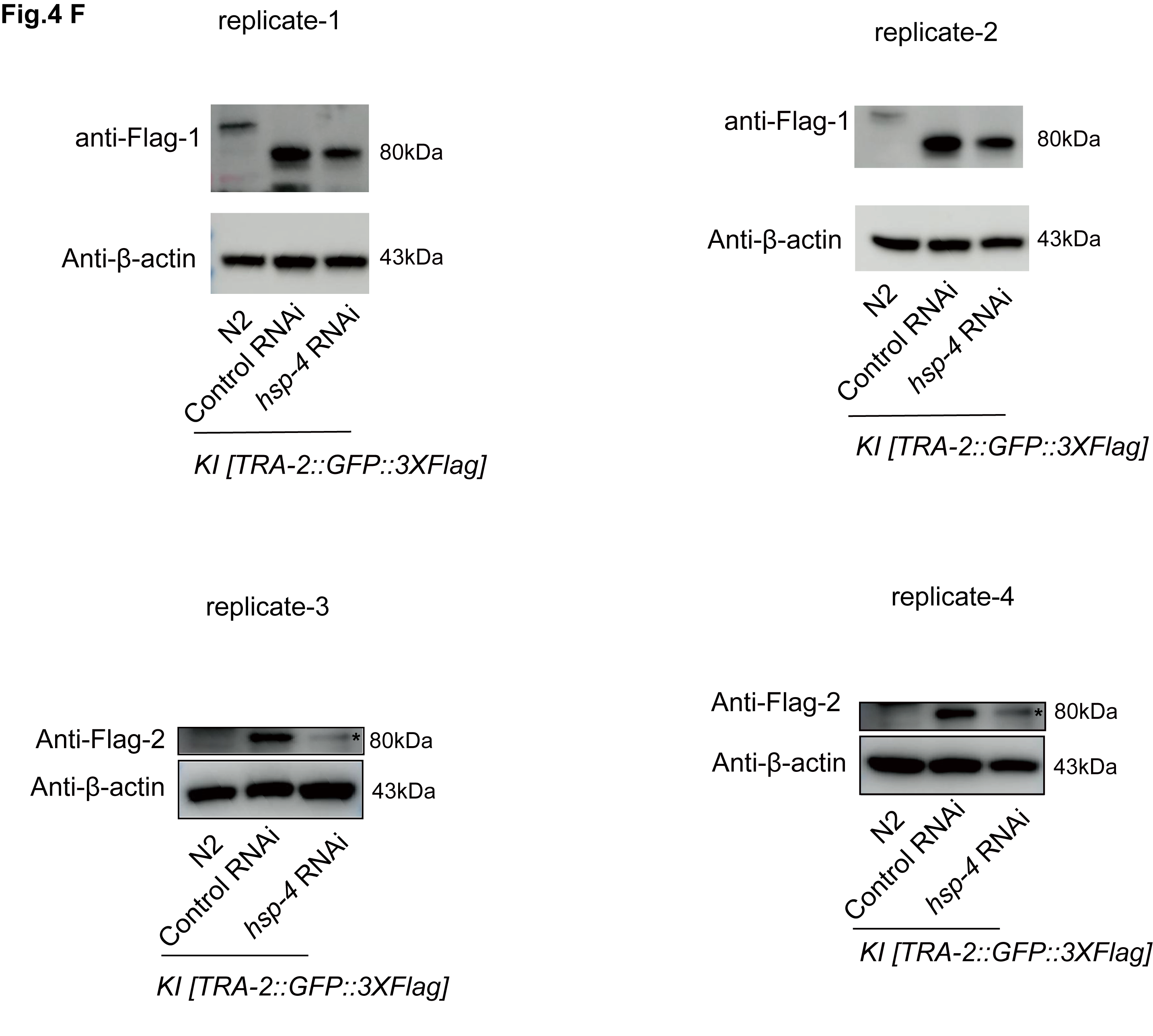

Supplement: Supplementary file 6 — Source data Fig. 4 [file 44318_2024_197_MOESM6_ESM.zip › SD figure 4/4F/4F.tif]

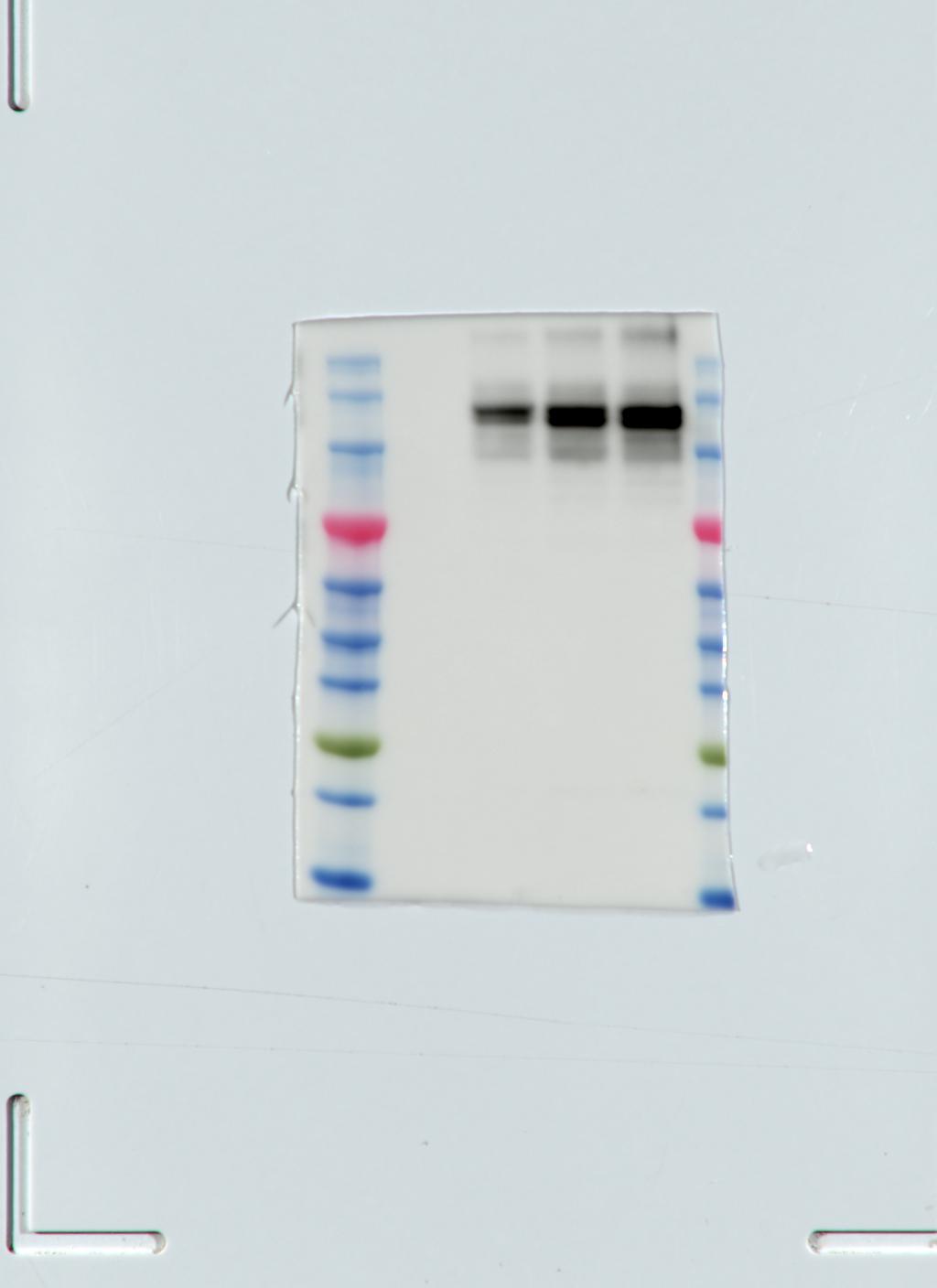

Supplement: Supplementary file 7 — Source data Fig. 5 [file 44318_2024_197_MOESM7_ESM.zip › SD figure 5/5C/5C replicate-1/IP P97/IP P97.tif]

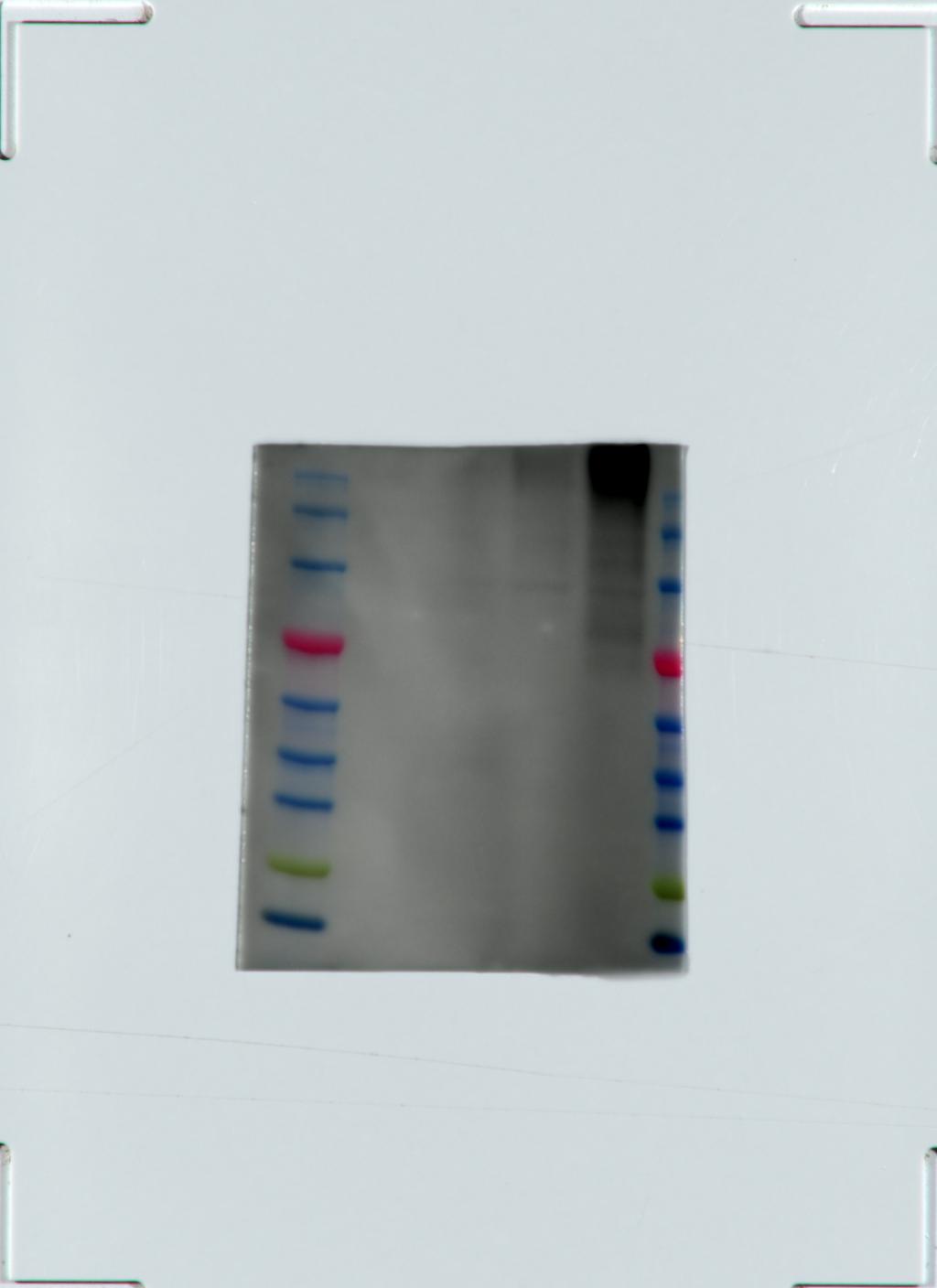

Supplement: Supplementary file 7 — Source data Fig. 5 [file 44318_2024_197_MOESM7_ESM.zip › SD figure 5/5C/5C replicate-1/IP TRA-2/IP TRA-2.tif]

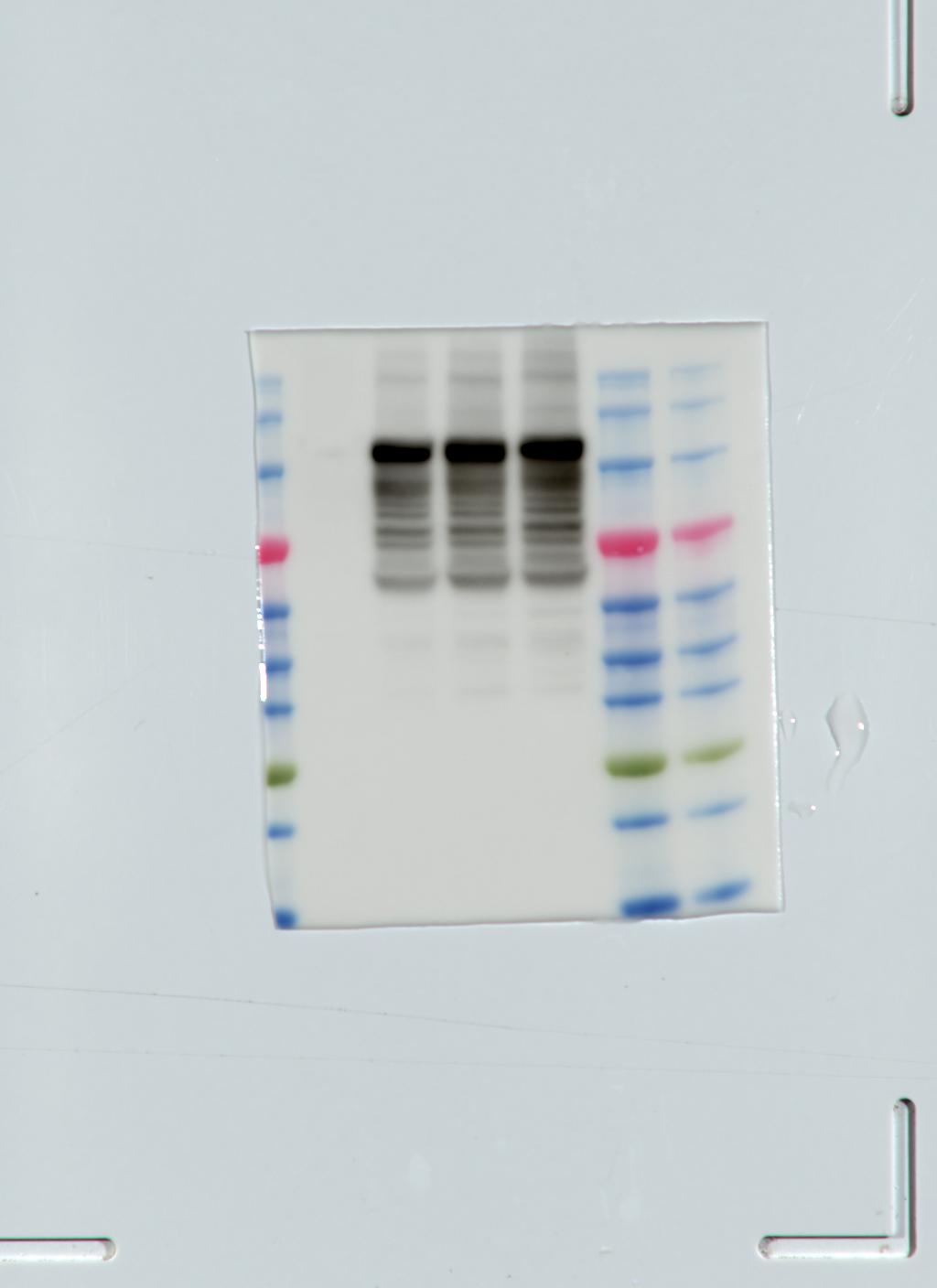

Supplement: Supplementary file 7 — Source data Fig. 5 [file 44318_2024_197_MOESM7_ESM.zip › SD figure 5/5C/5C replicate-1/lysate P97/lysate P97.tif]

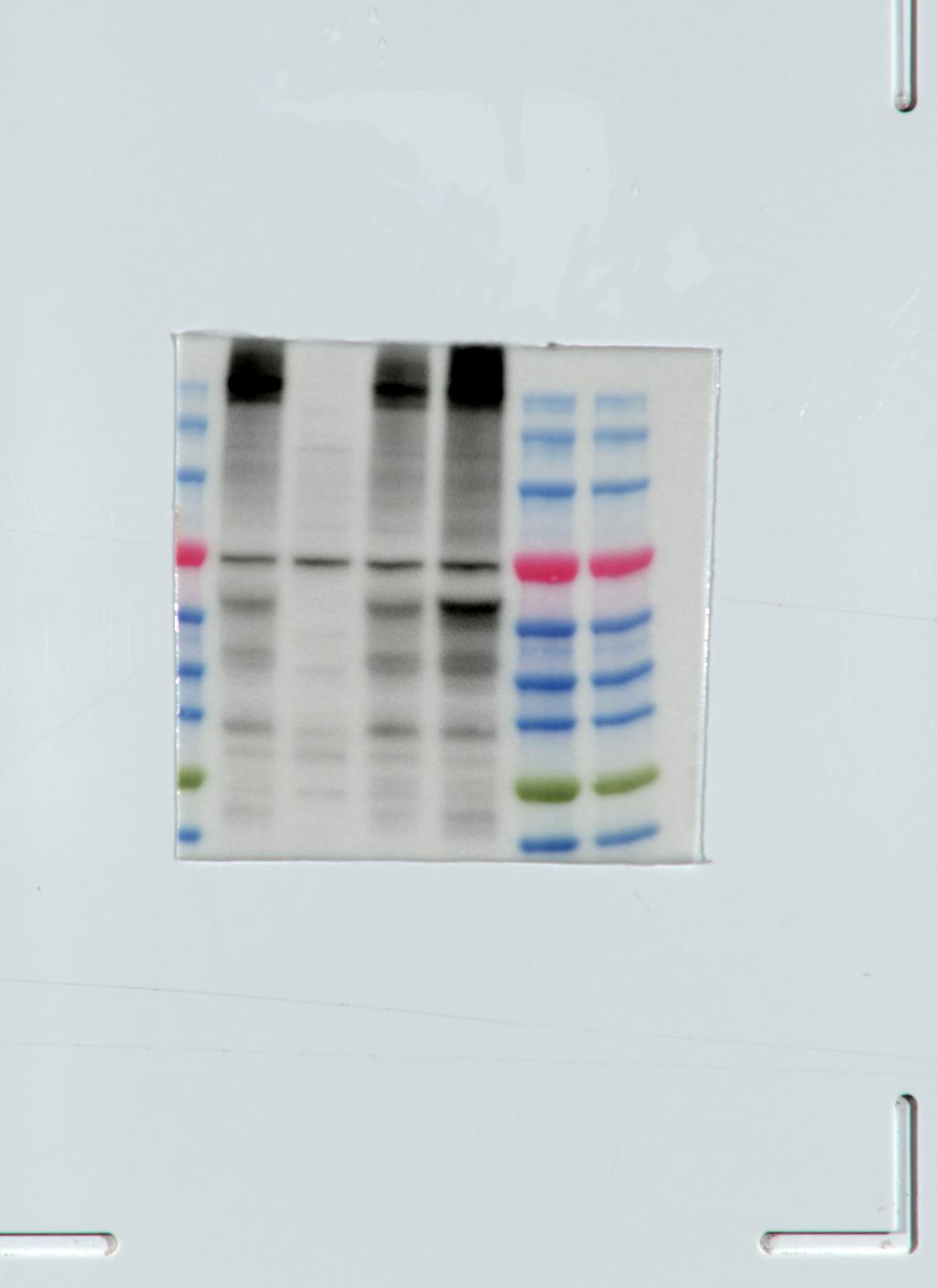

Supplement: Supplementary file 7 — Source data Fig. 5 [file 44318_2024_197_MOESM7_ESM.zip › SD figure 5/5C/5C replicate-1/lysate TRA-2/lysate TRA-2.tif]

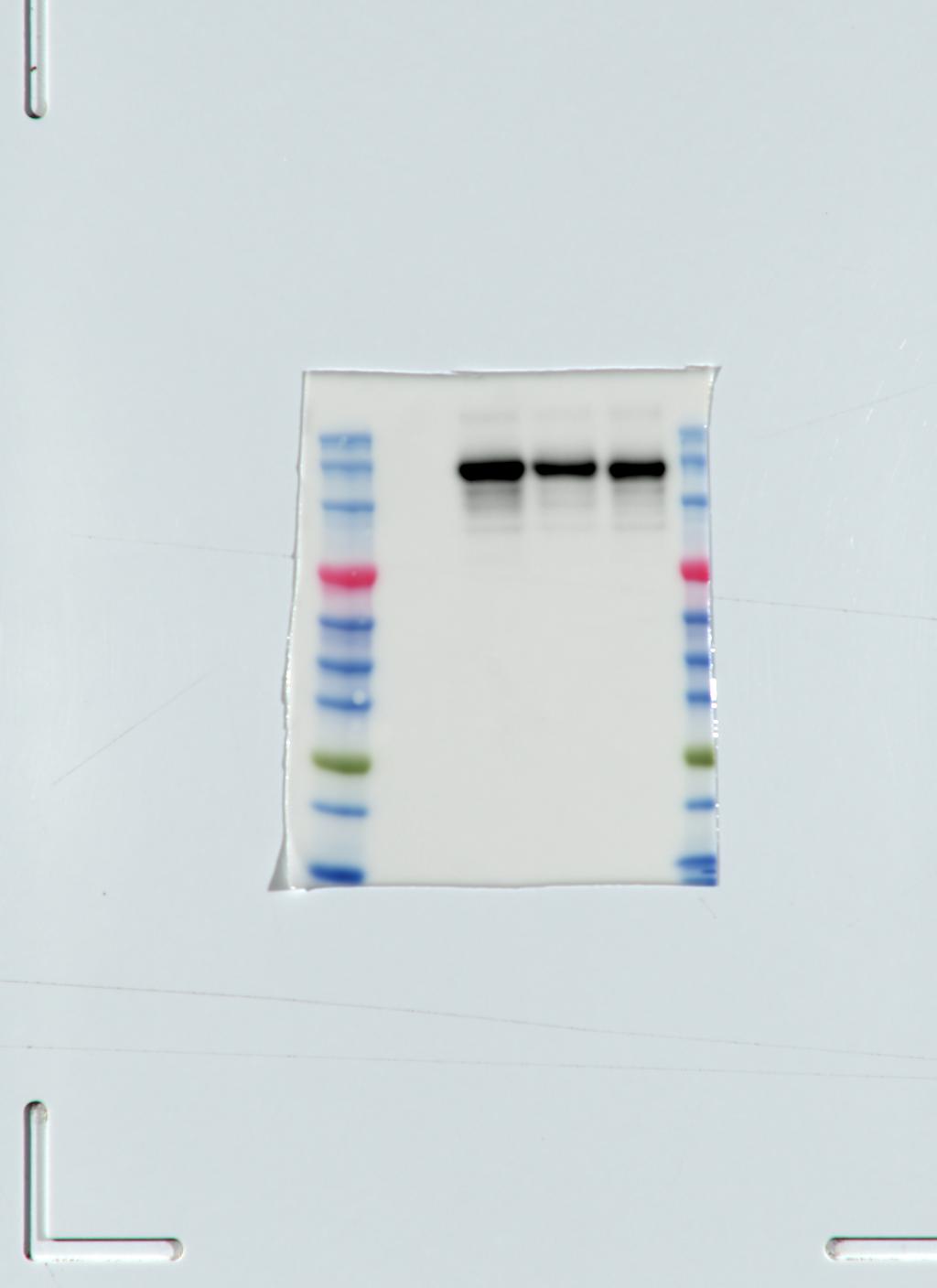

Supplement: Supplementary file 7 — Source data Fig. 5 [file 44318_2024_197_MOESM7_ESM.zip › SD figure 5/5C/5C replicate-2/IP P97/IP P97.tif]

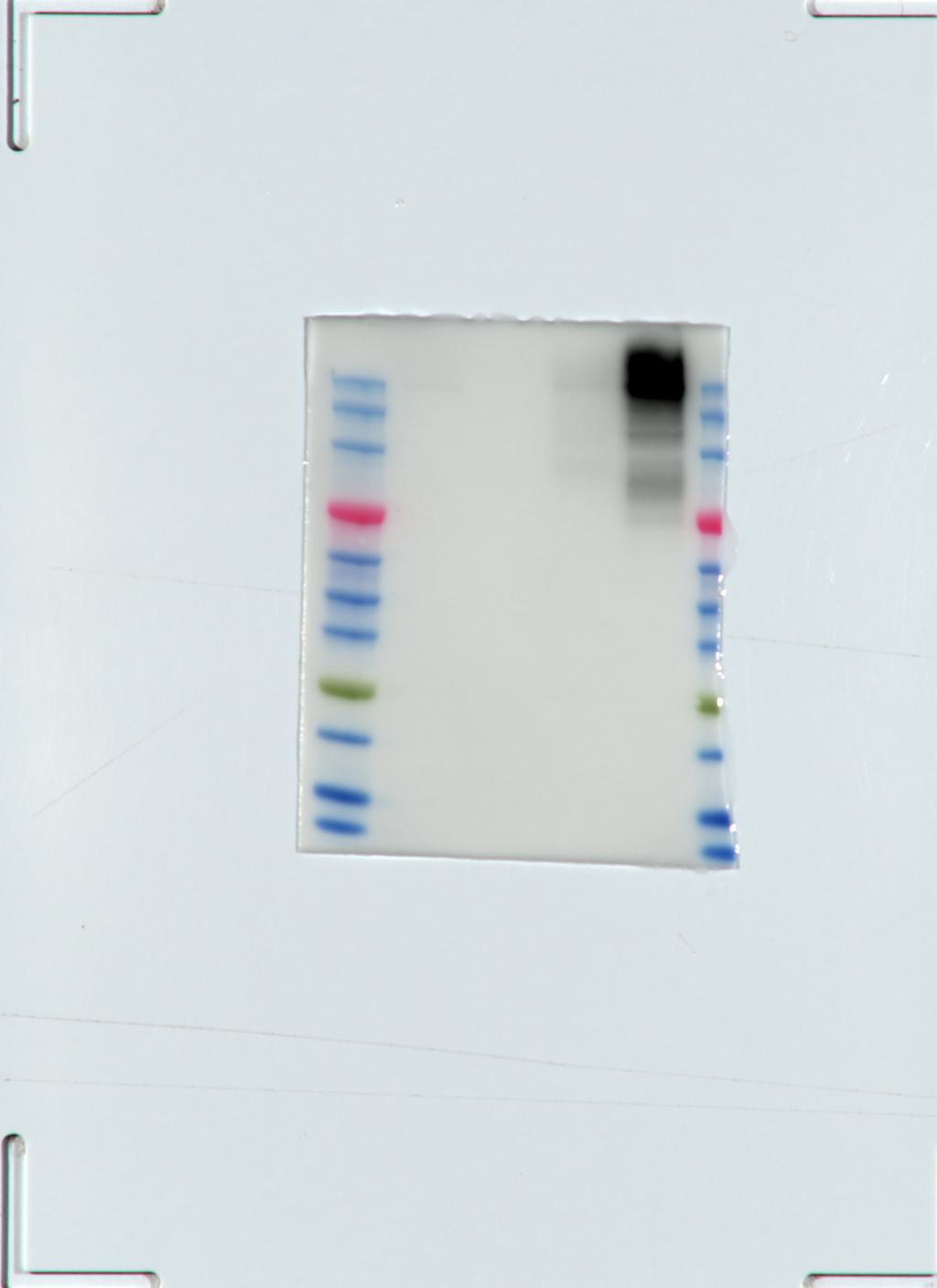

Supplement: Supplementary file 7 — Source data Fig. 5 [file 44318_2024_197_MOESM7_ESM.zip › SD figure 5/5C/5C replicate-2/IP TRA-2/IP TRA-2.tif]

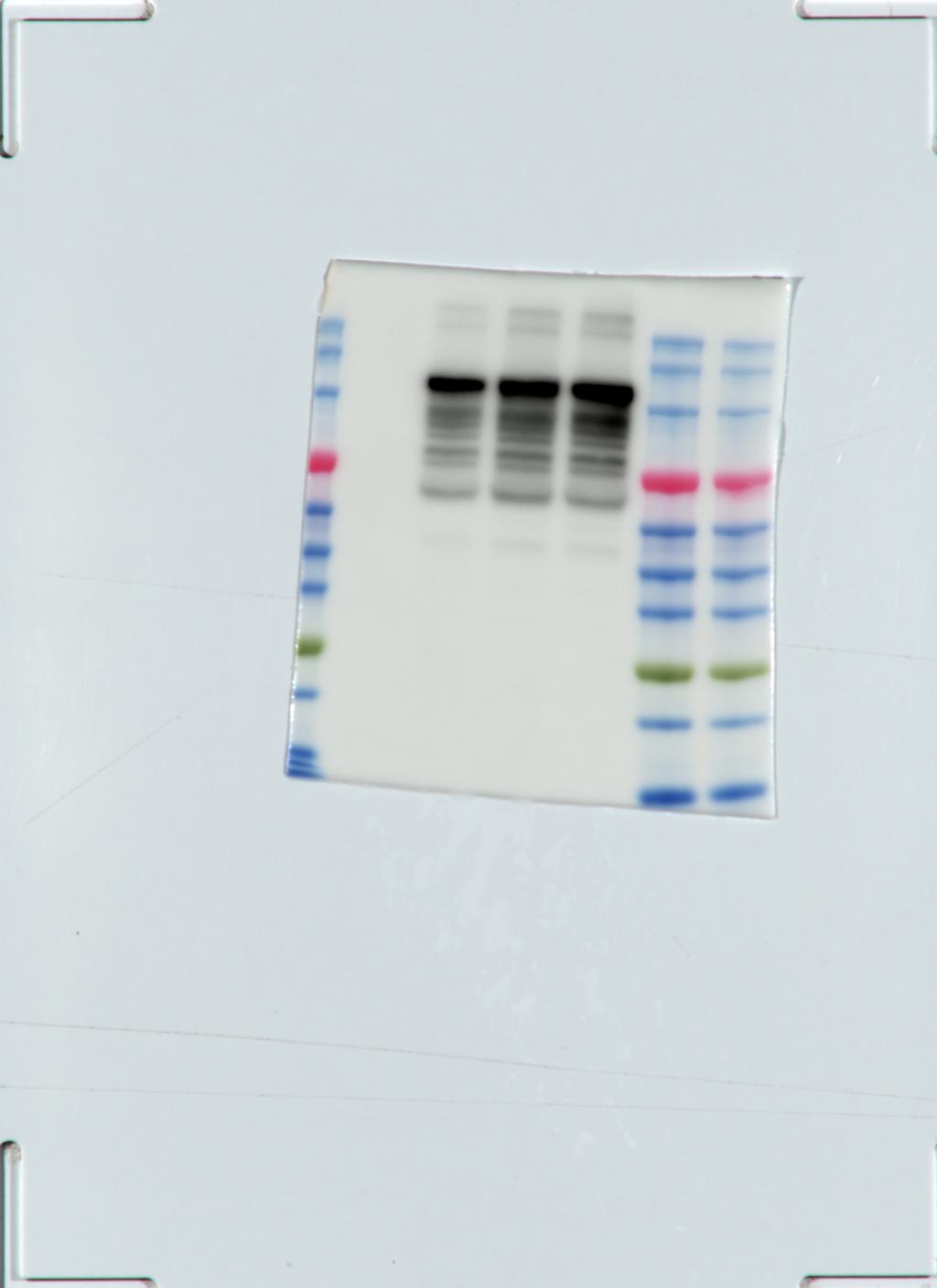

Supplement: Supplementary file 7 — Source data Fig. 5 [file 44318_2024_197_MOESM7_ESM.zip › SD figure 5/5C/5C replicate-2/lysate P97/lysate P97.tif]

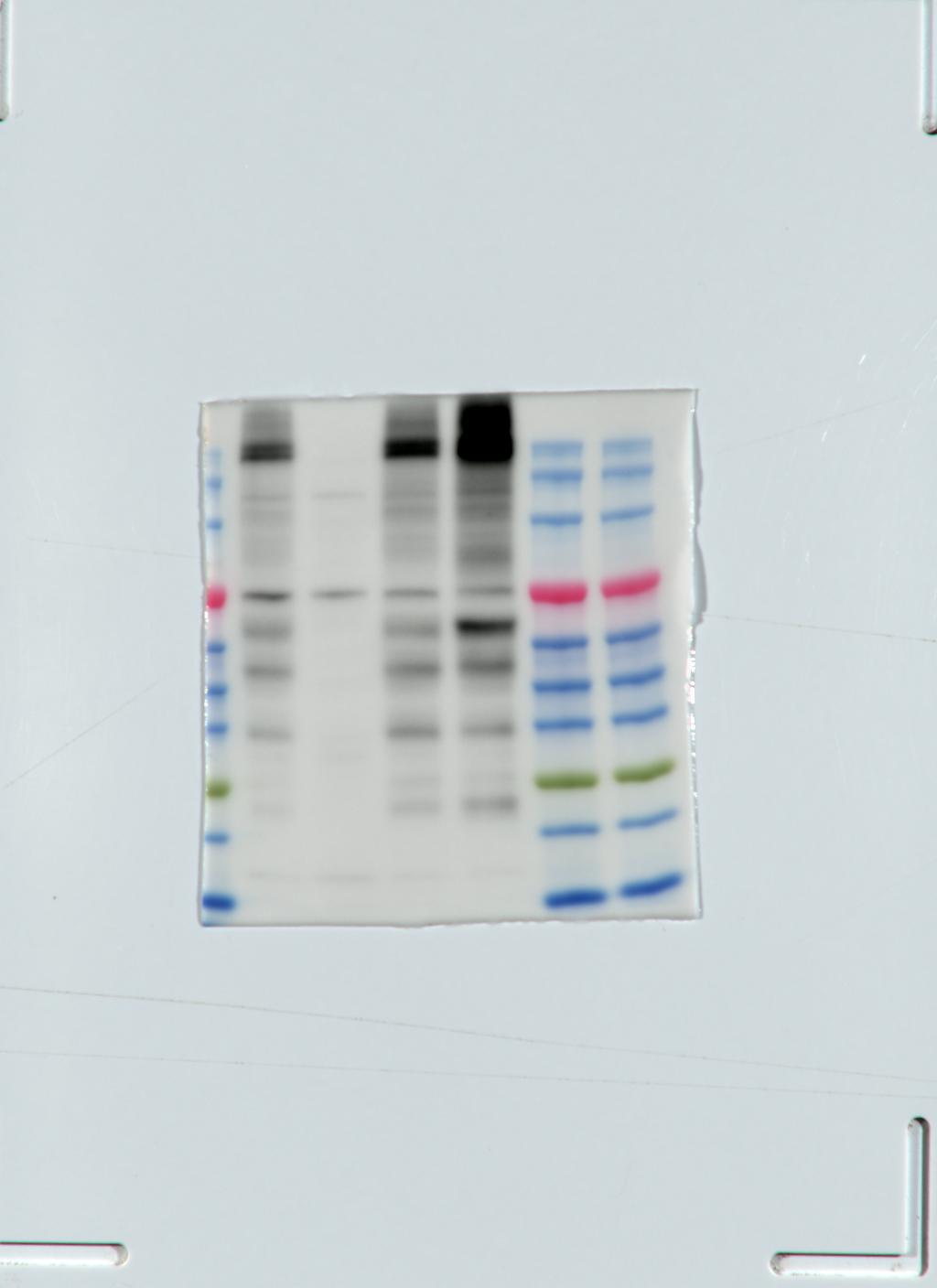

Supplement: Supplementary file 7 — Source data Fig. 5 [file 44318_2024_197_MOESM7_ESM.zip › SD figure 5/5C/5C replicate-2/lysate TRA-2/lysate TRA-2.tif]

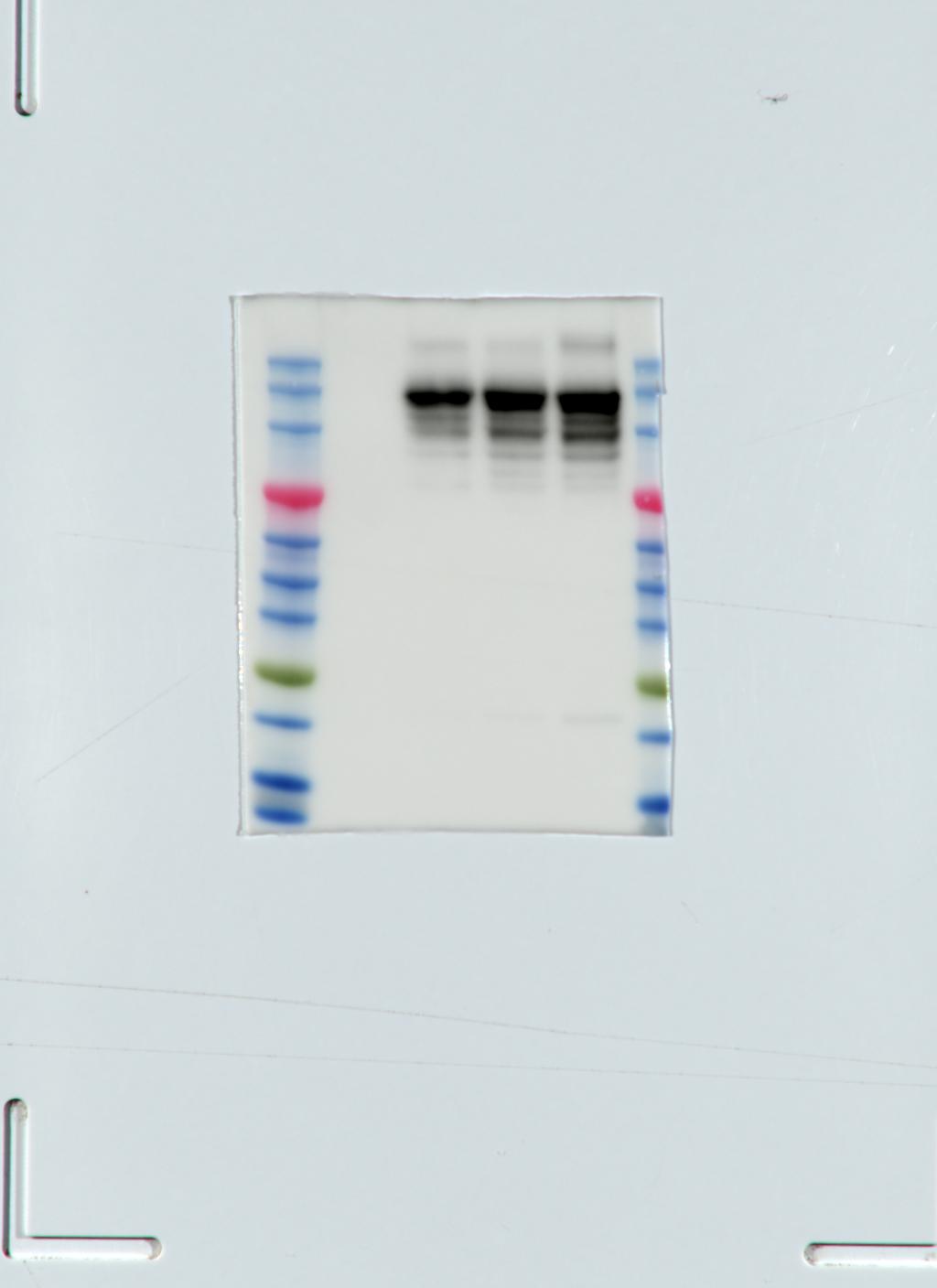

Supplement: Supplementary file 7 — Source data Fig. 5 [file 44318_2024_197_MOESM7_ESM.zip › SD figure 5/5C/5C replicate-3/IP P97/IP P97.tif]

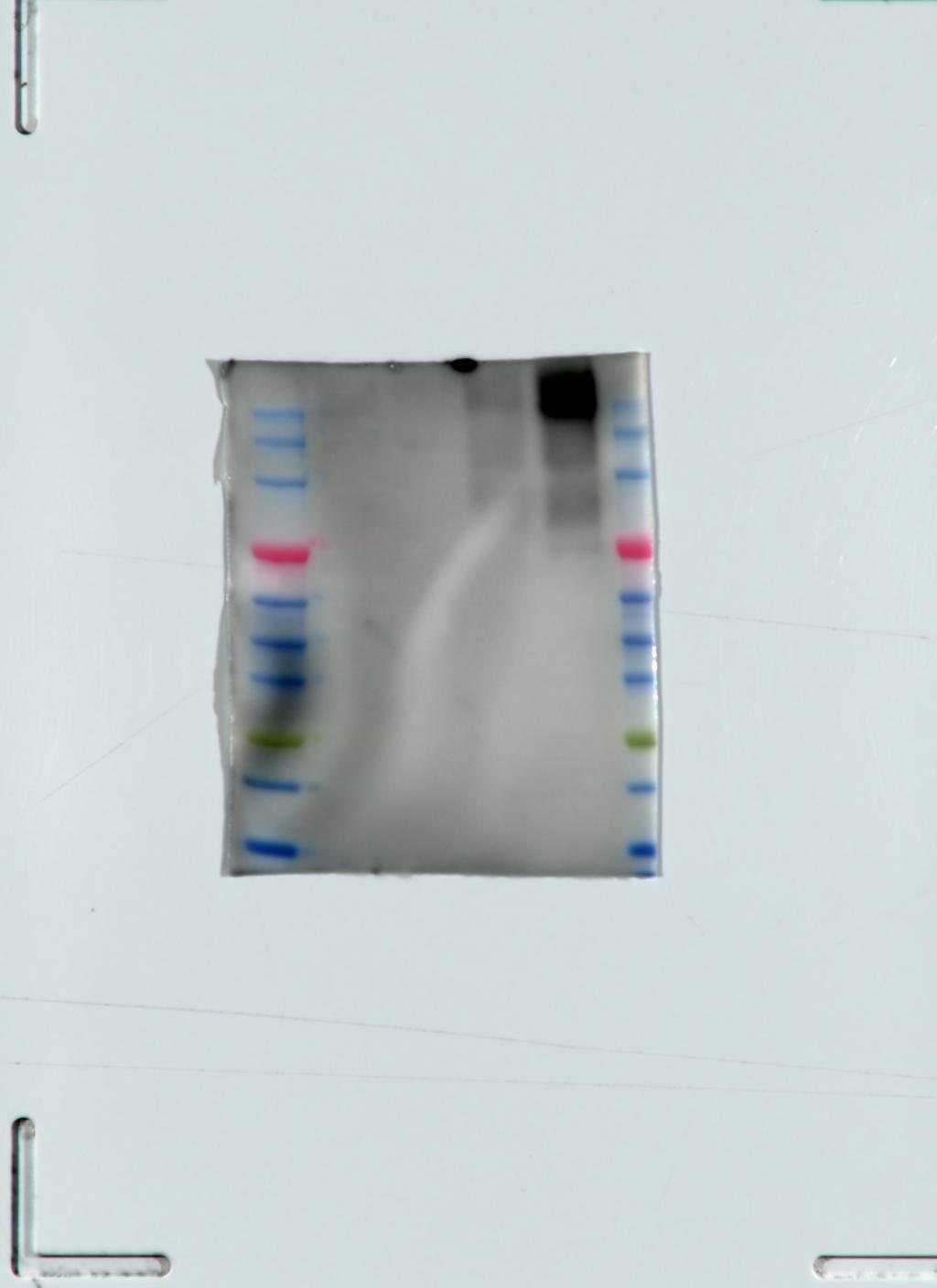

Supplement: Supplementary file 7 — Source data Fig. 5 [file 44318_2024_197_MOESM7_ESM.zip › SD figure 5/5C/5C replicate-3/IP TRA-2/IP TRA-2.tif]

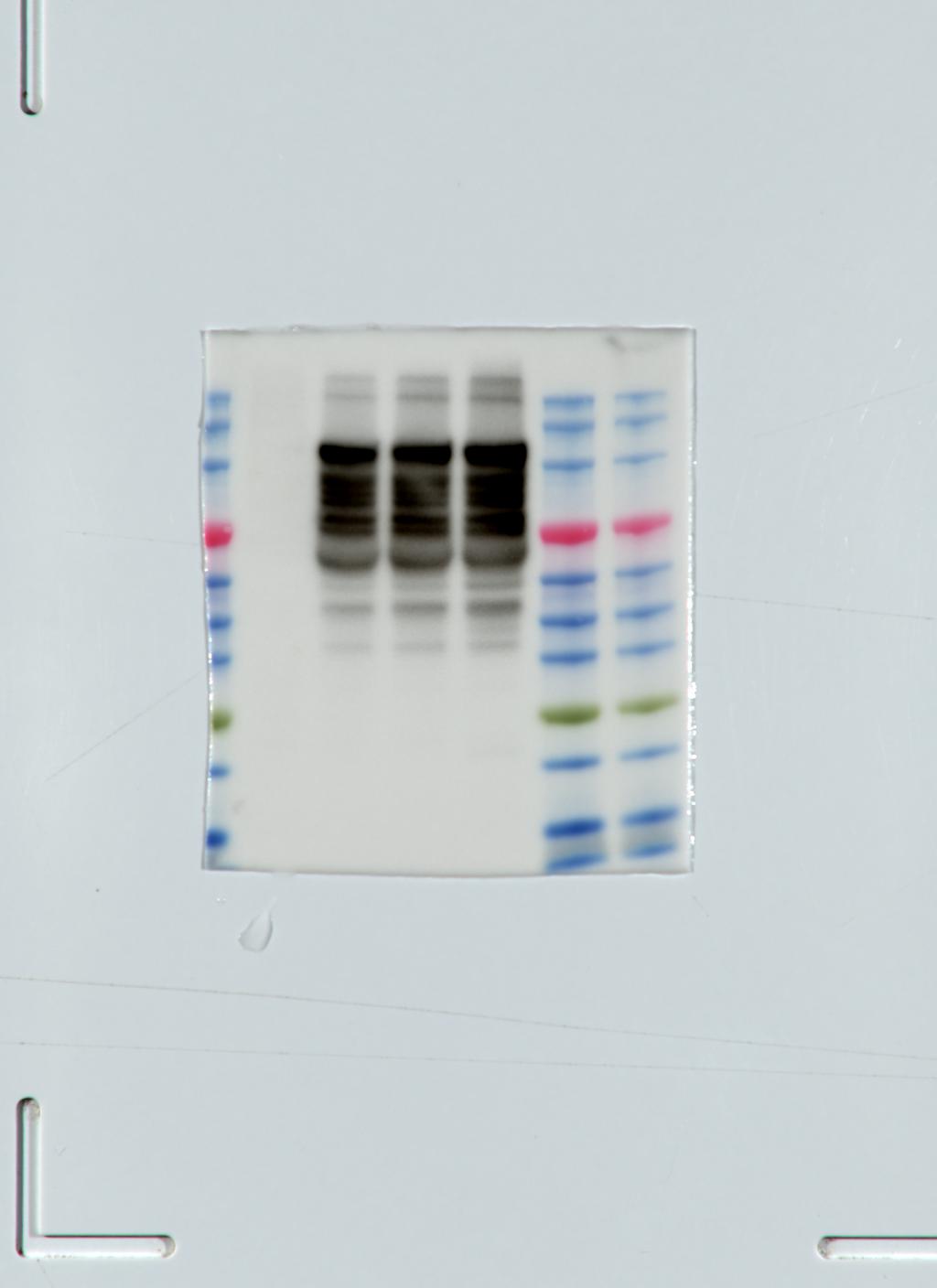

Supplement: Supplementary file 7 — Source data Fig. 5 [file 44318_2024_197_MOESM7_ESM.zip › SD figure 5/5C/5C replicate-3/lysate P97/lysate P97.tif]

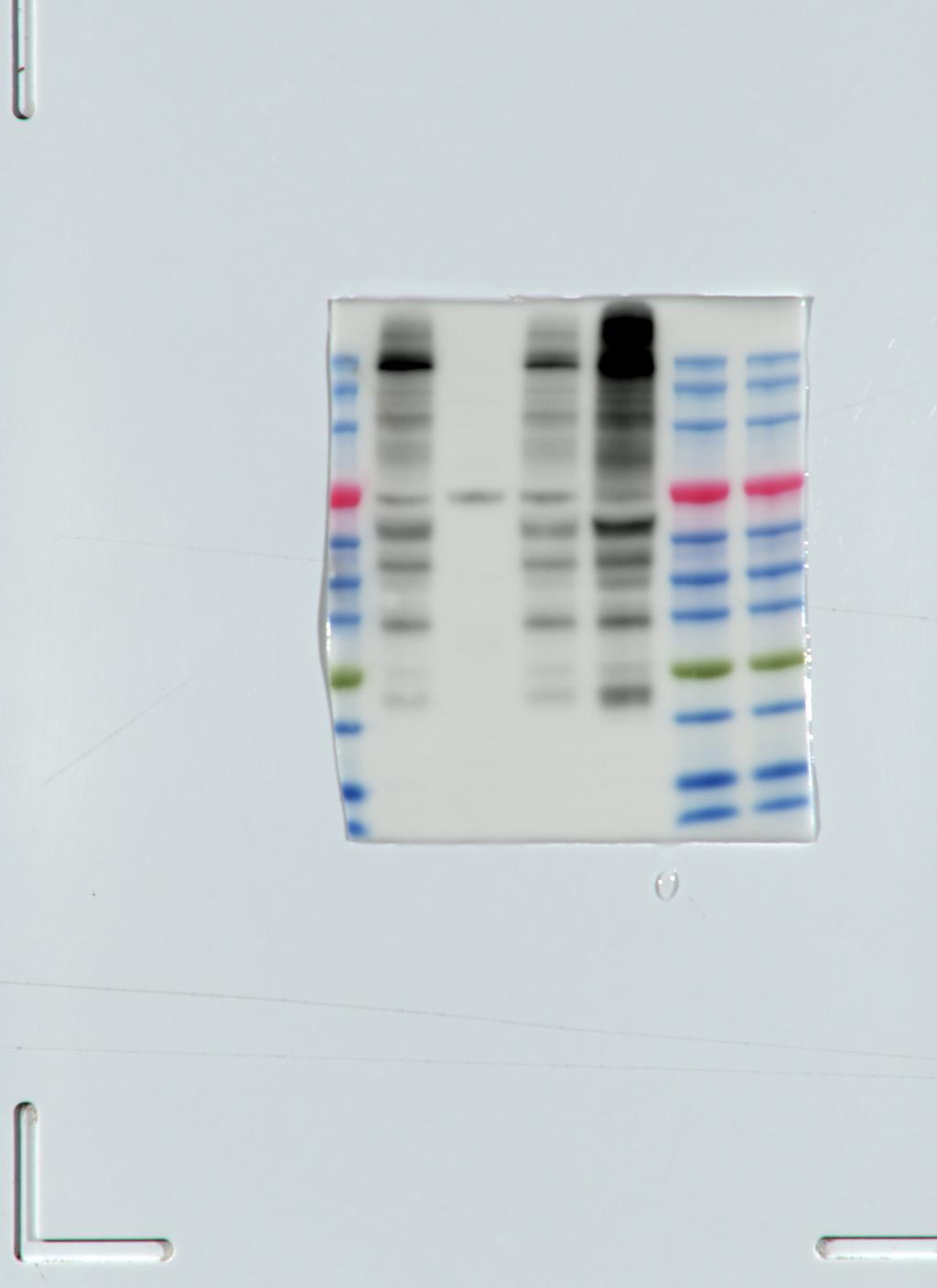

Supplement: Supplementary file 7 — Source data Fig. 5 [file 44318_2024_197_MOESM7_ESM.zip › SD figure 5/5C/5C replicate-3/lysate TRA-2/lysate TRA-2.tif]

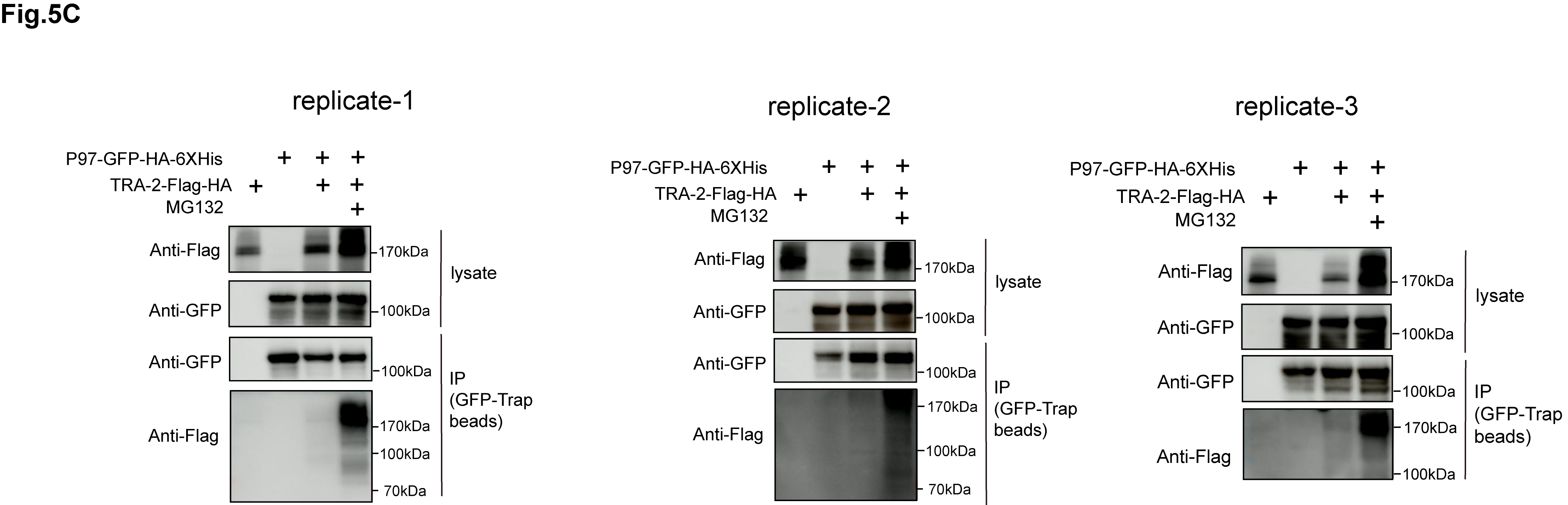

Supplement: Supplementary file 7 — Source data Fig. 5 [file 44318_2024_197_MOESM7_ESM.zip › SD figure 5/5C/5C.tif]

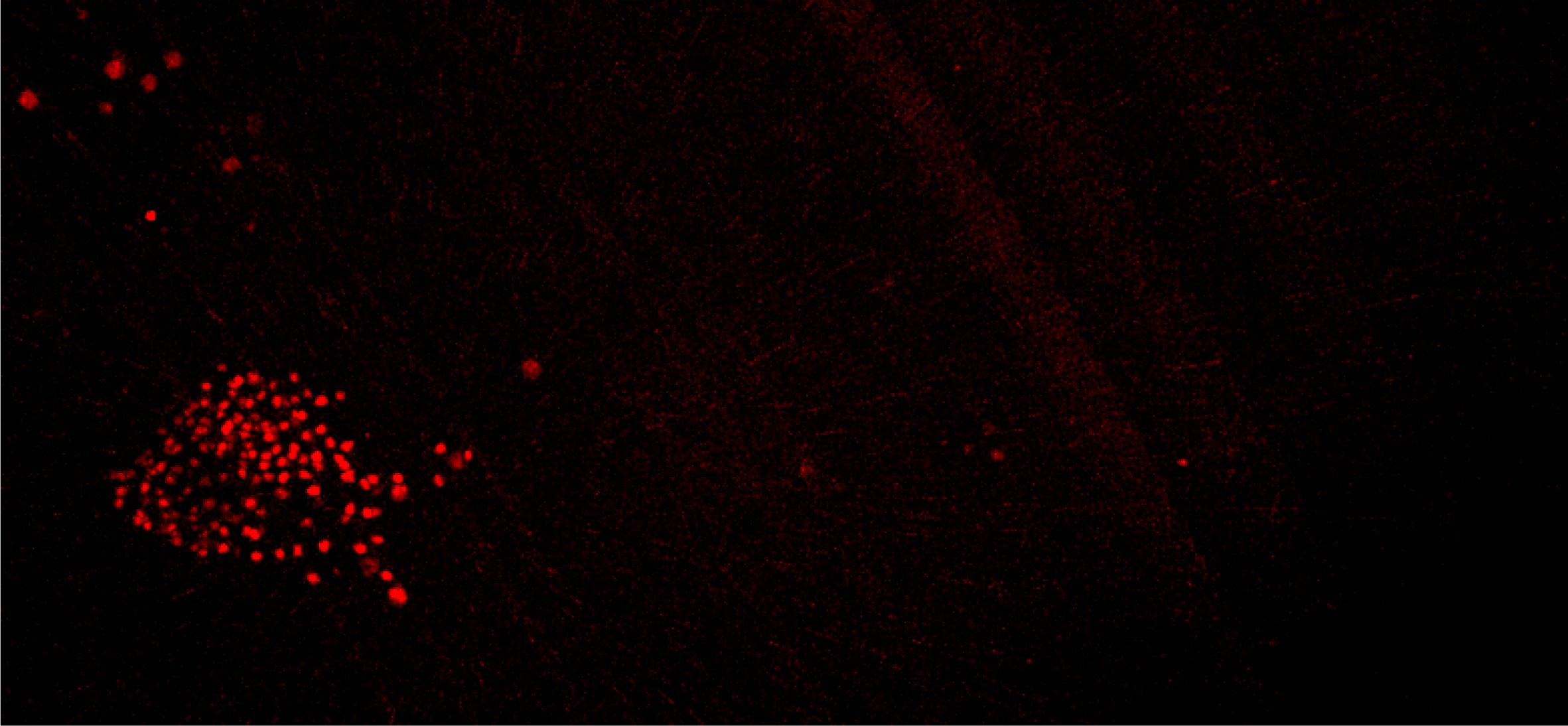

Supplement: Supplementary file 8 — Source data Fig. 6 [file 44318_2024_197_MOESM8_ESM.zip › SD figure 6/6A/6A.tif]

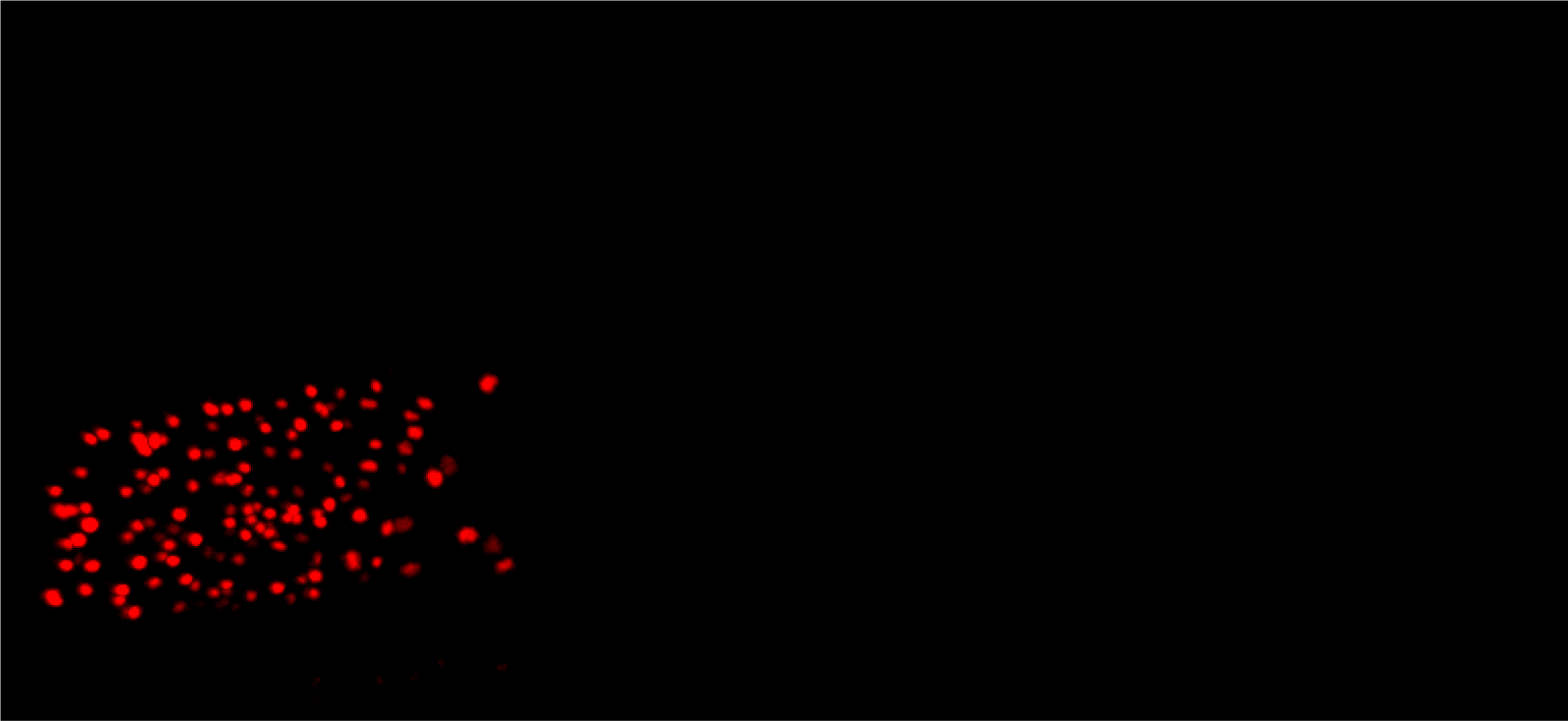

Supplement: Supplementary file 8 — Source data Fig. 6 [file 44318_2024_197_MOESM8_ESM.zip › SD figure 6/6B/6B.tif]

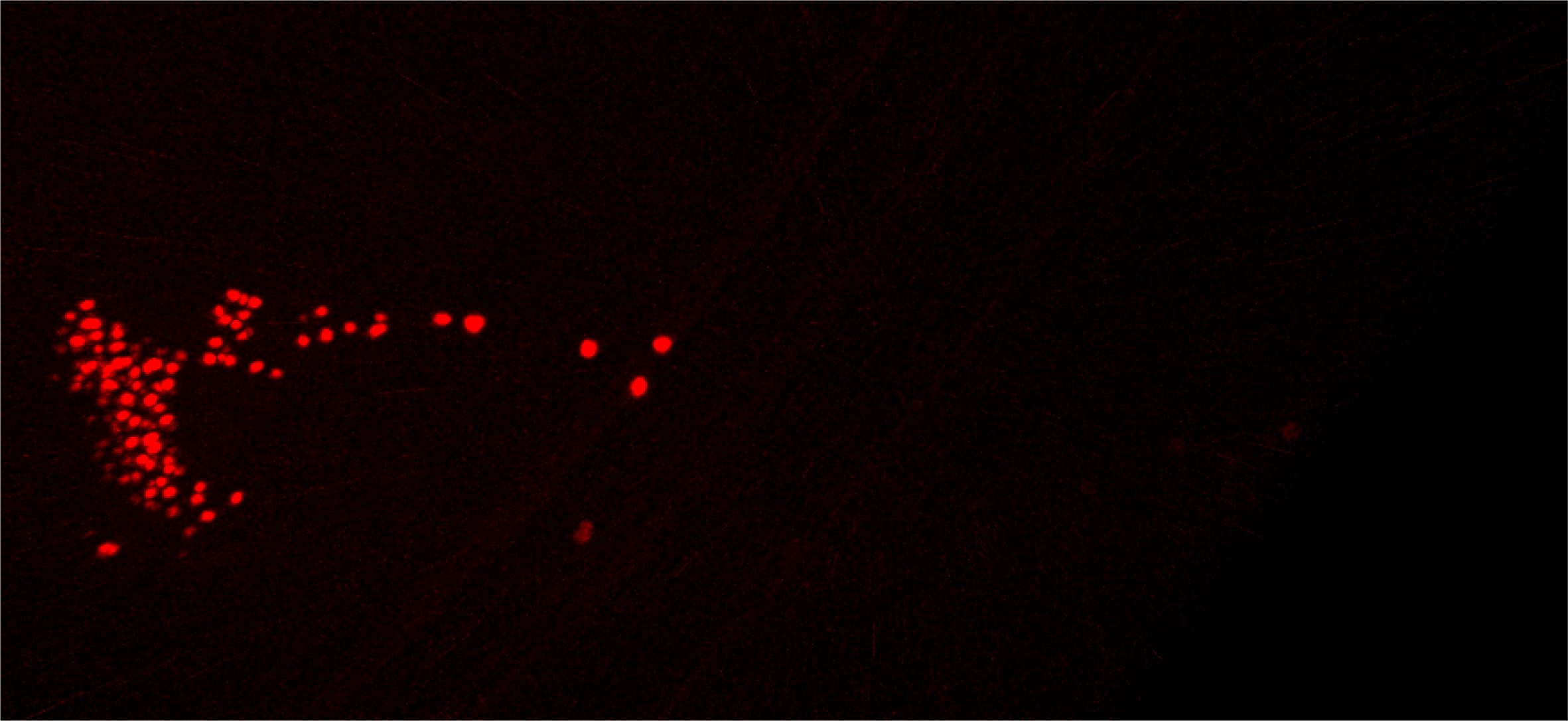

Supplement: Supplementary file 8 — Source data Fig. 6 [file 44318_2024_197_MOESM8_ESM.zip › SD figure 6/6C/6C.tif]

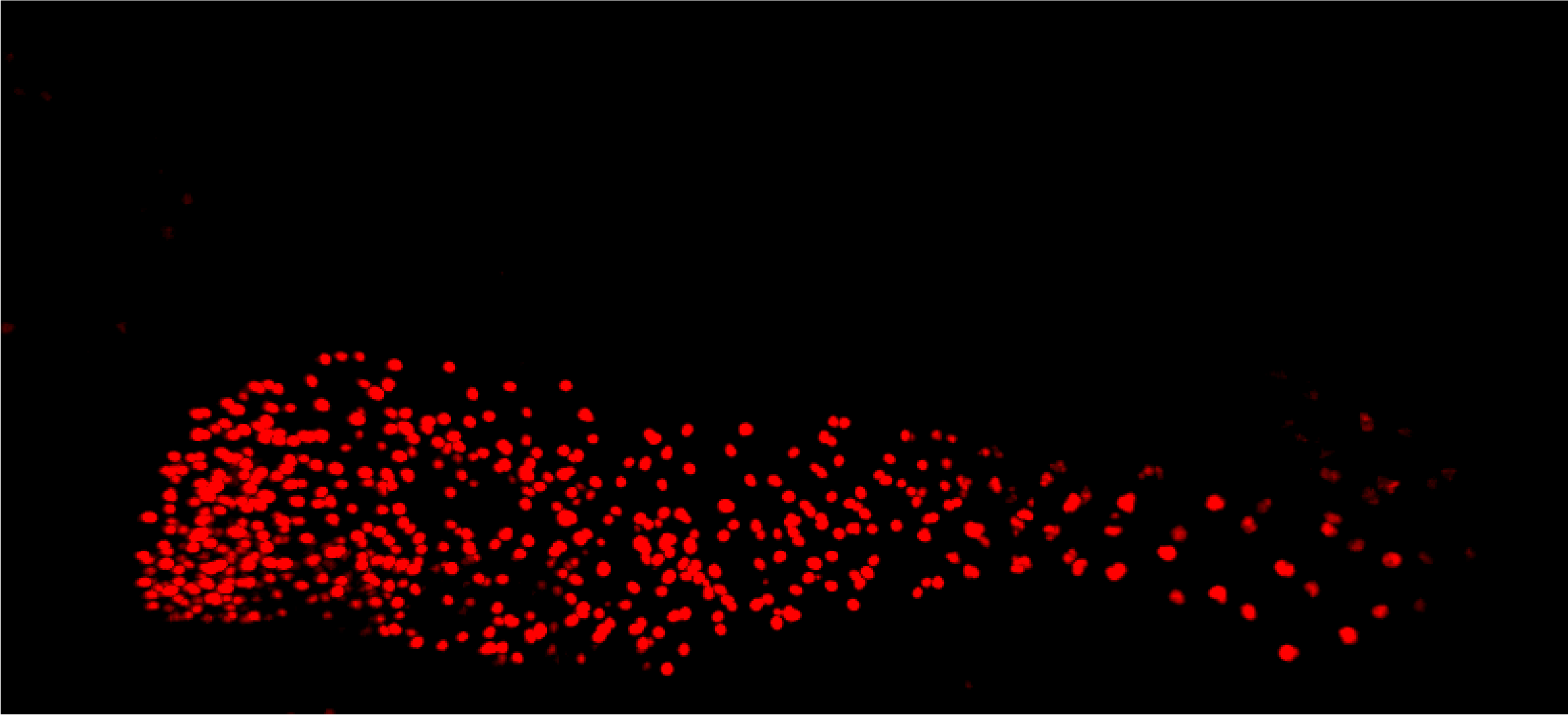

Supplement: Supplementary file 8 — Source data Fig. 6 [file 44318_2024_197_MOESM8_ESM.zip › SD figure 6/6D/6D.tif]

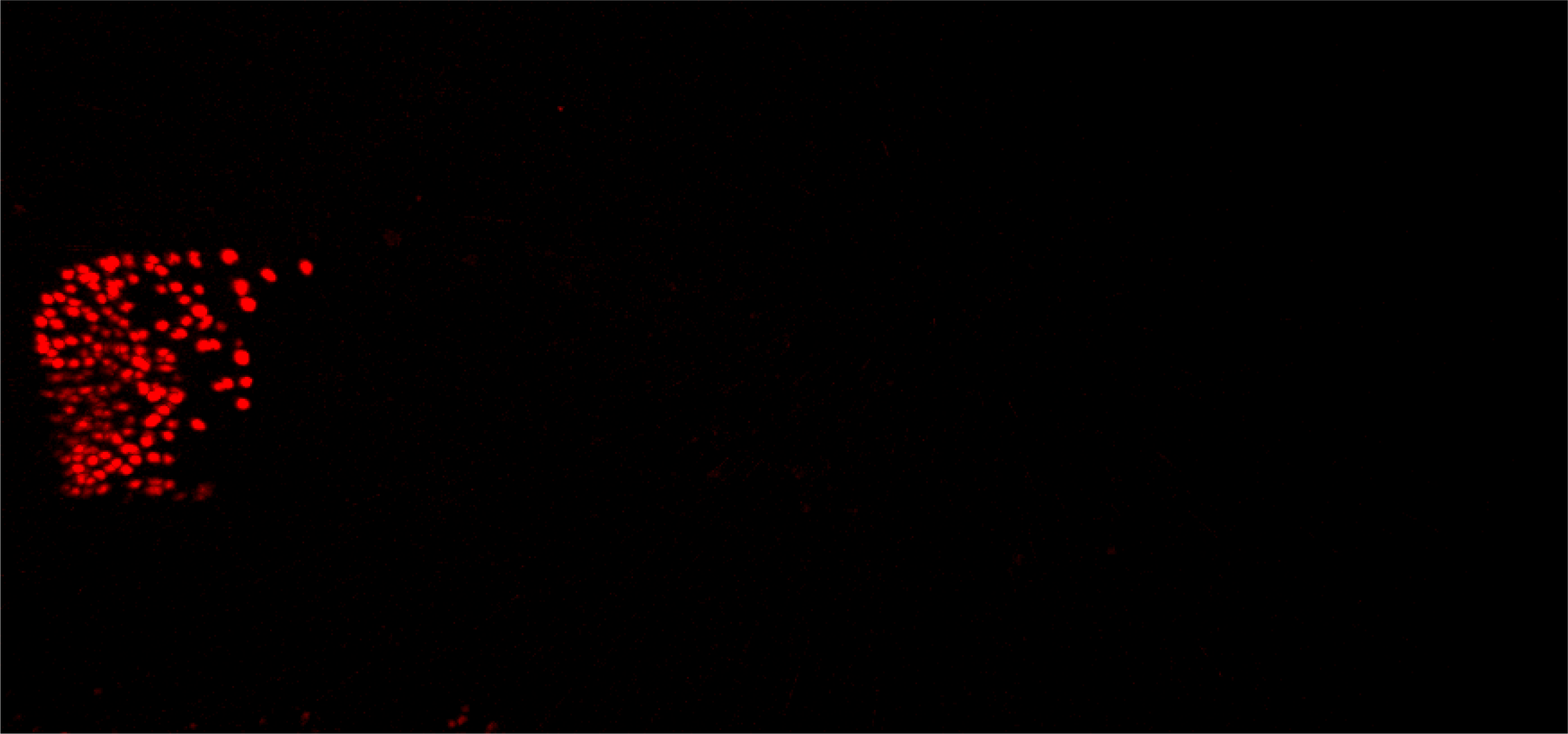

Supplement: Supplementary file 8 — Source data Fig. 6 [file 44318_2024_197_MOESM8_ESM.zip › SD figure 6/6E/6E.tif]

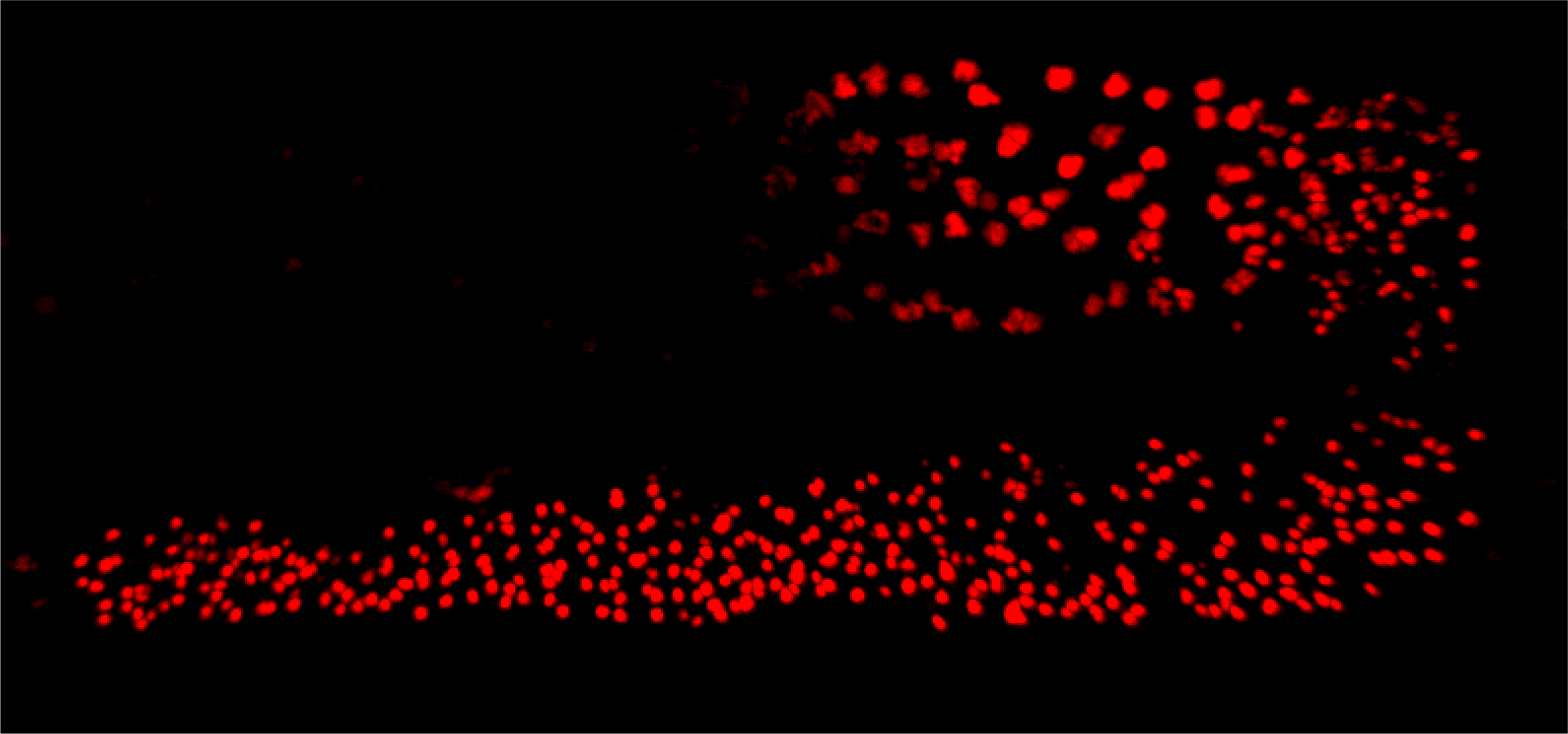

Supplement: Supplementary file 8 — Source data Fig. 6 [file 44318_2024_197_MOESM8_ESM.zip › SD figure 6/6F/6F.tif]
