# Supplementary figures and images for: Identification of BiP as a temperature sensor mediating temperature-induced germline sex reversal in C. elegans (part 2 of 2)
Source: EMBO J. 2024 Aug 12;43(18):4020–48. doi: 10.1038/s44318-024-00197-z (PMC11405683; doi:10.1038/s44318-024-00197-z)

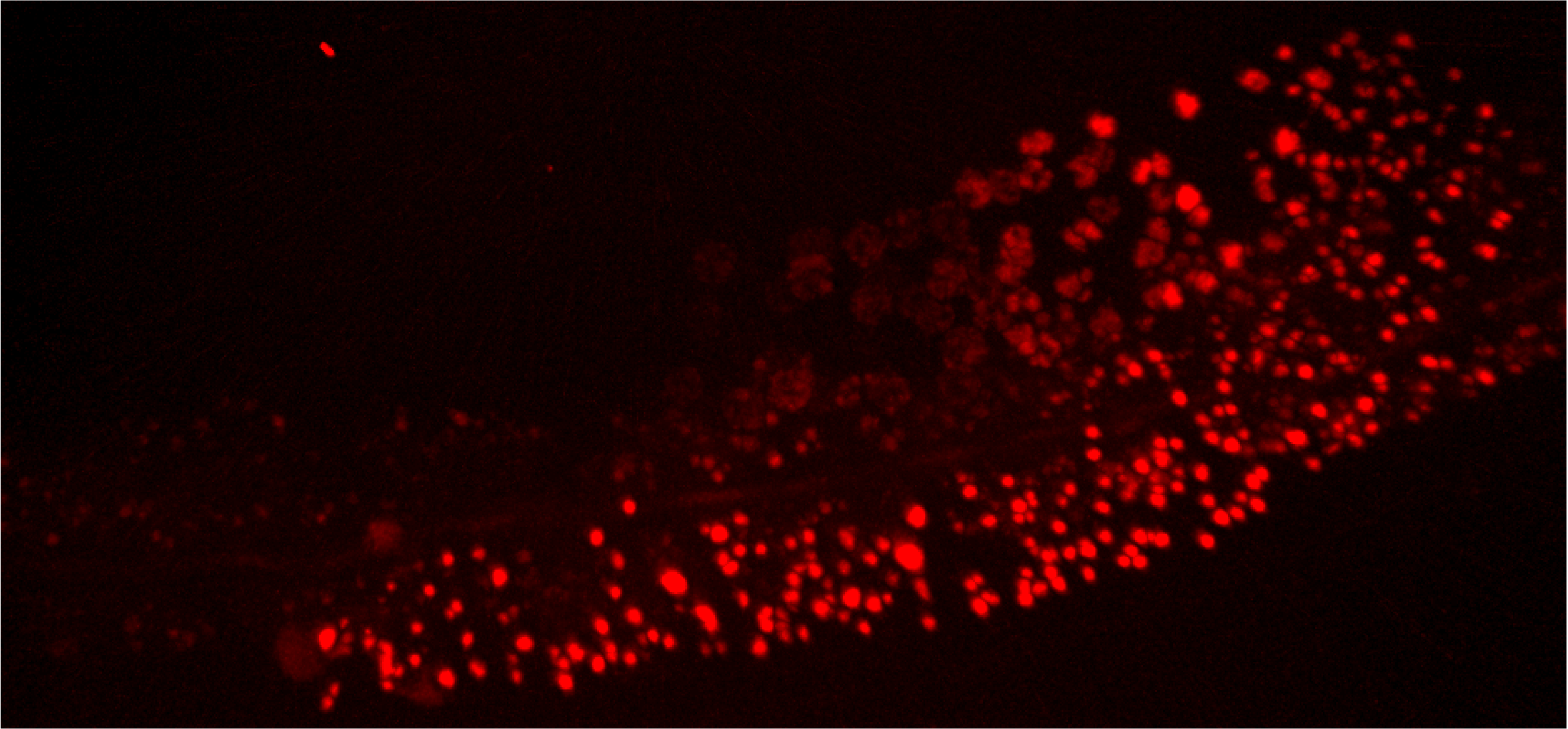

Supplement: Supplementary file 8 — Source data Fig. 6 [file 44318_2024_197_MOESM8_ESM.zip › SD figure 6/6H/6H.tif]

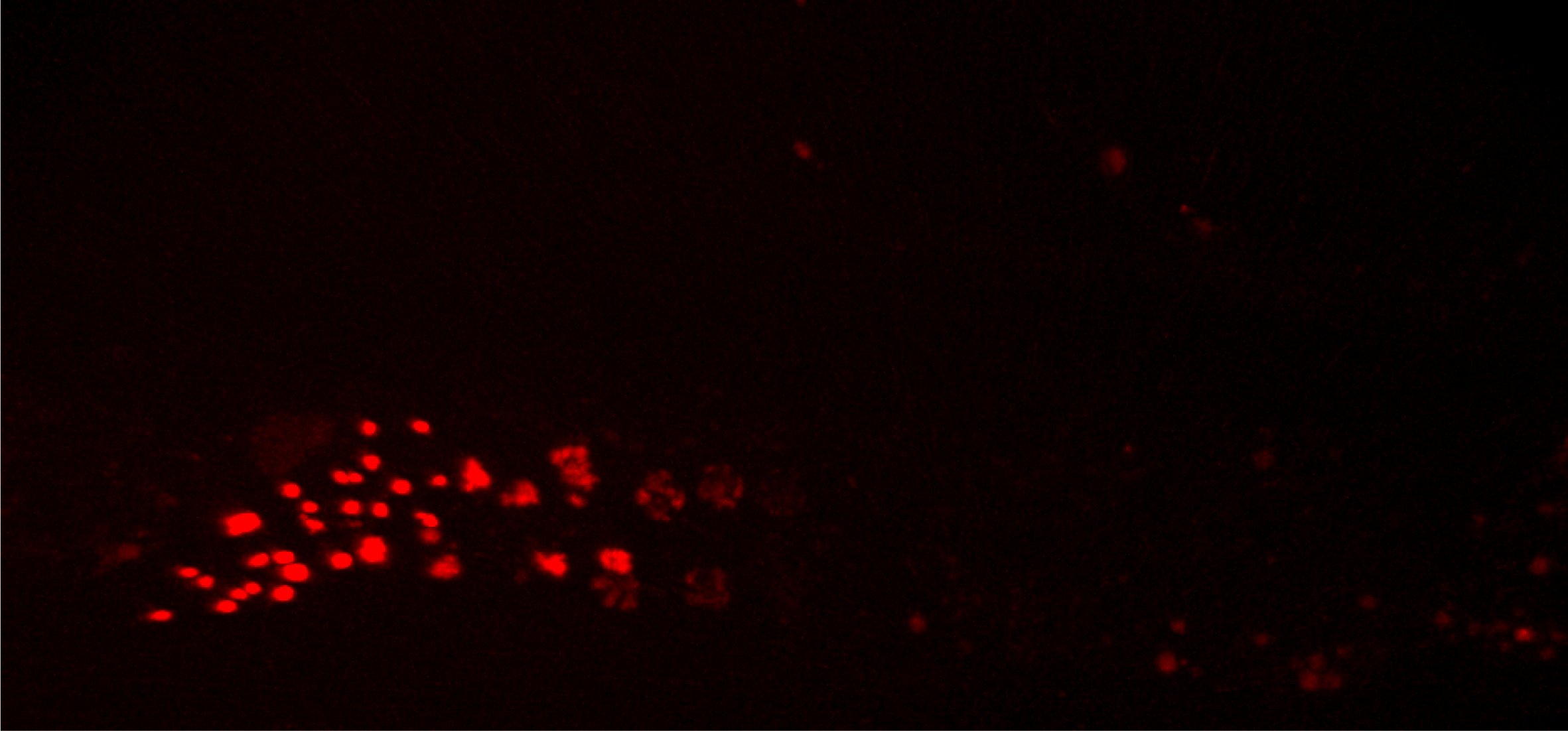

Supplement: Supplementary file 8 — Source data Fig. 6 [file 44318_2024_197_MOESM8_ESM.zip › SD figure 6/6I/6I.tif]

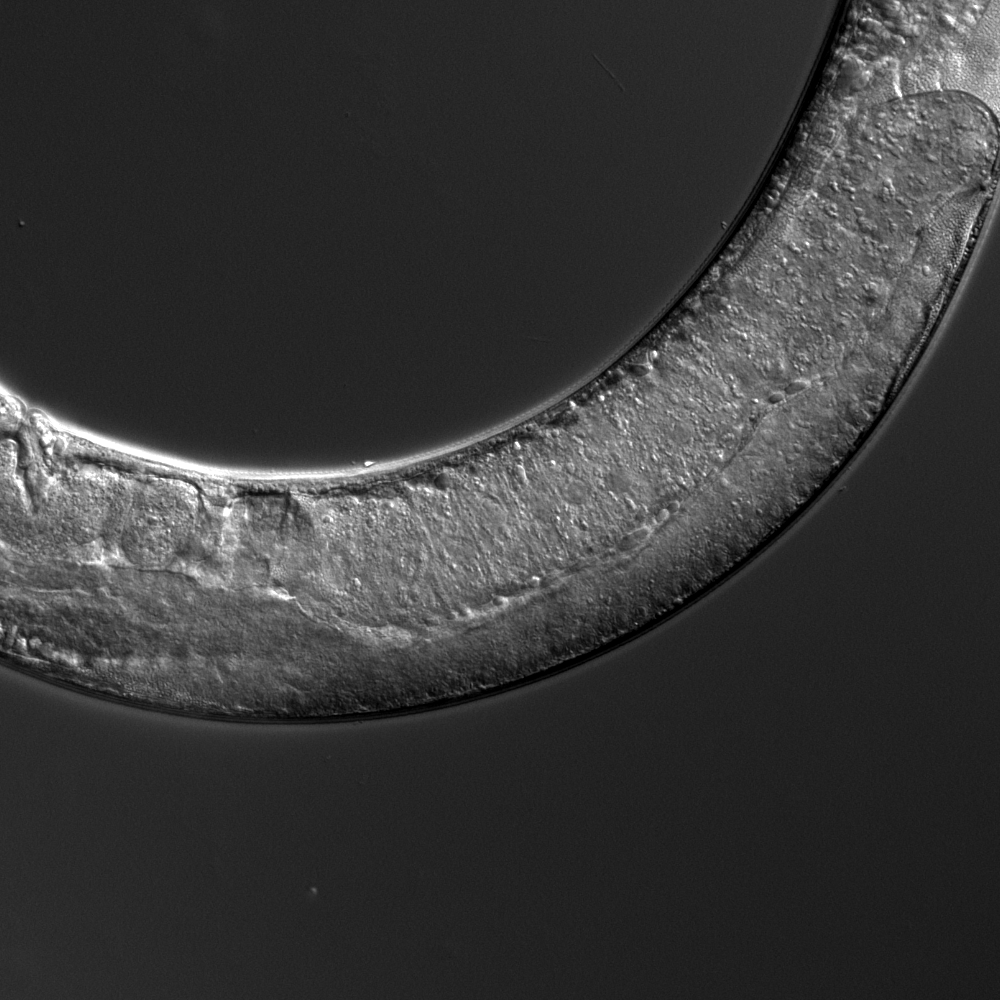

Supplement: Supplementary file 8 — Source data Fig. 6 [file 44318_2024_197_MOESM8_ESM.zip › SD figure 6/6M/6M.tif]

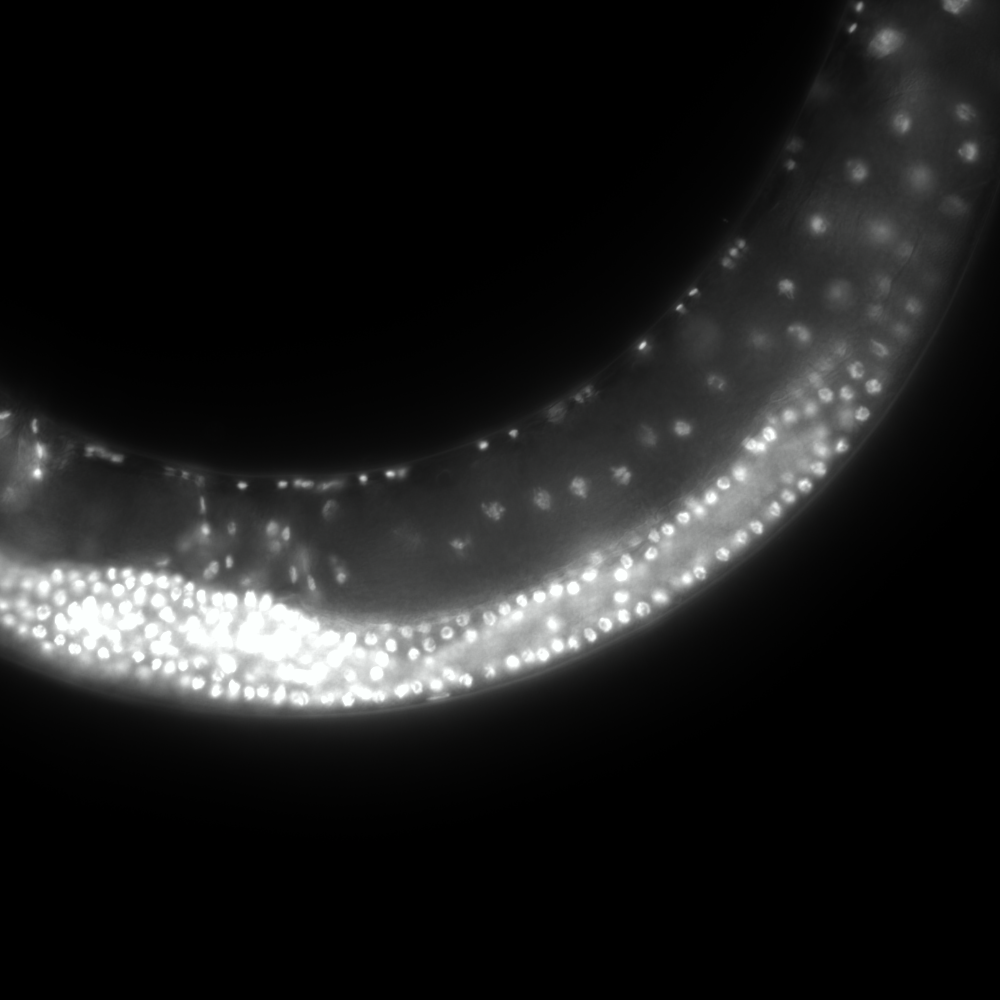

Supplement: Supplementary file 8 — Source data Fig. 6 [file 44318_2024_197_MOESM8_ESM.zip › SD figure 6/6N/6N.tif]

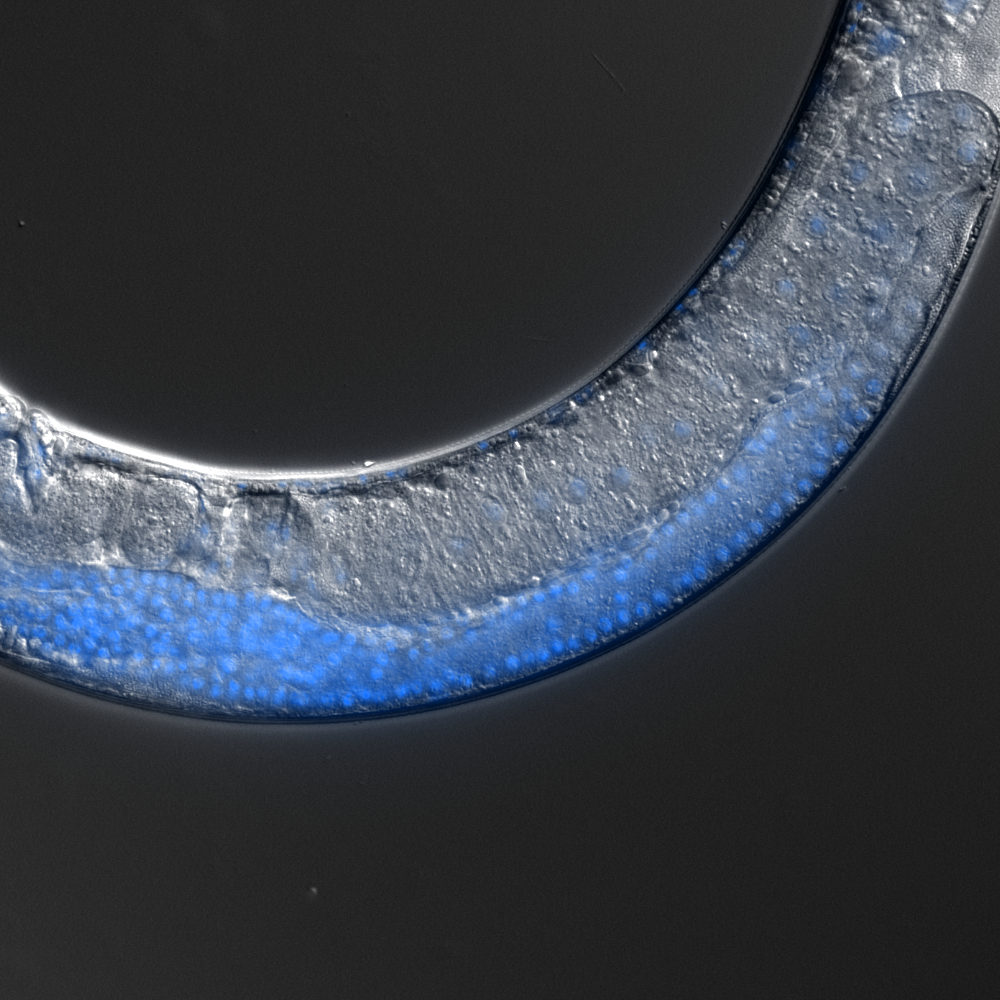

Supplement: Supplementary file 8 — Source data Fig. 6 [file 44318_2024_197_MOESM8_ESM.zip › SD figure 6/6O/6O.tif]

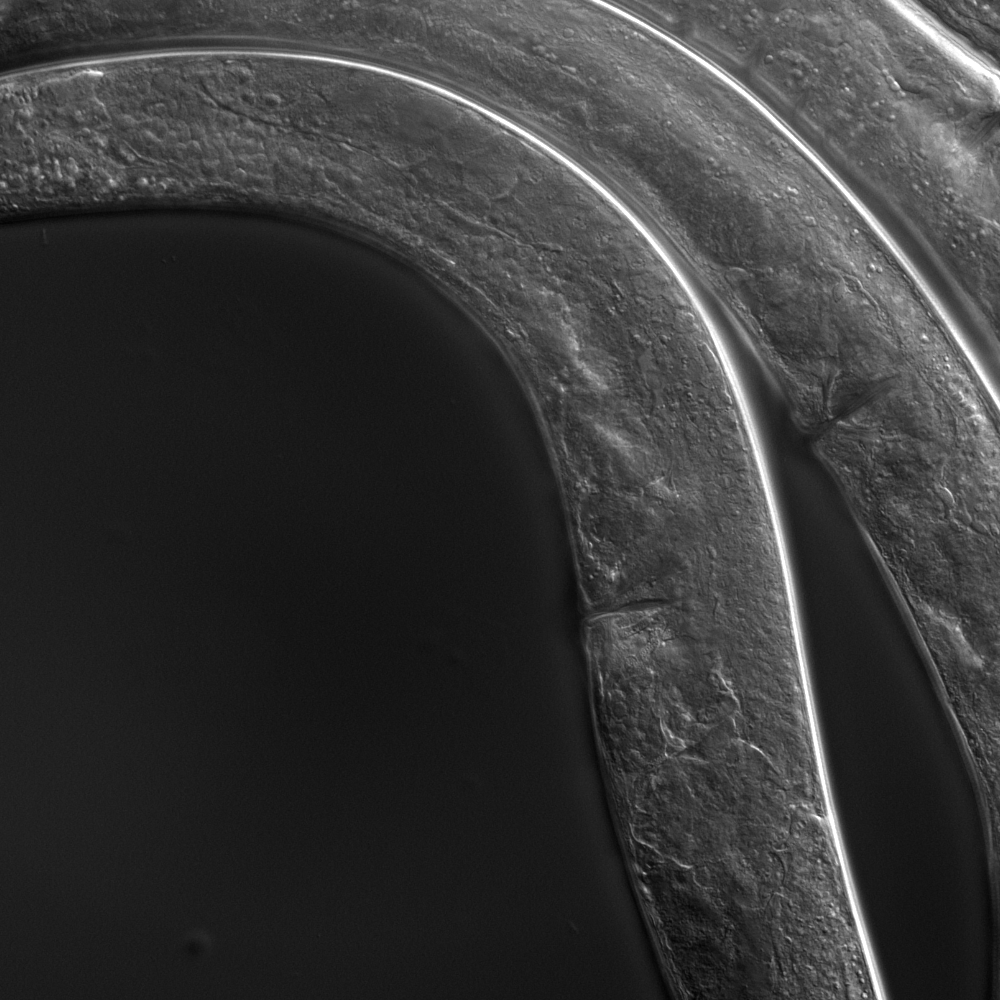

Supplement: Supplementary file 8 — Source data Fig. 6 [file 44318_2024_197_MOESM8_ESM.zip › SD figure 6/6P/6P.tif]

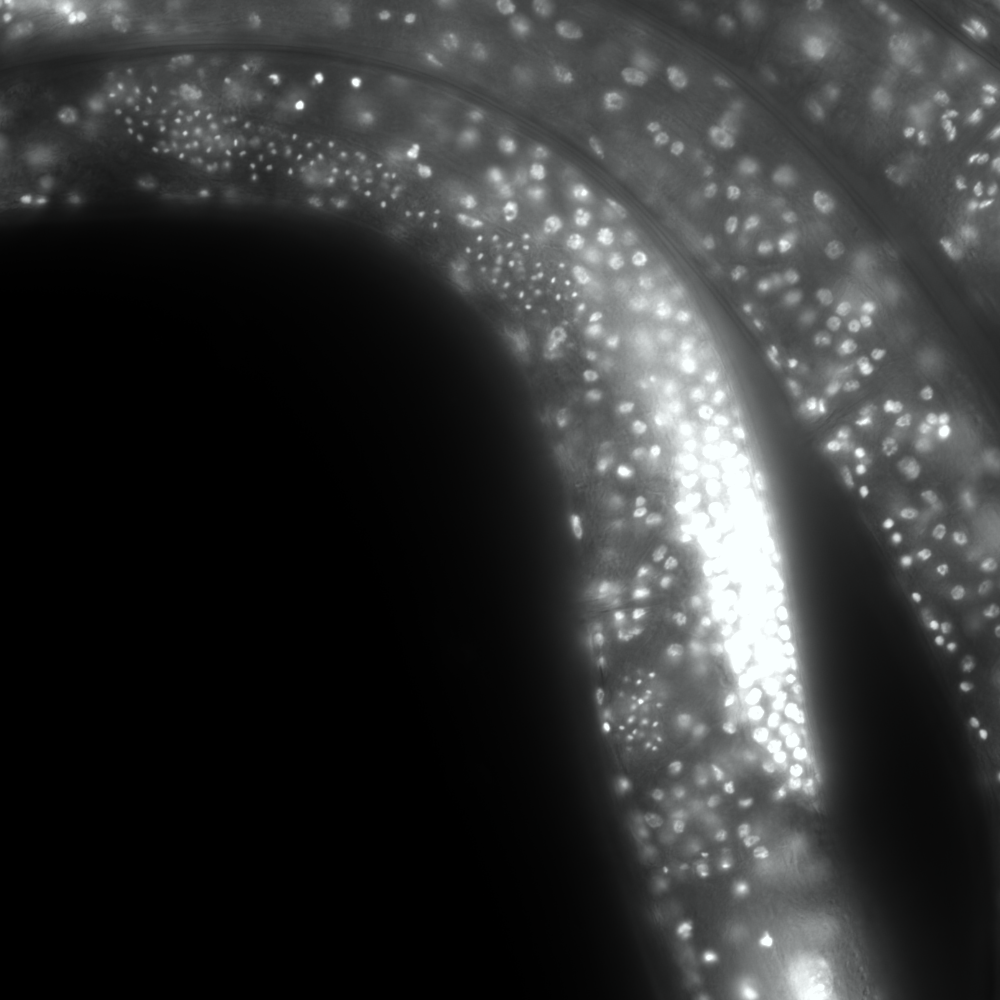

Supplement: Supplementary file 8 — Source data Fig. 6 [file 44318_2024_197_MOESM8_ESM.zip › SD figure 6/6Q/6Q.tif]

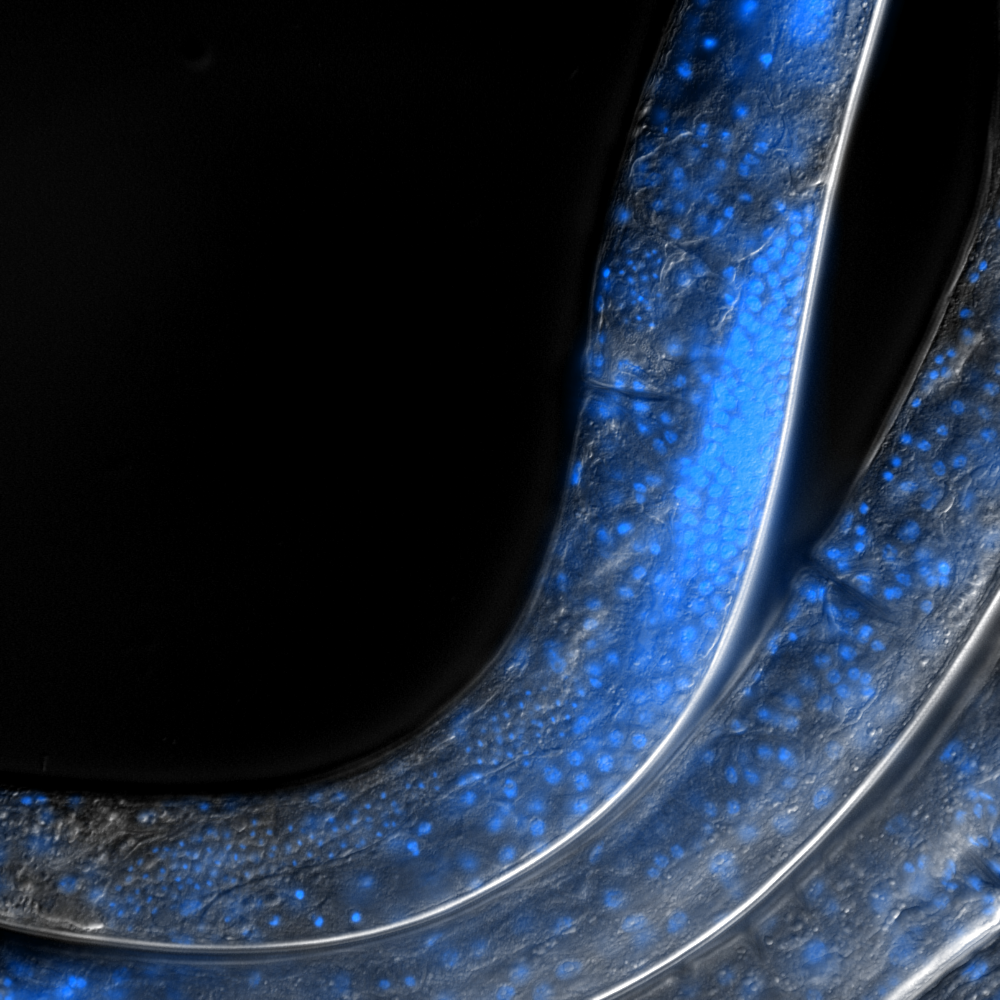

Supplement: Supplementary file 8 — Source data Fig. 6 [file 44318_2024_197_MOESM8_ESM.zip › SD figure 6/6R/6R.tif]
