# Supplementary material for: Identification of BiP as a temperature sensor mediating temperature-induced germline sex reversal in C. elegans
Source: EMBO J. 2024 Aug 12;43(18):4020–48. doi: 10.1038/s44318-024-00197-z (PMC11405683; doi:10.1038/s44318-024-00197-z)
Supplement: Supplementary file 9 — Expanded View Figures [file 44318_2024_197_MOESM9_ESM.pdf]

Expanded View Figures

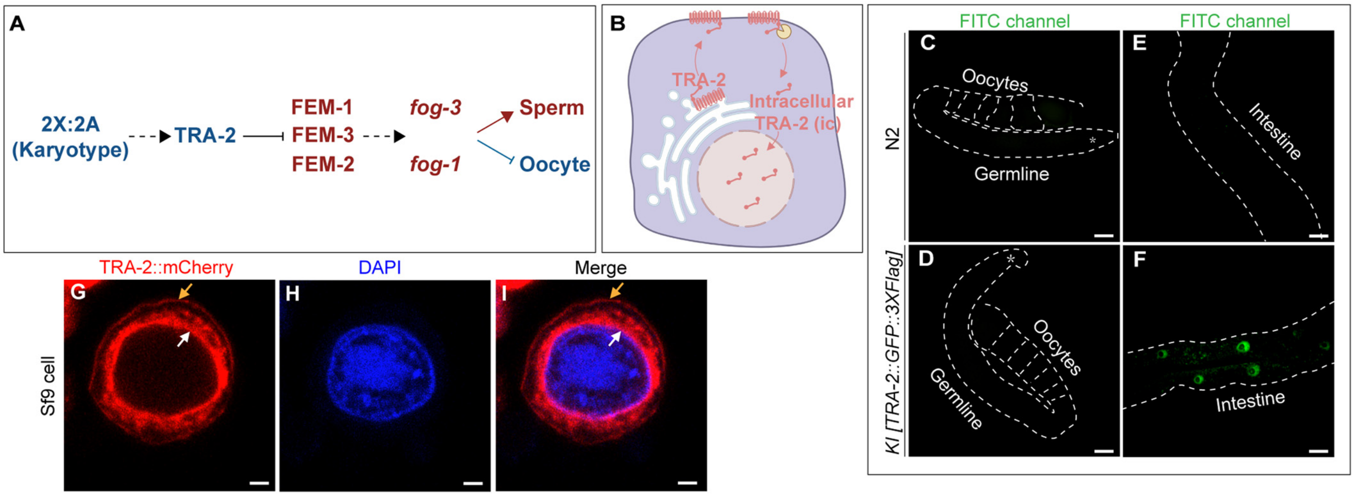

Figure EV1. The analysis of the TRA-2 expression pattern and subcellular localization.

(**A**) Model of the karyotype-mediated germline sex determination pathway in *C. elegans*, adopted from previous studies (Ellis and Schedl, 2007). The karyotype (2X:2A; ratio of X chromosomes to autosomes in *C. elegans* hermaphrodites) is the primary signal that modulates germline sex by regulating *tra-2*, which encodes a transmembrane protein functioning upstream of the FEM proteins. The relative ratio of TRA-2 to FEM-3 expression is critical for determining germline sex. In response to the upstream signal, the transcription of *fog-3*, encoding a member of the Tob protein family, is regulated to modulate germ cell fate. The genes in red promote sperm fate and the genes in blue drive oocyte fate. (**B**) A diagram illustrating the cellular behavior of TRA-2, summarized from previous studies (Mapes et al, 2010b; Shimada et al, 2006; Sokol and Kuwabara, 2000). The full-length transmembrane TRA-2 is synthesized and folded at the ER membrane. It is then trafficked to the plasma membrane, where it is cleaved. This cleavage releases a short intracellular C-terminal fragment that can translocate to the nucleus. (**C–F**) Representative images showing the expression pattern of the native-expressed TRA-2::GFP. The *KI [TRA-2::GFP::3×Flag]* strain, where the *GFP::3×Flag* was knocked in to the C-terminus of native *tra-2*, was used to determine the expression pattern of *tra-2*. GFP fluorescence can be observed in the nuclei of intestinal cells (**F**). In contrast, the TRA-2::GFP signal was not visible in the germline (**D**). The asterisk (*) indicates the distal end of the germline and white dashed lines outlined the germline arms and intestine. N2 served as a control to exclude interference from autofluorescence. $n = 60$ worms. Scale bar: 20 μm. (**G–I**) Micrographs showing the perinuclear and cell peripheral TRA-2 in Sf9 cells. The worm TRA-2 fused with mCherry was expressed in Sf9 insect cells to determine the subcellular localization of TRA-2. The white arrow indicates the TRA-2 signal in the perinuclear region, and the orange arrow indicates the plasma membrane-localized TRA-2. $n = 30$ cells were evaluated. Scale bar: 3 μm.

 © The Author(s)

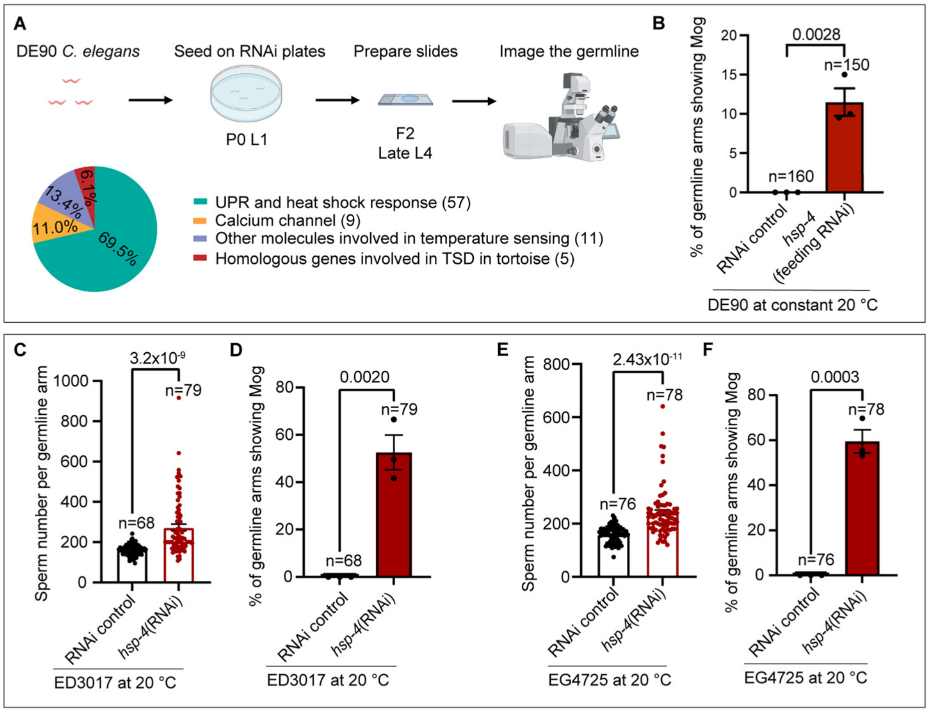

G

Genotype	Phenotype
hsp-3(lf)	No Mog (n>100)
hsp-4(lf)	No Mog (n>100)
hsp-3(lf/+);hsp-4(lf/+)	No Mog (n=35)
hsp-3(lf);hsp-4(lf/+)	Mostly lethal; the F1 survivals from *hsp-3(lf/+);hsp-4(lf/+)* mother showing no Mog (n=6)
hsp-3(lf/+);hsp-4(lf)	Mog (n=31)
hsp-3(lf);hsp-4(lf)	Mostly lethal; the F1 survivals from *hsp-3(lf/+);hsp-4(lf/+)* mother showing Mog (n=2)

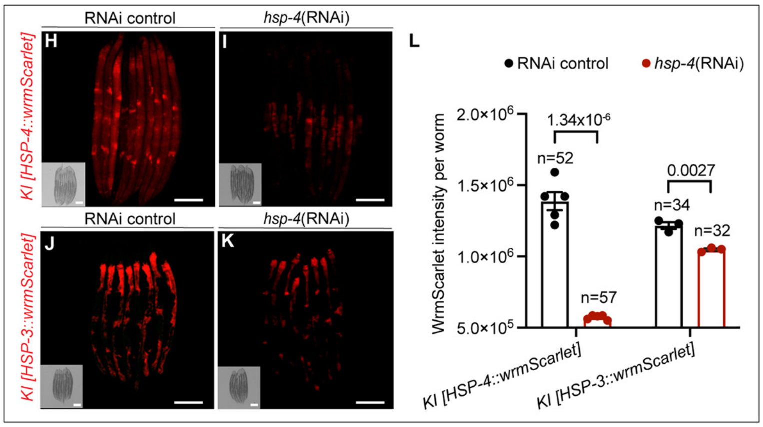

Figure EV2. Uncovering the role of BiP in germ cell fate determination through a RNAi screen and the redundant role of HSP-3 and HSP-4 BiPs in driving female germline sex.

(**A**) Workflow of the RNAi screen for identifying the potential thermosensitive genes that regulate germline sex, and a pie chart showing the categories of the genes included in the screen. The RNAi screen was performed by feeding at a constant 20 °C using DE90 strain. (**B**) Graph showing that in the screen described in (**A**), *hsp-4(RNAi)* by feeding caused Mog in a subset of hermaphrodites. The DE90 strain receiving *hsp-4*(RNAi) treatment was analyzed to determine the percentage of germline arms showing Mog. *P* value = 0.0028. (**C–F**) Bar graphs showing strong Mog caused by *hsp-4*(RNAi) in the wild-isolated *C. elegans* ED3017 and EG4725 strains cultured at a constant 20 °C. The *hsp-4*(RNAi) experiment was performed by microinjection with corresponding dsRNA. The quantitative analyses of the sperm number are shown in (**C**, **E**). With *hsp-4*(RNAi) treatment, 52.6% and 59.5% of germline arms displayed Mog in ED3017 (**D**) and EG4725 (**F**), respectively. *P* value $= 3.2 \times 10^{-9}$ (**C**), *P* value = 0.0020 (**D**), *P* value $= 2.43 \times 10^{-11}$ (**E**), *P* value = 0.0003 (**F**). (**G**) A table showing that the HSP-3 and HSP-4 BiPs function redundantly to regulate germline sex determination. The progeny of *hsp-3(lf/+);hsp-4(lf/+)* worms were scored to analyze Mog in the *hsp-3(lf/+);hsp-4(lf/+)*, *hsp-3(lf/+);hsp-4(lf)*, *hsp-3(lf);hsp-4(lf/+) and hsp-3(lf);hsp-4(lf)* worms. "*n*" indicates the number of worms evaluated. (**H–L**) Micrographs and a corresponding bar graph indicating that *hsp-4*(RNAi) can knock down both *hsp-3* and *hsp-4*. *hsp-4*(RNAi) was performed by the feeding method. The fluorescence signals of the native HSP-4::wrmScarlet and native HSP-3::wrmScarlet proteins in worms were analyzed after control(RNAi) or *hsp-4*(RNAi) treatment. The quantification of the mean fluorescence intensity of HSP-4::wrmScarlet in (**H**, **I**) and HSP-3::wrmScarlet per worm in (**J**, **K**) is shown in (**L**). *P* value $= 1.34 \times 10^{-6}$, *P* value = 0.0027 in (**L**) (from left to right). Scale bar:100 µm. For the statistics in this Figure, the data are presented as the means ± SEMs. Each dot represents the sperm number in one germline arm in (**C**, **E**), the percentage of germline showing Mog each replicate in (**B**, **D**, **F**) and the mean fluorescence intensity of wrmScarlet in a worm in each replicate in (**L**). Statistical significance was performed by unpaired *t* test, three biological independent replicates.

© The Author(s)

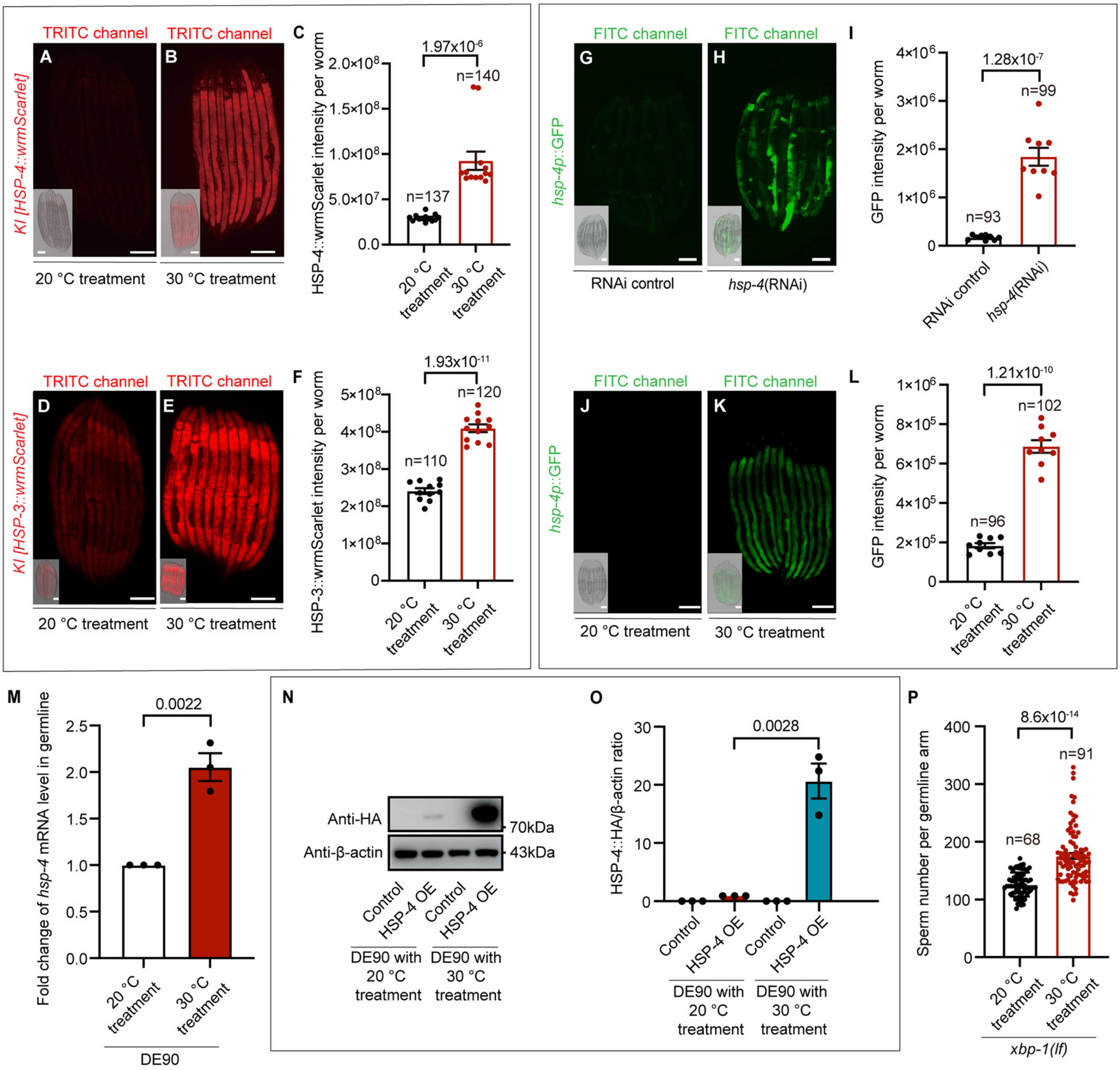
TRITC channel
TRITC channel
A
B
KI [HSP-4::wrmScarlet]
20 °C treatment
30 °C treatment
C
HSP-4::wrmScarlet intensity per worm
1.97x10-6
n=140
n=137
20 °C treatment
30 °C treatment
FITC channel
FITC channel
G
H
hsp-4p::GFP
RNAi control
hsp-4(RNAi)
I
GFP intensity per worm
1.28x10-7
n=99
n=93
RNAi control
hsp-4(RNAi)
D
E
KI [HSP-3::wrmScarlet]
F
HSP-3::wrmScarlet intensity per worm
1.93x10-11
n=120
n=110
J
K
L
1.21x10-10
n=102
n=96
M
Fold change of hsp-4 mRNA level in germline
0.0022
DE90
N
Anti-HA
Anti-β-actin
70kDa
43kDa
Control
HSP-4 OE
DE90 with 20 °C treatment
DE90 with 30 °C treatment
O
HSP-4::HA/β-actin ratio
0.0028
P
Sperm number per germline arm
8.6x10-14
n=91
n=68
xbp-1(lf)

 © The Author(s)

◀ **Figure EV3. The responsiveness of BiP to the temperature change.**

(A–F) Micrographs and bar graphs showing the induction of BiPs by warmer temperature. After treatment with the indicated temperatures, the BiPs' levels were analyzed by determining the fluorescence intensity of native HSP-4::wrmScarlet and HSP-3::wrmScarlet. The quantification of the mean fluorescence intensity of HSP-4::wrmScarlet and HSP-3::wrmScarlet per worm in (**A**, **B**) and (**D**, **E**) is shown in (**C**, **F**), respectively. *P* value $= 1.97 \times 10^{-6}$ (**C**), *P* value $= 1.93 \times 10^{-11}$ (**F**). Scale bar: 100 µm. **(G–I)** Fluorescent images and a corresponding bar graph showing that *hsp-4*(RNAi) induces *hsp-4p*::GFP expression. The transcription of *hsp-4* was obviously induced in the transcriptional fusion *hsp-4p::GFP* reporter worms treated with *hsp-4(RNAi)* by feeding at a constant 20 °C. The quantification of the mean fluorescence intensity of GFP per worm in (**G**, **H**) is shown in (**I**). *P* value $= 1.28 \times 10^{-7}$ (**I**). Scale bar: 100 µm. **(J–L)** Micrographs and corresponding quantitative analyses showing the induction of *hsp-4* transcription in the worms treated with the warmer temperature. The *hsp-4p::GFP* signal was markedly enhanced by 30 °C treatment (**J**, **K**), which further support the notion that warmer temperature induces a reduction in the available BiP levels. Quantification of the mean fluorescence intensity of GFP per worm is shown in the bar graph (**L**). Scale bar: 100 µm. *P* value $= 1.21 \times 10^{-10}$ (**L**). **(M)** qPCR analysis indicating the induction of *hsp-4* transcription in the germline after warmer temperature treatment. After the treatment with indicated temperatures, the mRNA of *hsp-4* was isolated from the dissected germline arms and then analyzed by qPCR.
P value $= 0.0022$. **(N, O)** Western blot images and the corresponding quantification showing the successful overexpression of HSP-4. The *Is [hsp-16.41::HSP-4::Flag::HA]* was created in DE90 background. The indicated worms were grown at 20 °C for 30 h, and then the worms in 30 °C treatment group were shifted to the 30 °C for 12 h. The corresponding quantitative analysis was presented in (**O**). *P* value $= 0.0028$ (**O**). **(P)** A bar graph showing that excess sperm were produced in the germline of the *xbp-1(lf)* mutant in response to the 30 °C treatment. The sperm number in each germline arms were counted by DAPI staining. *P* value $= 8.6 \times 10^{-14}$. The data are presented as the means ± SEMs. Each dot represents the mean fluorescence intensity of wrmScarlet in a worm in each replicate in (**C**, **F**), the mean fluorescence intensity of GFP in a worm in each replicate (**I**, **L**), the fold change in *hsp-4* mRNA levels in each replicate in (**M**), the value of HSP-4::Flag::HA/beta-actin ratio in each replicate in (**O**) and the sperm number in one germline arm in (**P**). Statistical analyses were performed by unpaired t test; three biological independent replicates.

© The Author(s)

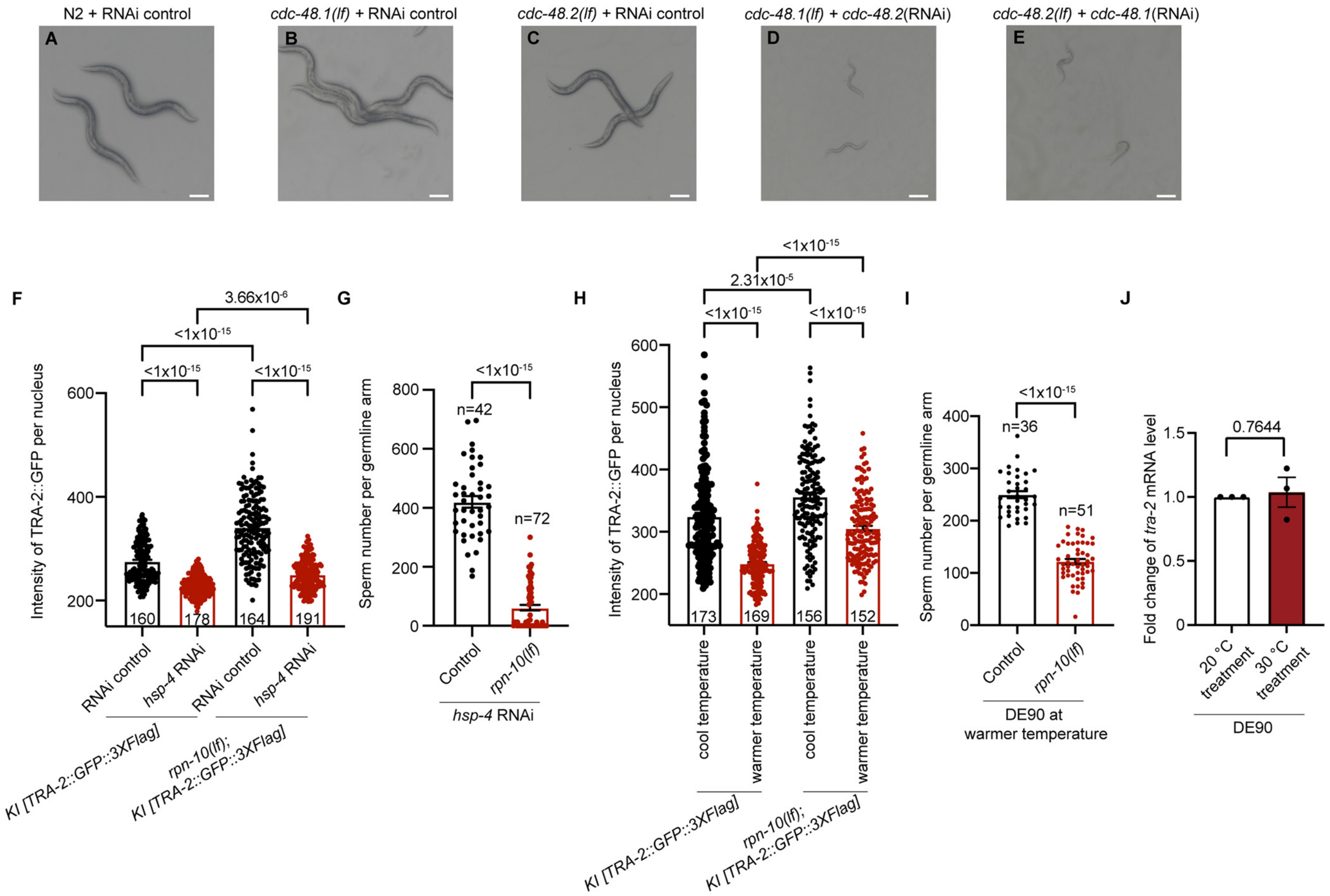

Figure EV4. CDC-48.1 and CDC-48.2 function redundantly to modulate *C. elegans* development.

(**A–E**) Images showing that *cdc-48.1(lf)* with *cdc-48.2*(RNAi) treatment and *cdc-48.2(lf)* with *cdc-48.1*(RNAi) treatment resulted in severe developmental defects. Synchronized N2, *cdc-48.1(lf)* and *cdc-48.2(lf)* worms with indicated feeding RNAi treatment, cultured at a constant 20 °C, were scored for the developmental defects. The number of nematodes assessed in each group exceeds 50. Scale bar: 100 μm. (**F**) Bar graphs illustrating that the decrease in TRA-2 expression induced by *hsp-4*(RNAi) was suppressed by the *rpn-10(lf)* mutations. The RNAi experiments were performed by feeding. *P* value $< 1 \times 10^{-15}$, *P* value $< 1 \times 10^{-15}$, *P* value $= 3.66 \times 10^{-6}$, *P* value $< 1 \times 10^{-15}$ (from left to right). (**G**) Bar graphs indicating the suppression of *hsp-4*(RNAi)-induced Mog by the *rpn-10(lf)* mutations. The *rpn-10(lf)* mutations were generated in the DE90 strain by the CRISPR–Cas9 method, and the worms were treated with RNAi against *hsp-4* by microinjection. *P* value $< 1 \times 10^{-15}$. (**H**) A bar graph indicating that warmer temperature (30 °C)-induced decrease in TRA-2 levels was suppressed by the *rpn-10(lf)* mutation. Due to the temperature sensitivity of *rpn-10(lf)* (Shimada et al, 2006), the worms were cultured at a constant 25 °C (cool temperature) to observe the mutant phenotype. *P* value $< 1 \times 10^{-15}$, *P* value $= 2.31 \times 10^{-5}$, *P* value $< 1 \times 10^{-15}$, *P* value $< 1 \times 10^{-15}$ (from left to right). (**I**) Bar graphs indicating that the *rpn-10(lf)* mutation significantly suppresses the warmer temperature (30 °C) treatment-induced excess sperm production. The *rpn-10(lf)* mutation was generated in the DE90 strain using the CRISPR–Cas9 method. *P* value $< 1 \times 10^{-15}$. (**J**) qPCR analysis indicating that *tra-2* transcription is not affected by the temperature elevation. DE90 worms with the indicated temperature treatments were collected for qPCR analyses. *P* value $= 0.7644$. The data are presented as the means ± SEMs. Each dot in the bar graph corresponds to the mean intensity of native TRA-2::GFP per nucleus in (**F**, **H**), the sperm number per germline arm in (**G**, **I**) and the fold change in *tra-2* mRNA levels in each replicate in (**J**). Statistical analyses were performed by unpaired *t* test in (**G**, **I**, **J**) and one-way ANOVA in (**F**, **H**). Three biological independent replicates were conducted.

© The Author(s)

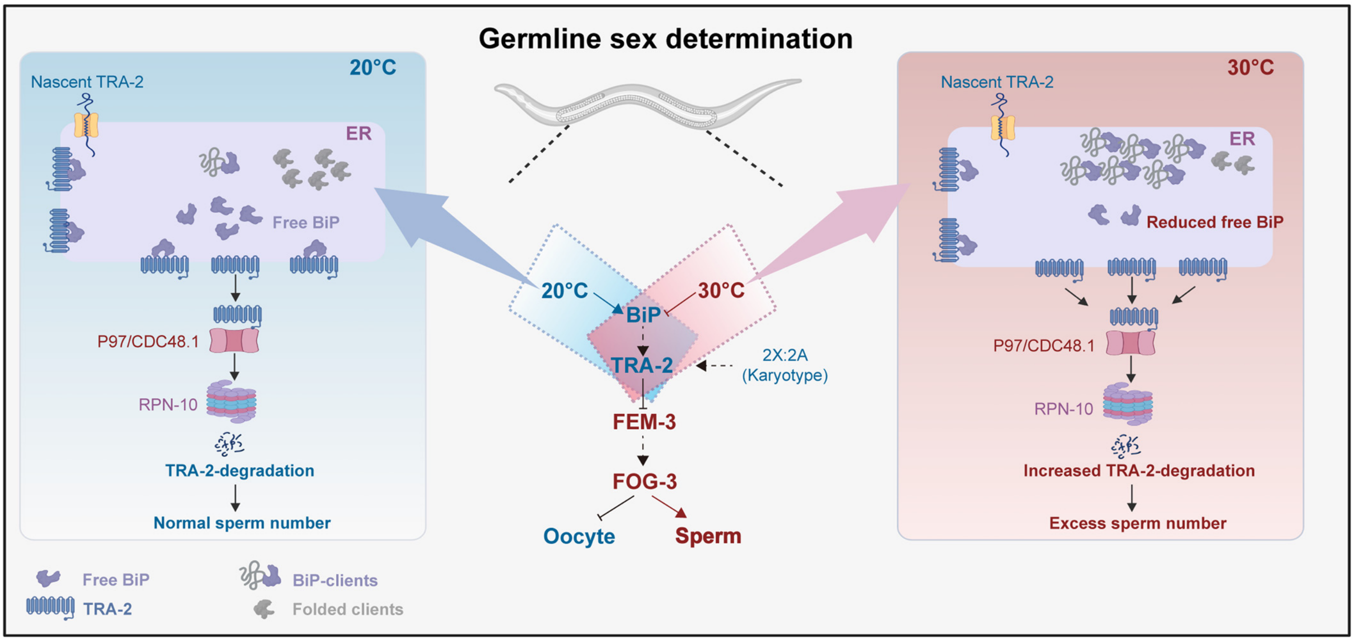

Figure EV5. A proposed model of BiP-mediated temperature-dependent germline sex reversal in *C. elegans*.

Model of the role of the thermosensitive ER chaperone BiP in translating temperature cues into the germline sex determination signal. The karyotype was previously shown to modulate TRA-2 expression to determine germline sex (Ellis and Schedl, 2007). In this study, we found that the thermosensitive regulator BiP senses and translates temperature cues into a germline sex regulatory signal by modulating the level of TRA-2. Specifically, we revealed that BiP is required for driving female germline fate by preventing the ERAD-mediated degradation of TRA-2, which acts as an important posttranslational mechanism to modulate germline sex determination. As temperature has been shown to directly affect protein folding (Day et al, 2002), the amount of available BiP is altered in response to alterations in temperature via detection of the resultant fluctuations in ER protein folding. When worms are exposed to the warmer temperature (30 °C), the amount of available BiP is decreased, which causes a decrease in TRA-2 expression to promote male germline fate. Therefore, BiP acts as a temperature sensor that mediates germline sex determination. Taken together, these findings indicate that the temperature, via a BiP-mediated process, and the karyotype co-regulate the key regulator of the sex determination pathway, TRA-2, to modulate germline sex in *C. elegans*, thus enabling the coexistence of TGSD and GGSD.

© The Author(s)
